# Priming of human iPSCs with doxorubicin causes genome-wide transcriptional reprogramming and functional impairments in iPSC-derived cardiomyocytes

**DOI:** 10.1038/s41536-026-00513-7

**Published:** 2026-10-10

**Authors:** Michelle Westerhoff, Roohallah Ghodrat, Nahal Brocke-Ahmadinejad, Yulia Schaumkessel, Karl Köhrer, Arif Dönmez, Timo Dellmann, David Pla-Martín, Christian Henninger, Andrea Borchardt, Lluís Enjuanes-Ruiz, Julia Tigges, Katharina Koch, Gerhard Fritz, Arun Kumar Kondadi, Andreas S. Reichert

**Affiliations:** 1 Institute of Biochemistry and Molecular Biology I, Medical Faculty and University Hospital Düsseldorf, Heinrich-Heine-University Düsseldorf, Düsseldorf, Germany; 2 Genomics and Transcriptomics Laboratory, Biological and Medical Research Center (BMFZ), Heinrich-Heine-University Düsseldorf, Düsseldorf, Germany; 3 IUF - Leibniz Research Institute for Environmental Medicine, Düsseldorf, Germany; 4 DNTOX GmbH, Düsseldorf, Germany; 5 Advanced Light Microscopy Core Facility (Ad-Light), Medical Faculty, Heinrich-Heine-University Düsseldorf, Düsseldorf, Germany; 6 Institute of Toxicology, Medical Faculty and University Hospital Düsseldorf, Heinrich-Heine-University Düsseldorf, Düsseldorf, Germany; 7 Present Address: Institute of Cardiovascular Physiology, Medical Faculty and University Hospital Düsseldorf, Heinrich-Heine-University Düsseldorf, Düsseldorf, Germany

**Keywords:** Cardiology, Cell biology, Stem cells

## Abstract

How Doxorubicin (Dox) mediated damage to stem cells or cardiomyocytes contributes to cardiotoxicity and cardiac regeneration is unclear. Using a human iPSC-based model together with corresponding iPSC-derived cardiomyocytes (iCMs) revealed that iPSCs are much more sensitive against single short pulse-treatments with Dox than iCMs. Major mitochondrial impairments such as increased mitochondrial fragmentation, loss of mitochondrial membrane potential, reduced ATP levels, and altered mitochondrial DNA (mtDNA) organization, yet neither increased ROS formation, nuclear DNA damage response, nor apoptosis were detected in Dox-treated iPSCs. Whole genome transcriptome profiling revealed major differences in the transcriptional response to Dox including downregulation of genes regulating cellular homeostasis and energy metabolism. Dox exposure at the iPSC stage, termed priming here, shifts the global transcriptional landscape of iCMs towards reduced cardiac muscle regeneration, energy metabolism, muscle contraction, and increased fibrosis. Functionally, priming caused iCMs to ‘memorize’ this early Dox treatment resulting in reduced ATP levels, altered mtDNA organization, and in impaired contractility and oscillating Ca^2+^ handling. We propose that Dox-induced mitochondrial dysfunction and transcriptional priming in stem cells alters durably cardiac and bioenergetic properties of its progeny which may impair the regenerative capacity after differentiation. This may be of particular relevance for children and teenagers undergoing chemotherapy.

## Introduction

The anthracycline doxorubicin (Dox) finds wide applications in the chemotherapeutic treatment of solid tumors (e.g., neuroblastoma, ovary, gastrointestinal, and breast tumors) and hematological malignancies (e.g., lymphatic and myeloid tumors) in adults as well as in children^1^. A major clinical challenge during, and years after chemotherapy, is the onset of cardiotoxicity resulting from cancer therapy with Dox^2,3^. The manifestation of Dox-induced cardiotoxicity depends on the cumulative dose and timing of Dox administration, yet also shows high inter-individual variability in patients^4^. Dysregulation of cardiac electrophysiological features within hours to weeks after first administration of Dox has been well documented^5,6^. Side effects of Dox, such as congestive heart failure, a reduction in the left ventricular ejection fraction and serious arrhythmias, may occur within the first year of Dox treatment but often manifest much later, even 10 years after the initial application^2,3,7^. In particular, children, women, and elderly patients are prone to late-onset cardiac problems. The underlying mechanisms of these acute and chronic cardiotoxic effects are still unresolved and are a matter of intense research^8–10^.

The heart as a contractile muscle apparatus is reliant on the functionality of cardiomyocytes which represent ~30–35% of the total cellular mass in the human heart^11^. Homeostasis of these cells is believed to determine the sensitivity of patients against Dox treatment^12–14^. As cardiomyocytes are terminally differentiated cells, it has been proposed that Dox-induced dysfunction of these cells cannot solely be dependent on the well-known inhibition of DNA replication by Dox^15^. In addition, the formation of reactive oxygen species (ROS), the specific inhibition of topoisomerases including topoisomerase IIβ in mitochondria, and inhibition of autophagic processes have been implicated in Dox-induced cardiotoxicity^16,17^. Besides the impairment of cytosolic and nuclear processes, mitochondria have been described to play a pivotal role in the induction of cardiotoxic effects upon Dox exposure^18–20^. Given an estimated turnover rate of mitochondrial proteins in the heart of about 16–18 days, Dox-induced alterations may persist and propagate over time rather than being eliminated from the pool rapidly^21,22^. Yet, in most studies mitochondrial dysfunction has been observed upon treatment with comparably high treatment doses which may not completely reflect the actual clinical situation. In patients, Dox plasma concentrations drop rapidly within 1 h after short-time infusion of Dox to values between 25 and 250 nM^23^. As a consequence, previous in vitro approaches observing mitochondrial dysfunction under application of high Dox doses likely involved multiple damaging mechanisms (e.g.,: nuclear and mitochondrial DNA damage, ROS production, inhibition of autophagic processes, etc.) prompting us to establish a model using low Dox concentrations allowing us better to decipher the role of mitochondria as mediators of cardiotoxicity. In general, impairment of mitochondrial quality control pathways is clearly linked to cardiac failure during ageing and cardiovascular diseases highlighting the central role of mitochondria in cardiac function^24^. While Dox-induced mitochondrial dysfunction directly affects the viability and function of cardiomyocytes, the long-term impact of such a damage may be further augmented by the low ability of the heart to effectively replace damaged cardiomyocytes. In fact, there is tremendous interest in the last few decades to understand how regeneration of the heart after cardiac damage can be promoted^25^.

During development of the mammalian heart, cardiac progenitor cells differentiation into cardiomyocytes (CMs) and local proliferation of existing CMs is known to decline strongly around the time of birth followed by a period of hypertrophy of cardiomyocytes^11^. This is in line with the general view that the adult heart is an organ of low regenerative capacity^26^ showing a low rate of ~1% turnover of CMs per year^27–29^. This is age-dependent as the annual percentage of CMs replaced is highest in the first decade of life (declining from 5% for newborns to ~2% at the age of 10 years), reaching ~0.8% after 20 years of age and ~0.3% in 75-year-old adults based on ^14^C-isotope tracing studies in humans^14^. Moreover, upon cardiac damage to a low degree damaged CMs are replaced as several studies in animal models clearly showed that e.g., after ischemia newly formed CMs are found at low levels and are enriched in ischemic regions compared to non-infarcted regions of the myocardium^25,28,30^. Despite the fact that several fraudulent and partly retracted studies from one group claimed a much higher regenerative capacity of the mammalian adult heart under physiological conditions and in particular upon transplantation of bone-marrow derived hematopoietic stem cells (e.g., c-kit+ cells), one still needs to emphasize that numerous preclinical and clinical studies performed in other labs have shown that administration of isolated non-cardiac or stromal cardiac cells show beneficial effects for the damaged heart^25,30–33^. These studies demonstrated a low degree of cardiac tissue renewal at least in part originating from transplanted cells and it is critically discussed whether this by itself is sufficient to explain the observed beneficial effects on cardiac function. Currently, the view prevails that positive paracrine effects derived from transplanted cells (e.g., mesenchymal stem cells (MSCs), hematopoietic stem cells or endothelial cell precursors) on existing CMs are of particular relevance in this context^25^. Overall, promising strategies, including stem cells to regenerate the damaged heart are widely explored. This encompasses approaches to promote proliferation of existing CMs via dedifferentiation and redifferentiation as well as cell-based delivery strategies, including allogeneic or autologous transplantation of CMs derived from pluripotent stem cells (PSCs) or from iPSCs^25^.

We hypothesize that mitochondrial dysfunction could contribute to both long-lasting damage of existing cardiomyocytes as well as to suppressed cardiac regeneration which might play a pivotal role in the manifestation of long-term cardiotoxic effects upon chemotherapeutic treatment of patients with Dox. In sum, it is still unclear whether Dox-induced long-term cardiotoxic effects in humans, in particular in children and young adults, could in part result from impaired regeneration of the heart due to cell types that are highly sensitive to Dox and that contribute to heart regeneration.

Here, we introduce human induced-pluripotent stem cells (iPSCs) and iPSC-derived cardiomyocytes (iCMs) as a cellular model system for stem cells and their differentiated progeny allowing us to dissect whether treatment of iPSCs with rather mild doses of Dox has any consequences in iPSCs and their progeny. We show that iPSCs are highly sensitive to mild and transient Dox treatment regimens. These treatment schemes are insufficient to induce either a significant DNA damage response or to impair cardiomyocyte differentiation. However, they are accompanied by substantial and persistent impairments of mitochondrial function in iPSCs. Furthermore, treatment at the iPSC stage causes major alterations in gene expression profiles as well as functional parameters, such as the beating frequency, in cardiomyocytes originating from the early-treated iPSC pool. Our data demonstrate that iPSCs undergo a process of priming that appears to not only promote a transcriptional program involved in fibrosis on one hand, and suppress the expression of genes associated with cardiac muscle function on the other hand, but also to result in major functional changes including impaired contractility, Ca^2+^ handling and altered mitochondrial functions. Overall, we propose that the administration of Dox to a pool of sensitive stem cells prior to cardiomyocytes differentiation could result in the manifestation of long-term effects impacting cellular renewal of cardiomyocytes. We suggest that this could represent an underappreciated mechanism contributing to long-term cardiotoxicity after chemotherapy particularly found in pediatric patients.

## Results

### Human iPSCs are highly sensitivity to single pulse-treatment with doxorubicin

**Fig. 1:**
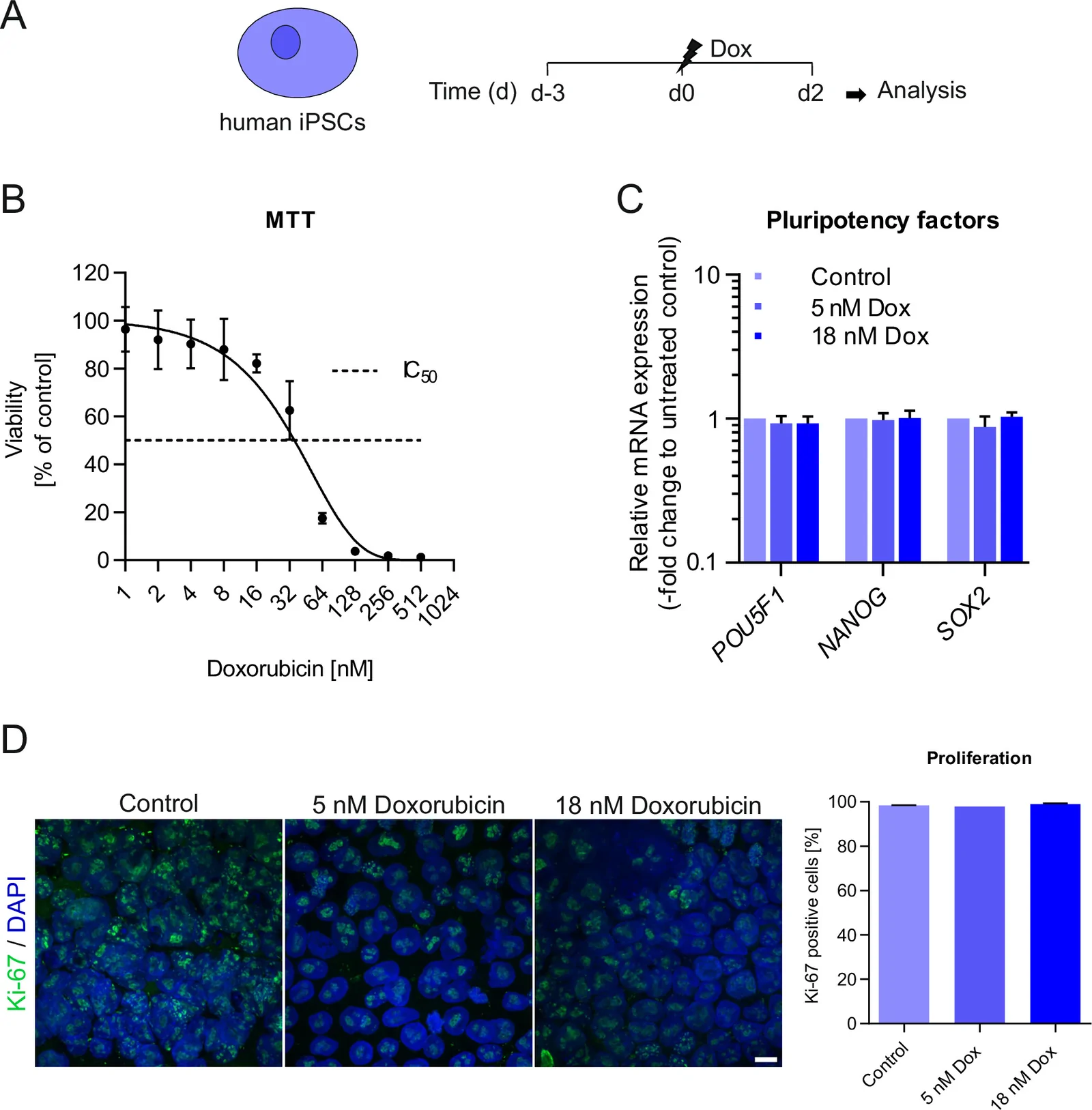
Human induced-pluripotent stem cells (iPSCs) are highly sensitive to doxorubicin pulse treatment. **A** Schematic illustration of pulse-treatment scheme with Dox in iPSCs. Pulse-treatment with Dox is represented by a lightning symbol. **B** Viability of iPSCs was elucidated 48 h after pulse-treatment with Dox using MTT assay. Data points represent the mean ± SEM of three independent experiments (*n* = 3; *N* = 8). Dashed line marks the IC_50_-value. **C** Quantitative real-time PCR results of the mRNA expression of stem cell markers POU Class 5 Homeobox 1 (*POU5F1*), Nanog Homeobox (*NANOG*) and SRY-Box Transcription Factor 2 (*SOX2*). Relative mRNA expression of Dox-treated iPSC compared to the control represent the mean ± SEM (*n* = 5; *N *= 3). **D** Immunohistochemical detection of the proliferation marker Ki-67 (green color) in Dox-treated and untreated iPSC. DNA was counterstained with DAPI (blue color). Left side, representative images. Magnification: 63x, scale bar 10 µm. Right side, quantitative results of two independent experiments of Ki-67 positive cells in iPSC 48 h after Dox treatment. Data represent the average percentage ± SEM of two independent experiments (*n* = 2; *N* = 100 nuclei).

Plasma concentrations of Dox in cancer patients receiving fast infusions have been observed to decline rapidly reaching low steady-state concentrations between 25 and 250 nM^15^. Previously, we established an in vitro treatment scheme aiming to mimic the clinical situation in patients using a single pulse-treatment with Dox comprising 2 h treatment (pulse) with concentrations ranging from 10 to 500 nM^34^. Here, we treated iPSCs for 2 h with Dox followed by a 48 h recovery phase (Fig. 1A). To elucidate the toxicity of Dox on iPSCs under these conditions, cell viability of iPSCs with increasing Dox concentrations (range 1–512 nM) was determined 48 h after Dox pulse-treatment using an MTT assay (Fig. 1B). iPSCs showed a remarkably high sensitivity to Dox (IC_50_-value = 35 nM) despite the short exposure time of 2 h. This was corroborated using another assay commonly used, namely Sulforhodamine B assay (Supplementary Fig. 1) as well as by using two other iPSCs cell lines, namely iPSC11 and iPSC12 cells (Supplementary Fig. 12A, B). Due to the high sensitivity of iPSCs and the rationale to ideally induce stress only moderately, we used concentrations within the IC_10_ and IC_30_-values (5 nM and 18 nM, respectively) for all subsequent experiments. In this way, only mild stress was applied, furthermore avoiding concentrations of Dox that would prevent cardiac differentiation. To ascertain whether iPSCs maintain their pluripotency when exposed to moderate doses of Dox, expression levels of established pluripotency markers *POU5F1*, *NANOG* and *SOX2* were determined. No apparent differences in the expression of pluripotency markers were detectable (Fig. 1C) demonstrating that the pluripotent state of iPSCs was not affected upon pulse-treatment with Dox. To analyze whether the proliferation capacity of iPSCs was maintained upon Dox treatment, the extent of Ki-67, a well-known proliferation marker, detectable within the cell nucleus was determined via immunofluorescence staining. Dox treatment did not cause any reduction in this marker (Fig. 1D) demonstrating that proliferation of iPSCs was not significantly altered upon Dox treatment. Thus, short pulse-treatment of iPSCs with the chosen sub-lethal doses of Dox did not result in a detectable change in the expression of surrogate markers for pluripotency and proliferation of iPSCs.

### Mild pulse-treatment with doxorubicin is not sufficient to induce a major DNA damage response or apoptosis

**Fig. 2:**
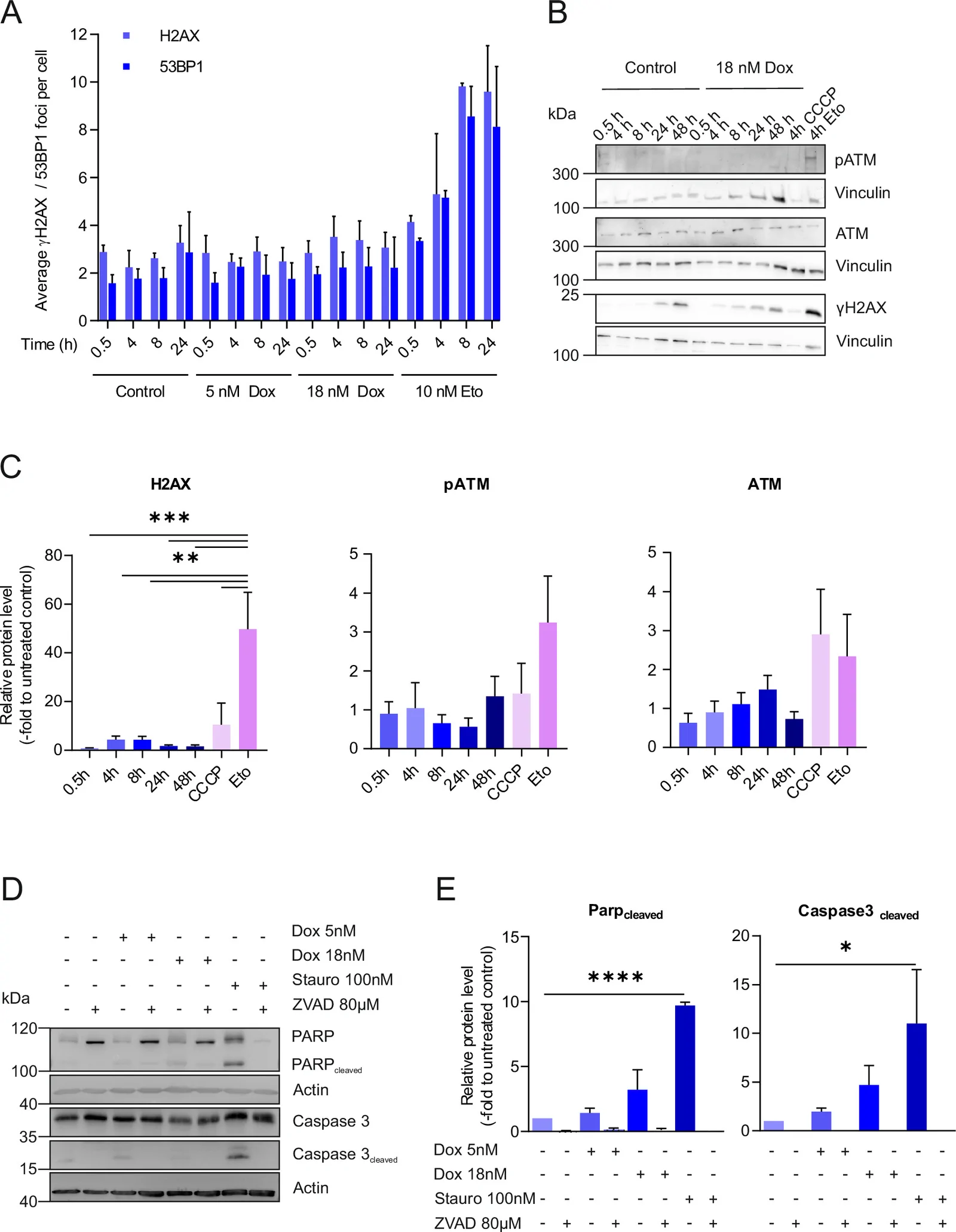
Doxorubicin pulse-treatment is not accompanied by DNA damage response or apoptosis in human iPSCs. **A** Quantitative analysis of immunohistochemical detection of the DNA damage marker γH2AX and 53BP1 0.5, 4, 8, and 24 h after 2 h Dox pulse-treatment. Data points shown are the mean ± SEM (*n* = 3; *N* = 30–100 nuclei). As a positive control, iPSCs were treated with 10 nM Etoposide (Eto) for 2 h. **B** Representative cropped immunoblots of DNA damage markers γH2AX, ATM, and its activated phosphorylated form pATM (Ser1981). Treatment conditions and time points of analysis are indicated. 10 µM Carbonylcyanid-m-chlorphenylhydrazon (CCCP) and 10 nM etoposide (Eto), each for 4 h, served as positive controls for mitochondrial and genomic stress, respectively. **C** Quantification of relative protein expression levels of DNA damage marker proteins in Dox-treated iPSCs compared to their respective time point control. For CCCP and Eto, relative expression levels were normalized to the 4 h control time point. Shown are relative protein expression levels ± SEM (*n* = 3). Vinculin was used as loading control. Full-length gels/blots of (**B**) are presented in Supplementary Fig. 15A. **D** Representative cropped western blots of apoptosis markers PARP (total and cleaved PARP), Caspase 3, and Caspase 3-cleaved products determined 48 h after treatment. Full-length gels/blots of (**D**) are presented in Supplementary Fig. 15B**. E** Relative protein levels of untreated iPSC were set to 1.0. Actin was used as loading control. Quantitative data represent the mean ± SEM (*n* = 3). Quantification was performed with ImageJ software. For statistical analysis, One_-_way ANOVA with multiple comparisons was performed. *p-value ≤ 0.05, **p-value ≤ 0.01, ***p-value ≤ 0.001, ****p-value ≤ 0.0001. Full-length blots of (**B** and **D**) are presented in Supplementary Fig. 15.

The intricate relation between DNA damage repair (DDR) and apoptosis induction upon genotoxic stress is well known^35^. For stem cells exposed to genotoxic substances, DDR and regulation of apoptosis are essential for survival and stem cell pool maintenance. To analyze whether the mild and short Dox treatment conditions result in the induction of a DDR, several markers of this pathway were determined over time. First, the DDR marker proteins Ser139-phosphorylated histone variant H2AX (γH2AX) and tumor suppressor p53-binding protein 1 (53BP1) were quantified for the first 24 h using immunofluorescence staining. Etoposide (Eto) was used as a positive control for the induction of DNA double-strand breaks (DSBs). Compared to the control, neither of these two markers exhibited a discernible increase overtime following Dox treatment. In contrast, the administration of Eto resulted in a significant increase (Fig. 2A). To further validate that DDR is not activated upon transient exposures of iPSCs to moderate doses of Dox, protein expression levels of γH2AX, protein kinase ataxia telangiectasia mutated (ATM), and its activated phosphorylated form pATM were determined over the same time-course using immunoblotting. Also here, neither of these markers showed a Dox-dependent increase when compared to the control (Fig. 2B, C). We conclude that Dox, as applied here, did not induce DSBs to a major extent, suggesting that the observed high sensitivity of iPSCs towards Dox is possibly not caused by excessive induction of DSBs in the nucleus.

To elucidate whether Dox exposure induces apoptosis in iPSCs, protein levels of established markers involved in the apoptotic cascade were determined using immunoblotting. Short pulse-treatment of iPSCs with Dox was not sufficient to induce PARP cleavage and Caspase 3 activation in a significant manner, only a moderate non-significant increase was observed using 18 nM Dox (Fig. 2D, E). As expected, treatment with the positive control staurosporine caused a significant induction of apoptosis. Consistent with our results on DDR, we conclude that short pulse-treatment of iPSCs with moderate doses of Dox does neither induce DDR nor apoptosis to a major extent. These findings distinguish this treatment scheme from others in the literature using much higher Dox concentrations and/or incubation times.

### Doxorubicin-induced mitochondrial fragmentation in iPSCs is accompanied with loss of mitochondrial membrane potential

**Fig. 3:**
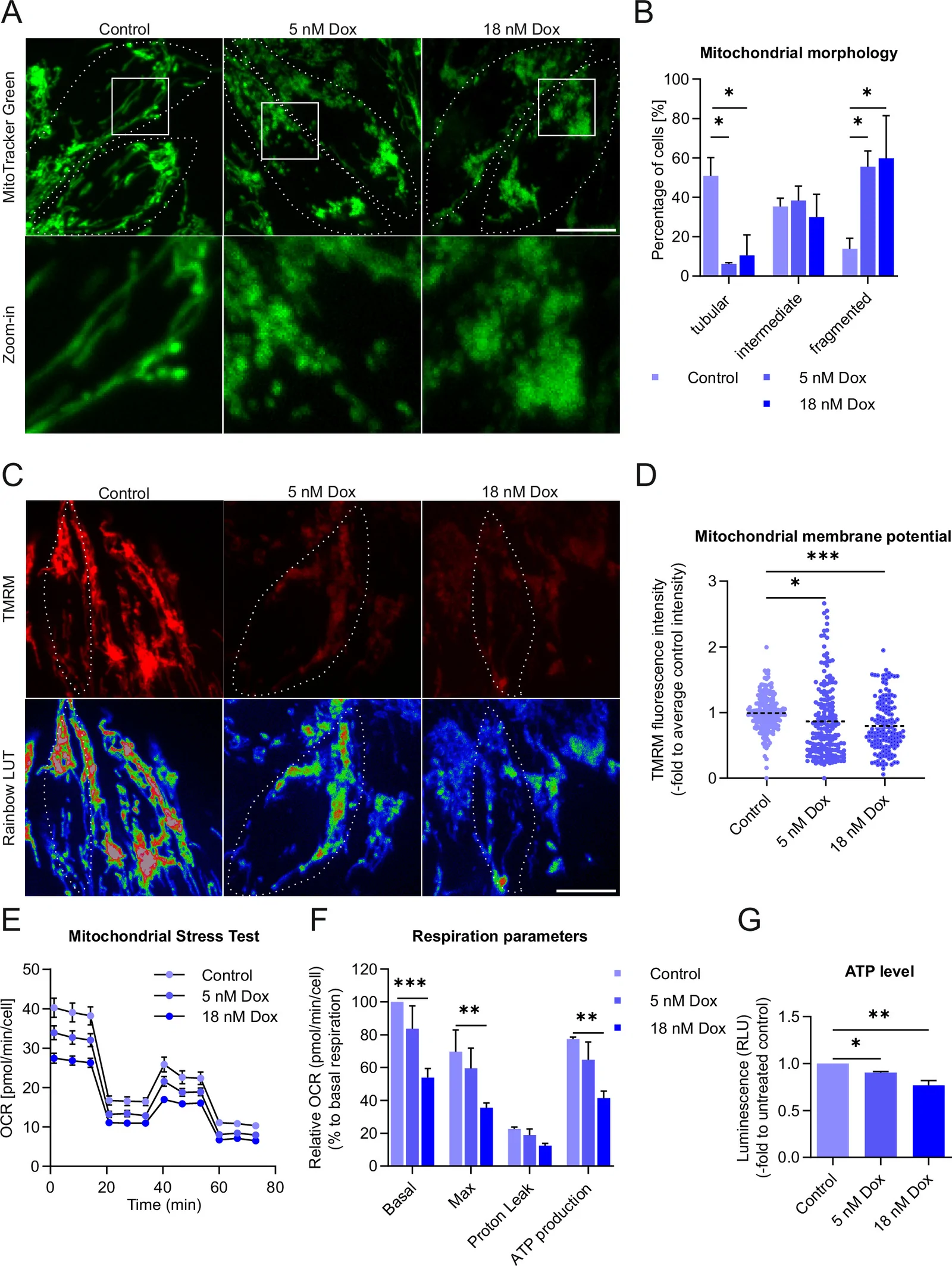
Doxorubicin pulse treatment of iPSCs impairs mitochondrial morphology and mitochondrial bioenergetics at multiple levels. **A** Representative confocal image of MitoTracker™Green FM-stained mitochondria of control and pulse-treated iPSCs. Magnification: x63, scale bar: 10 μm. Cell surroundings are marked with dashed lines. **B** Quantification of mitochondrial morphology categorized in tubular, intermediate, or fragmented 48 h after Dox treatment. Data show mean ± SEM (*n* = 3; *N* = 50 cells). 2-way ANOVA was performed. *p-value ≤ 0.05. **C** Representative confocal image of TMRM-stained iPSCs (upper row). Intensity is shown in pseudo-color rainbow LUT from confocal images (lower row). Magnification: ×63, scale bar: 10 µm. **D** Quantification of relative TMRM fluorescence intensity in Dox-treated iPSCs relative to the untreated control. Data represent the median plus whiskers (*n* = 3; *N* = 35–50 cells). For statistical analysis, one-way ANOVA with multiple comparisons was performed. *p-value ≤ 0.05. ***p-value ≤ 0.001. **E** Representative Seahorse run of one biological replicate. Shown are the oxygen consumption rates (pmol O_2_/min) normalized to the cell number (by Hoechst staining). **F** Relative basal respiration, maximal respiration (after FCCP injection), proton leak and ATP production are shown for iPSCs after 48 h of 2 h pulse-treatment with Dox. Data were normalized to the untreated control and represents the mean ± SEM (*n *= 3). For statistical analysis, *2-way* ANOVA with multiple comparisons was performed. **p-value ≤ 0.01, ***p-value ≤ 0.001. **G** Quantification of relative ATP levels 48 h after 2 h pulse-treatment with Dox using CellTiter-Glo® 2.0 Cell viability assay. Data represent the mean ± SEM (n = 3; *N* = 4), normalized to the respective control. *One-way* ANOVA with multiple comparisons was performed. *p-value ≤ 0.05, **p-value ≤ 0.01.

In order to check whether short pulse-treatment of iPSCs with low doses of Dox is accompanied with changes in mitochondrial function, mitochondrial morphology was studied using live-cell confocal microscopy in iPSCs and mitochondrial morphology was categorized into one of the following three categories: “tubular”, “intermediate” or “fragmented”. Overall, the average percentage of fragmented mitochondria was significantly elevated already at the lowest concentration of Dox of 5 nM (Fig. 3A, B). Consistent with this, we observed a moderate, yet significant, increase of OPA1 processing (Supplementary Fig. 2F,G) with 18 nM Dox indicating an impairment of mitochondrial functionality even upon short pulse-exposures with moderate doses of Dox. To elucidate whether Dox pulse-treatment is sufficient to induce an imbalance in mitochondrial membrane potential (ΔΨ) in iPSCs, we measured the relative intensity of TMRM within mitochondria using live-cell confocal microscopy. Here, even after a recovery period of 48 h Dox pulse-treatment was sufficient to significantly reduce Δ*Ψ* of mitochondria in iPSCs in a persistent manner (Fig. 3C, D). This appeared to be slightly more pronounced at 18 nM Dox compared to 5 nM Dox. In sum, pulse-treatment of iPSCs with moderate doses of Dox is sufficient to impair mitochondrial function, and that this impairment is not reversed within the recovery period of 48 h. As the same conditions do not appear to induce significant alterations in nuclear DDR or apoptosis, we conclude that these drug-treatment conditions may selectively impair mitochondrial functionality. We propose that with these findings it is now possible to dissect known mitochondrial from nuclear effects, supporting the view that mitochondria are a critical target of Dox.

### Pulse-treatment of iPSCs with doxorubicin impairs mitochondrial respiration and ATP formation

Since loss of mitochondrial membrane potential could result from impaired mitochondrial respiration, live cell respirometry was performed using a Seahorse Flux Analyzer. We observed a significant reduction in the overall oxygen consumption rate (OCR), maximal respiration (Max) and ATP production upon treatment with Dox (Fig. 3E, F). To further validate the Dox-mediated decrease in ATP production, steady-state levels of ATP were measured with the CellTiter-Glo® assay. With increasing Dox concentrations, ATP levels significantly decreased reaching a reduction of about 20% when 18 nM Dox was applied (Fig. 3G). Corroborating to our data, short pulse-treatment with Dox also led to reduced ATP levels in iPSC11 and iPSC12 with enhanced fragmentation of mitochondria in iPSC11. These findings emphasize that Dox-induced mitochondrial dysfunction, manifesting at low doses, is not confined to a particular iPSC cell line (Supplementary Fig. 12C-H). We next determined mitochondria ROS formation, yet did not observe that this was altered by applying moderate doses of Dox (Supplementary Fig. 2A). Furthermore, we asked whether the suppression of mitochondrial respiration was linked to changes in mitochondrial ultrastructure. For this, transmission electron microscopy was performed and crista were categorized into four main morphologies: Tubes, septa, arches and branches. No significant changes in the cristae number per mitochondria after pulse-treatment with Dox were observable (Supplementary Fig. 2B,C). Next, the influence of low Dox doses on the assembly of oxidative phosphorylation (OXPHOS) complexes was determined via performance of BN- and CN-PAGE analyses. Short pulse-treatment of iPSCs with Dox had no significant effect on the steady-state assembly of different OXPHOS complexes or the MICOS complex in iPSCs (Supplementary Fig. 2D,E). However, it is noteworthy that iPSCs clearly harbor supercomplexes, high-molecular-weight complexes composed of at least two different OXPHOS complexes, an observation that, to our knowledge, was not reported before. Measurement of in-gel activity of complex I and complex IV showed no significant effect of Dox on their activities. We further analyzed protein levels of numerous mitochondrial proteins after short pulse-treatment with Dox. Most proteins did not show any major differences, still the levels of some mitochondrial ribosomal subunits such as MRPS25 and MRLP3 appeared slightly increased (Supplementary Fig. 3). Overall, pulse-treatment showed a clear reduction in ATP levels and in mitochondrial respiration, however, this was apparently neither a result of altered ROS levels, mitochondrial ultrastructure, mitochondrial OXPHOS protein levels, nor due to defects in OXPHOS complex assembly.

### Pulse-treatment with doxorubicin modulates organization of the mitochondrial genome in iPSCs

**Fig. 4:**
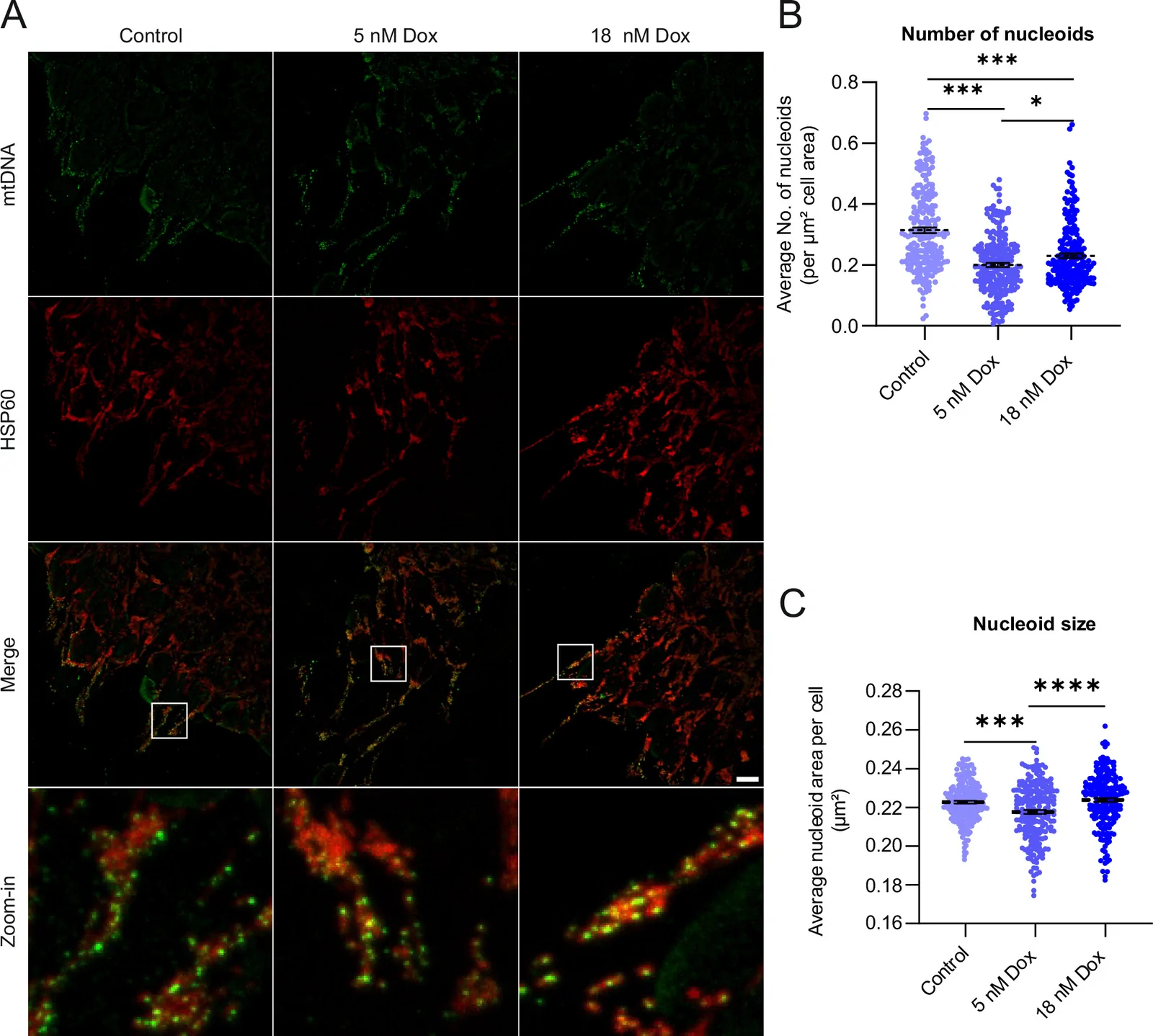
Pulse-treatment of iPSCs with doxorubicin alters mtDNA organization. **A** Representative confocal image of mitochondrial nucleoids (α-DNA) in iPSC upon 5 nM and 18 nM Dox treatment. Magnification: ×63, scale bar: 10 μM. Zoomed regions are marked with a white square. Quantification of the average amount of mitochondrial nucleoids per µm² per cell (**B**) and nucleoid size in µm² (**C**) after pulse-treatment with Dox. Data show mean ± SEM (*n* = 3; *N* = 50 cells). *One-way* ANOVA was performed. *p-value ≤ 0.05,**p-value ≤ 0.01, ***p-value ≤ 0.0001.

Within mitochondria, several hundreds to thousands of mtDNA copies are organized as punctate-like nucleoid structures harboring ~1.4 mtDNA molecules per nucleoid on average^36^. To determine the effect of Dox on mitochondrial nucleoid organization, mitochondrial nucleoid content and morphology were determined using confocal microscopy. Upon Dox treatment, the average number of mitochondrial nucleoids per cell was reduced with both concentrations of Dox compared to the control, even showing a slightly more pronounced effect when using 5 nM Dox (Fig. 4A, B). Next, we determined the average size of mitochondrial nucleoids. Pulse-treatment of iPSCs is accompanied with a moderate but significant reduction in nucleoid size of nucleoid size when 5 nM Dox was applied compared to the untreated control as well as to the 18 nM Dox-treated condition (Fig. 4A, C). At 18 nM Dox there was no significant change of the average size compared to the control. To elucidate whether Dox-dependent modulation of mitochondrial nucleoids results in overall reduction in mitochondrial DNA content (mtDNA copy number) was measured by quantitative real-time PCR (Supplementary Fig. 2H). Upon treatment of iPSCs with Dox, only a moderate, yet not significant, increase in mtDNA copy number was found. Also, the protein levels of mtDNA-associated proteins TFAM and Twinkle were not significantly altered (Supplementary Fig. 3). In summary, the administration of pulse-treatment with Dox in human iPSCs has been demonstrated to induce a modulation in the organization of mitochondrial DNA, characterized by a moderate change in the size of mitochondrial nucleoids, concurrent with a decrease in nucleoid number. This finding is consistent with the possibility that mitochondrial nucleoids may actually undergo remodeling in response to Dox-dependent inhibition of mtDNA replication.

### Pulse-treatment of human iPSCs with doxorubicin results in major changes in gene expression modulating cellular homeostasis and metabolism

**Fig. 5:**
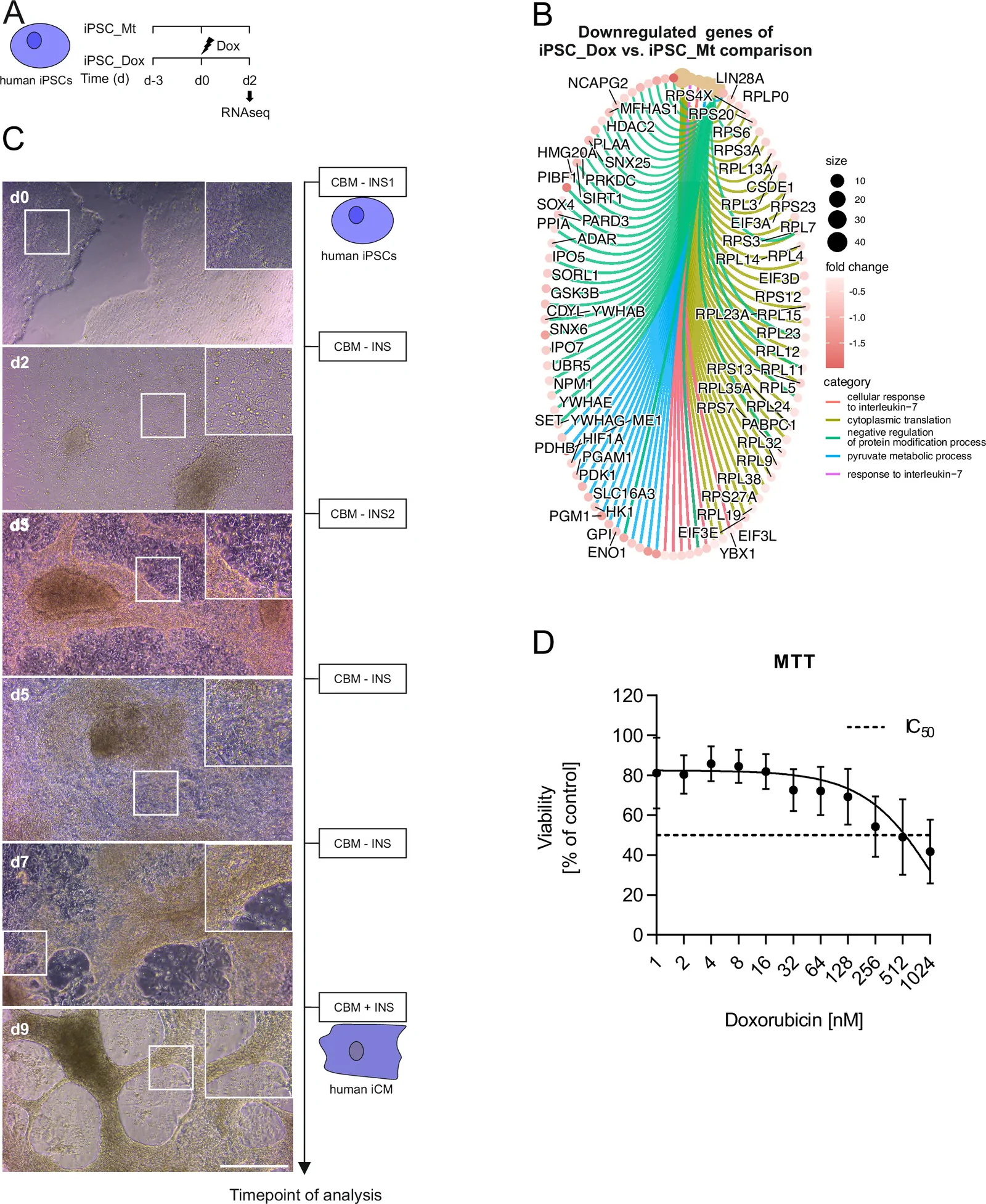
Dox alters gene expression modulating cellular homeostasis and metabolism in human iPSCs and iCMs, showing increased resistance against doxorubicin. **A** Schematic illustration of the treatment scheme in iPSCs prior to short-read-NGS RNAseq analysis at day 2 (d2). Time point of 2 h pulse-treatment with 18 nM doxorubicin (Dox) is indicated by a lightning symbol. **B** Gene concept network of GO-term enrichments and associated downregulated genes of pairwise comparison between Dox-treated iPSCs and the respective control. Number of genes, logFC-value, and GO-terms are listed in the respective legend. **C** Scheme of iPSC differentiation into iPSC-derived cardiomyocytes (iCMs) following a chemically defined protocol. At defined days (d), cardiac basal media (CBM) was supplemented without B-27 minus insulin (-INS) or with insulin (+INS). In addition, growth factors such as CHIR99021 (CBM-INS1) or IWP4 (CBM-INS2) were added at specific days of the differentiation protocol. Days with negative numbers represent the time of culturing iPSCs until initiation of cardiac differentiation. On day 7, differentiation of iPSCs into iPSC-derived cardiomyocytes (iCMs) is completed. Representative images of iCMs acquired with Leica DM IL LED Fluo Cellfactory (Leica). Days (d) of cardiac differentiation are indicated. Magnification: 20×, scale bar: 300 µm. **D** Determination of the sensitivity of iCMs against Dox. iCMs were pulse-treated for 2 h with increasing doses of doxorubicin (Dox) and viability was determined using an MTT assay. The mean ± SEM of three independent biological replicates (*n* = 3; *N* = 4–8) is shown.

Since we observed that pulse-treatment of iPSCs with Dox impairs mitochondrial functions, morphology and mtDNA organization, we asked which transcriptional response on a genome-wide level is induced by this treatment and whether metabolic pathways linked to mitochondria are affected. For this, we performed whole genome transcriptome profiling of human iPSCs 48 h after 2 h pulse-treatment with Dox and the respective mock-treated iPSC control (Fig. 5A). Multidimensional scaling revealed high homogeneity within the respective replicates (Supplementary Fig. 5). In total, expression levels of 16198 genes were obtained from the short read-NGS RNAseq dataset. Pairwise comparison between pulse-treated iPSCs (iPSC_Dox) and the respective control (iPSC_Mt) resulted in significant upregulation of 753 and downregulation of 519 genes (Supplementary Table 3). A GO-term enrichment analysis revealed no significant functions in the genes upregulated after Dox-treatment of iPSCs, whereas, for the significantly downregulated genes an enrichment of various biological processes (BP) was seen e.g., cellular response to interleukin-7, cytoplasmic translation, negative regulation of protein modification, and pyruvate metabolic process (Fig. 5B, Supplementary Fig. 9A). Several ribosomal genes and eukaryotic initiation factors involved in cytoplasmic translation were significantly downregulated as well as Lin-28 Homolog A (*LIN28A*), a pivotal regulator of genes enhancing pluripotency in stem cells and modulating metabolism^37,38^. Additionally, genes involved in post-translational regulation, including the Ubiquitin Protein Ligase E3 Component N-Recognin 5 (*UBR5*) and Sirtuin 1 (*Sirt1*), were found to be downregulated. In addition, pulse-treatment of iPSCs was accompanied by suppression of SRY-Box Transcription Factor 4 (*SOX4*), a crucial transcription factor during embryogenesis (Fig. 5B). This suggests that even transient Dox exposure may disrupt cellular protein homeostasis and cell fate decisions. Importantly, within the group of downregulated genes, we also observed numerous factors controlling major metabolic pathways such as hexokinase 1 (*HK1*), pyruvate dehydrogenase kinase 1 (*PDK1*), phosphoglucomutase 1 (*PGM1*), pyruvate dehydrogenase E1 subunit beta (*PDHB*), and hypoxia inducible factor 1 subunit alpha (*HIF1A*). These enzymes and factors clearly hint towards an alteration of glycolysis as well as downstream metabolic pathways occurring inside mitochondria upon Dox-treatment. In sum, pulse-treatment for only 2 h with moderate doses of Dox is sufficient to cause major transcriptional changes accompanied with downregulation of genes regulating cellular homeostasis and energy metabolism in iPSCs.

### iPSCs-derived cardiomyocytes (iCMs) show a strongly increased resistance against doxorubicin compared to iPSCs

Based on the observed Dox sensitivity of iPSCs, we next asked whether iPSCs can be differentiated into iPSC-derived cardiomyocytes (iCMs) and whether the sensitivity of iCMs towards Dox differs from their undifferentiated counterparts. For this, a previously established differentiation protocol was applied (Fig. 5C) and the generated iCMs were characterized. Consistent with a successful cardiac differentiation, we observed a cell morphology resembling cardiomyocytes at day 9 of the differentiation protocol (Fig. 5C). Generated iCMs further showed spontaneous contractions starting from day 7 of the cardiac differentiation protocol (Supplementary movies 1–4). Cardiac differentiation was corroborated by quantitative real-time PCR showing enhanced expression of cardiomyocyte markers Troponin T2 (*TNNT2*), NK2 Homeobox 5 (*NKX2-5*), Myosin Heavy Chain 6 (*Myh6*) and GATA Binding Protein 4 (*GATA4*) as well as reduced expression of pluripotency markers POU Class 5 Homeobox 1 (*POU5F1*) and Nanog Homeobox (*NANOG*) (Supplementary Fig. 4A). To determine the sensitivity of iCMs against doxorubicin in a comparable manner, we applied a similar 2-h pulse-treatment scheme as in iPSCs and measured cell viability after 48 h using the MTT assay. Here, iCMs showed an IC_50-_value of ~512 nM (Fig. 5D), which is much higher than the IC_50_-value of ~35 nM for iPSCs. In sum, iCMs are considerably more resistant against Dox compared to their undifferentiated iPSCs counterparts.

### Pulse-treatment with Dox in iPSCs impairs mtDNA nucleoid organization and ATP formation in iCMs

**Fig. 6:**
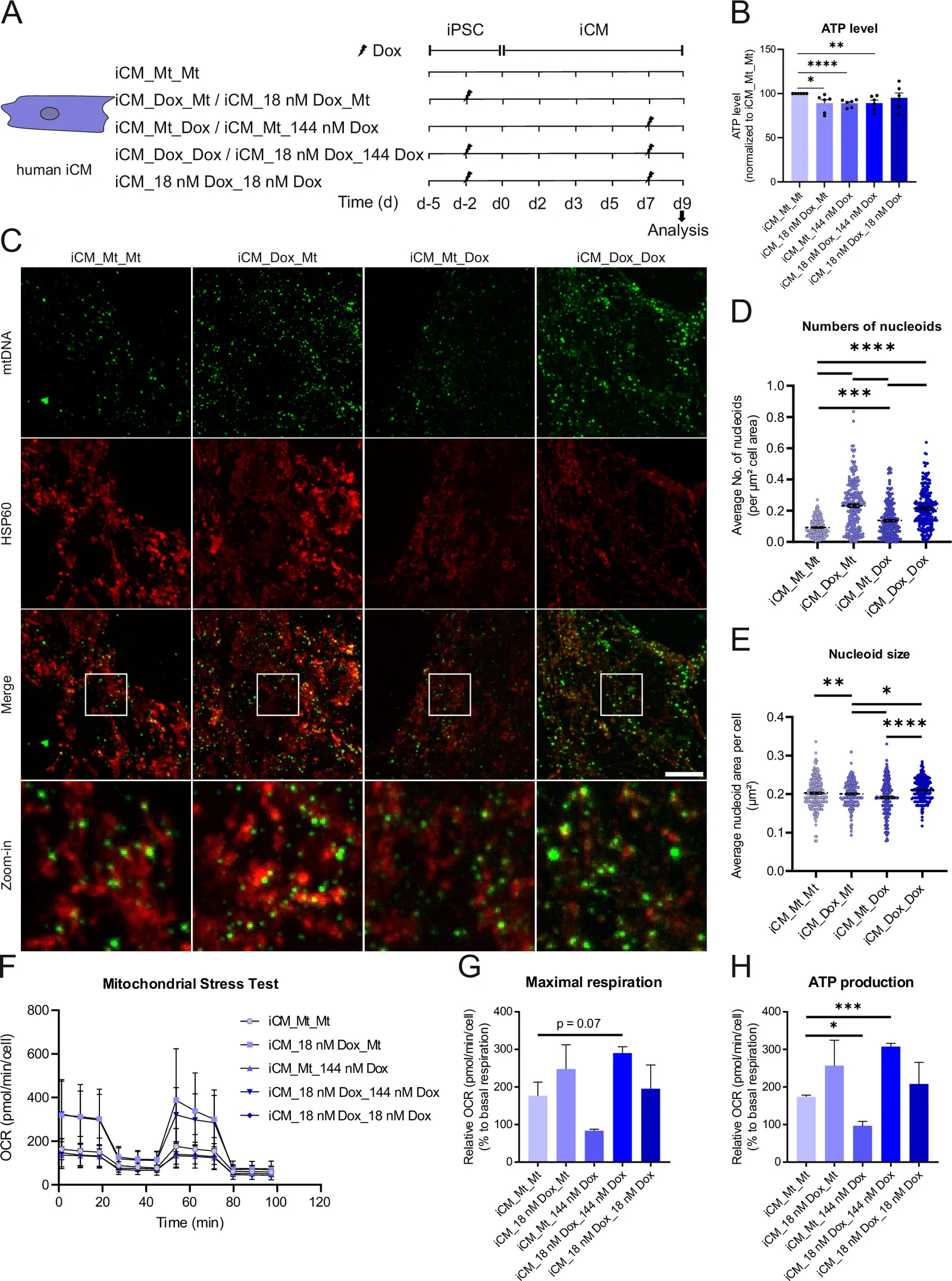
Mitochondrial mtDNA organization and bioenergetics are impaired in iCMs treated with Dox during cardiac differentiation. **A** Overview of treatment schemes in iCMs prior to whole genome-RNAseq analysis. Following samples were measured: Untreated iCMs (iCM_Mt_Mt), in iPSC stage treated iCMs (iCM_Dox_Mt), iCM treated on day 7 (iCM_Mt_Dox) and iCMs treated in iPSC stage and on day 7 of cardiac differentiation (iCM_Dox_Dox). Time point of pulse treatment with doxorubicin (Dox) is marked with a lightning symbol. **B** Quantification of relative ATP levels in iCMs pulse-treated with Dox at different stages of cardiac differentiation using CellTiter-Glo® 2.0 Cell Viability Assay. Data represent the mean ± SEM (*n* = 6; *N* = 3), normalized to respective control. Unpaired Student’s *t* test was performed. *p-value ≤ 0.05, **p-value ≤ 0.01, ****p-value ≤ 0.0001. **C** Representative confocal image of mitochondrial nucleoids (α-DNA) in iCMs (iCM_Mt_Mt), iCMs treated in iPSC stage (iCM_Dox_Mt), iCMs treated on day 7 of cardiac differentiation (iCM_Mt_Dox) and in double-treated iCMs (iCM_Dox_Dox). Magnification: ×63, scale bar: 10 μM. Zoomed-in regions are marked with a white square. Quantification of the average amount of mitochondrial nucleoids per µm² per cell (**D**) and area (µm², **E**) after treatment with Dox. Data show mean ± SEM (*n* = 3; *N* = 50 cells). One-way ANOVA was performed. *p-value ≤ 0.05, **P-value ≤ 0.01, ***P-value ≤ 0.0001. **F** Representative Seahorse run of one biological replicate. Shown is the oxygen consumption rate for each treatment condition (pmol O_2_/min) normalized to the respective cell number (by Hoechst staining). Relative maximal respiration (**G**) and ATP production (**H**) are shown for iCMs receiving Dox as pulse-treatment at different stages of cardiac differentiation. Data was normalized to basal respiration of the untreated control- Data shown represent the mean ± SEM of three independent experiments (*n* = 3; *N* = 2-4).

Next, we asked whether pulse-treatment of iPSCs with moderate doses of doxorubicin has persistent functional consequences after cells have differentiated into cardiomyocytes. In other words, are iCMs that were treated earlier at the iPSC stage primed by this? Moreover, could such pretreatment impose an advantage or disadvantage compared to mock-treated cells when coping with similar types of stress after differentiation? To address this, the following different treatment schemes were applied and iCMs were first analyzed (Fig. 6A) for their influence on differentiation and for functionality. Later, whole-genome transcriptome profiling was performed. When no concentration for Dox is indicated, the corresponding IC_30_-values for iPSCs (18 nM) or iCMs (144 nM) were used, respectively. To also maintain exposures to equal Dox doses across different differentiation states, we applied in some experiments 18 nM Dox (indicated as iCM_18 nM Dox_18 nM Dox) as using a higher dose was not tolerated by iPSCs.

First, iPSCs were either pulse-treated with the corresponding IC_30_-value (18 nM) before initiation of cardiac differentiation and analyzed at the iCM stage (iCM_Dox_Mt or iCM_18 nM Dox_Mt) or were not treated with Dox at the iPSC stage for comparison (iCM_Mt_Mt). To determine the influence of moderate pulse-treatment of Dox on terminally differentiated cardiomyocytes, iCMs were treated with 144 nM for 2 h on day 7 of cardiac differentiation followed by 48 h of recovery (iCM_Mt_Dox or iCM_Mt_144 nM Dox). To investigate whether early pulse-treatment of iPSCs alters the response towards Dox treatment at a later stage of cardiac differentiation, Dox-treated iPSCs were exposed to a second dose of Dox at the iCM stage (iCM_Dox_Dox or iCM_18 nM_Dox_144 nM).

We noted that differentiation into iCMs was not impaired independent of the time point of Dox pulse-treatment as cellular morphology and beating of iCMs was observed (Supplementary Movies 1–4). Also, the observed expression levels of cardiac differentiation and pluripotency markers by quantitative real-time PCR corroborate this finding (Supplementary Fig. 4A). Next, we addressed the functional consequences regarding mitochondrial morphology as well as mitochondria-linked cellular respiration using a Seahorse Flux Analyzer. Neither mitochondrial morphology, basal respiration, maximal respiration, nor spare respiratory capacity were grossly affected in any of the treatment conditions when compared to the untreated control (Supplementary Fig. 4C-F, Fig. 6F, G). We noted a moderate trend showing mitochondrial elongation and enhanced mitochondrial maximal respiration when cells have experienced priming (iCM_18 nM Dox_Mt, iCM_18 nM Dox_144 nM Dox) and an opposing trend of upon late Dox treatment (iCM_Mt_144 nM Dox). Also, ATP production was significantly reduced in the latter condition (Fig. 6H). Interestingly, when iCMs were primed prior to receiving the second dose of Dox, we could not observe this drop in ATP production, but rather the opposite (Fig. 6H). Taken together, these findings are in line with a putative hormesis effect of priming at an iPSC stage, as iCMs appear partially protected against Dox-induced loss in ATP production, mitochondrial fragmentation, and impaired oxidative phosphorylation when pretreated at the iPSC stage.

Total ATP levels were significantly reduced in iCMs that were primed either only once with Dox (iCM_18 nM Dox_Mt) or when applied a second time at the iCM stage (iCM_18 nM Dox_144 nM Dox; Fig. 6B). Late treatment alone (iCM_Mt_144 nM Dox) also led to significantly reduced ATP levels (Fig. 6B).

Given that iPSCs showed altered mtDNA organization upon Dox treatment (Fig. 4), we next asked whether iCMs show a Dox-dependent alteration of mtDNA. Indeed, the number of nucleoids was increased in all tested treatment regimens (Fig. 6C, D) compared to the control. Moreover, the nucleoid size was significantly reduced in iCMs that were primed at the iPSC stage (iCM_Dox_Mt; (Fig. 6C, E). In sum, we conclude that priming at an iPSC stage is persistent and has functional consequences on mitochondrial functions, including mtDNA organization, much later at the iCM stage.

### The transcriptional response to pulse-treatment with doxorubicin reveals major differences regarding energy metabolism between iPSCs and iCMs

**Fig. 7:**
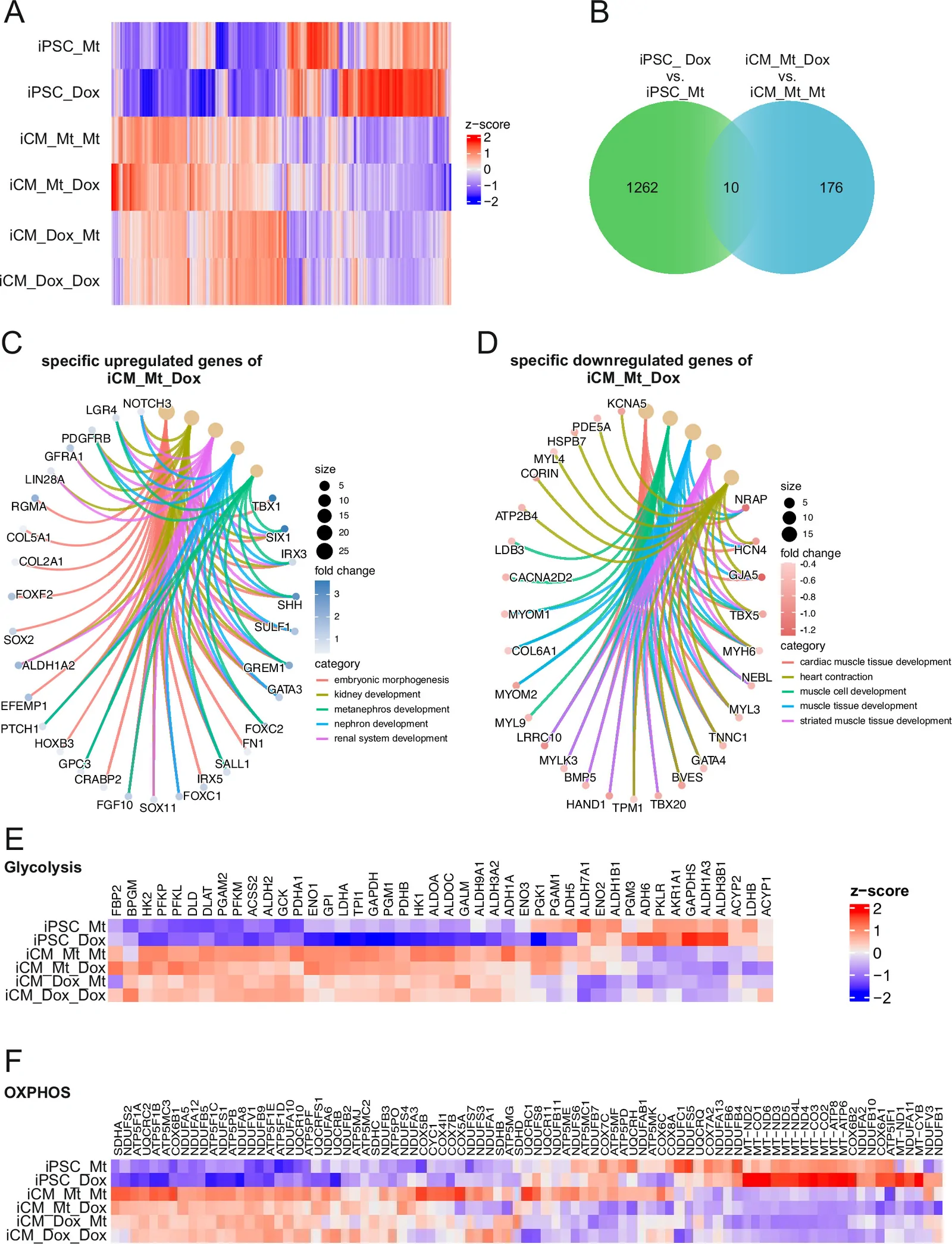
Whole-genome transcriptome analysis reveals differences in the response of iPSCs and iCMs towards doxorubicin treatment. **A** Heatmap of all 16198 genes detected in the indicated six treatment conditions. The average z-score is given for all four technical replicates in all conditions scaled per gene. **B** Venn diagram of pairwise comparisons between Dox-treated iPSCs and iCMs pulse-treated with Dox on day 7 of cardiac differentiation compared to the respective control. Gene concept network of GO-term enriched upregulated (**C**) and downregulated (**D**) genes of pairwise comparison between Dox-treated iPSCs (iPSC_Dox) and iCMs (iCM_Mt_Dox). The number of genes, logFC-value, and GO-terms are indicated. Heatmap of targeted analysis of genes involved in glycolysis and gluconeogenesis (**E**) and oxidative phosphorylation (OXPHOS, **F**). Average z-score for all four technical replicates of each condition is shown. Coloring for z-scores as indicated.

To test whether different Dox treatment regimens change gene expression we performed whole genome transcriptome profiling using the indicated conditions (Fig. 7A). The transcriptome analysis revealed numerous significantly differentially expressed genes (Supplementary Table 3 and Supplementary Data) and multidimensional scaling of the data showed a clear trend of separation between the six groups with a high homogeneity within the replicates (Supplementary Fig. 5). Overall, data for 16,198 genes were received from the RNAseq data set. By performing hierarchical clustering, we found strong differences in gene clusters between iPSCs and their differentiated counterparts reflecting the major transcriptional changes induced by the differentiation process itself (Fig. 7A; Supplementary Fig. 8). Thus, independent of the time point of Dox treatment, the expression of typical cardiac differentiation markers was also seen on a whole transcriptome level confirming that differentiation of iPSCs into cardiomyocytes proceeds despite prior or concurrent Dox exposure.

Given that both untreated and Dox-treated iPSCs can differentiate into iCMs, we next asked how iCMs are affected by a pulse-treatment with doxorubicin on a global transcriptome level and whether the response is different to the Dox effect in iPSCs described earlier. For this, pairwise comparisons between Dox-treated iPSCs and iCMs on day 7 of cardiac differentiation to their respective control were performed (iPSC_Dox vs. iPSC_Mt; iCM_Mt_Dox vs. iCM_Mt_Mt) to determine significantly differentially regulated genes upon Dox treatment (Fig. 7B; Supplementary Table 3 and Supplementary Data). A subsequent pairwise comparison between the resulting gene sets revealed a total of 842 significantly upregulated and 606 downregulated genes (Fig. 7B). Not surprisingly, most changes were specific to the cell type treated, whereas only 10 significantly differentially regulated genes were shared between both conditions. Importantly, iPSCs showed a higher number of specific genes (1262) significantly affected by moderate pulse-treatment with Dox as compared to their differentiated counterparts (176), supporting the hypothesis that Dox has a much stronger impact on gene expression in iPSCs compared to iCMs. GO-term enrichment of biological processes (BP) analysis of genes solely affected in iPSCs (1262) resulted in the enrichment of the same GO-terms as described previously for Dox-treated iPSCs (Fig. 5B). In contrast to iPSCs, the number of specific and significantly affected genes in iCMs was much lower, with 89 upregulated genes and 87 downregulated genes. An analysis of those significantly upregulated genes in Dox-treated iCMs revealed GO-term enrichment of biological processes (BP) linked to embryonic morphogenesis and kidney development (Fig. 7C, Supplementary Fig. 9C). Among these GO-term functional enrichments associated genes such as T-Box transcription factor 1 (*TBX1*), SIX homeobox 1 (*SIX1*), sonic hedgehog signaling molecule (*SHH*), and repulsive guidance molecule BMP co-receptor A (*RGMA*) have been shown to be highly upregulated indicating Dox-induced upregulation of processes associated with embryogenesis. As shown by GO-term enrichment analysis, pulse-treatment with Dox induces the repression of genes associated with cardiac muscle development, heart contraction, muscle cell development, muscle tissue development, and striated muscle tissue development (Fig. 7D, Supplementary Fig. 9C). Here, gap junction protein alpha 5 (*GJA5*), nebulin-related anchoring protein (*NRAP)*; and hyperpolarization-activated cyclic nucleotide-gated potassium channel 4 (*HCN4*) were suppressed upon pulse-treatment with Dox. Suppression of these genes are linked to impaired formation of electrochemical gradients and cellular connectivity necessary for cardiac function. Taken together, iPSCs and iCMs show major differences in the transient transcriptional response upon Dox pulse treatment. Pulse-treatment of iCMs with Dox was not accompanied by major significant GO-terms associated with metabolism as seen in iPSCs, pointing to distinct effects of DOX in iCMs versus iPSCs. Since we could show that pulse-treatment of iPSCs is accompanied by downregulation of metabolic function (Fig. 3E–G), we questioned whether this effect is reflected in our transcriptomic data. A targeted analysis using a defined gene set for glycolysis and gluconeogenesis from Harmonizome 3.0^39,40^ revealed a Dox-induced reciprocal transcriptional response of essential genes of glycolysis in iPSCs versus iCMs (*HK1, GAPDH, ALDOA, ALDOC, LDHA*, *PGK1*) independent of the time point of treatment (Fig. 7E, Supplementary Fig. 8E-H). Similar reciprocal effects were observable when analyzing a defined set of genes for oxidative phosphorylation (OXPHOS) from Harmonizome3.0 (Fig. 7F, Supplementary Fig. 8I,J). It is noteworthy that iPSCs showed a very robust upregulation of all 13 mitochondrial encoded genes (*MT-ATP6, MT-ATP8, MT-CO1, MT-CO2, MT-CO3, MT-CYB, MT-ND1, MT-ND2, MT-ND3, MT-ND4, MT-ND4L, MT-ND5, MT-ND6*) which was not seen in iCMs. We also checked in publicly available databases whether OXPHOS gene expression is altered in the hearts of deceased patients that have been treated with Dox earlier^41^. The variation of the selected limited data set is quite high which likely results from a combination of inter-patient variability and the fact that heart tissue consists of multiple cell types at a highly variable proportion. Still, it is interesting to note that also here mtDNA-encoded genes are often expressed higher in patients who have undergone Dox treatment compared to healthy patients (Supplementary Fig. 14B). Future studies will have to dissect this further, in particular which cell types are responsible for this in humans. In sum, moderate single pulse-treatment of iPSCs and iCMs revealed major differences in the transcriptional response for the two differentiation states. While the transcriptional response in iCMs clearly modulated gene expression mediating cardiac function, the response of iPSCs revealed many genes altering major metabolic processes such as glycolysis and OXPHOS. In iCMs, the latter effect was not evident or appeared even reciprocal (Supplementary Fig. 8E-J). Thus, even moderate pulse-treatment in iPSCs is accompanied with upregulation of mtDNA-encoded genes supporting the hypothesis that this could represent a compensatory mechanism to balance mitochondrial dysfunction upon treatment with Dox.

### A single early pulse-treatment with Dox in iPSCs causes transcriptional adaptations in iCMs associated with impaired cardiac muscle regeneration

**Fig. 8:**
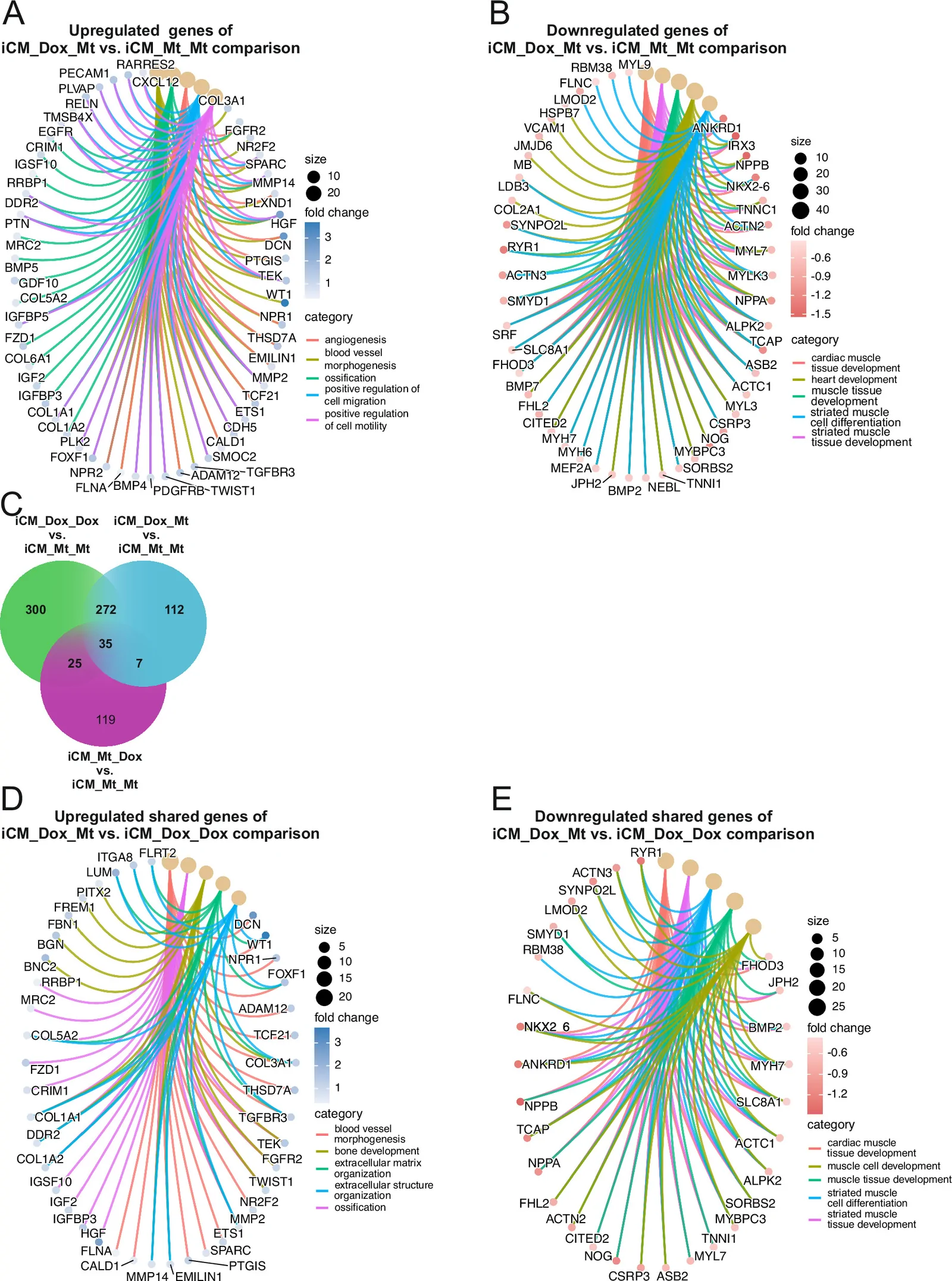
Early doxorubicin treatment of iPSCs is accompanied with whole genome transcriptional reprogramming in iCMs. Gene concept network of GO-term enrichments of differentially upregulated (**A**) and downregulated (**B**) genes of iCMs (iCM_Dox_Mt) compared to mock-treated control (iCM_Mt_Mt). GO-terms of associated genes, number of genes, and logFCs are indicated. **C** Venn diagram of significantly differentially regulated genes resulting from indicated pairwise comparisons in which mock-treated iCMs (iCM_Mt_Mt) were always used as control. Gene concept network of GO-term enrichments and associated shared upregulated (**D**) and downregulated (**E**) genes when early treatment of iPSCs occurred (iCM_Dox_Mt and iCM_Dox_Dox). Number of genes, GO-terms, and logFCs is shown.

Next, we asked whether Dox pulse-treatment in the iPSC stage causes persistent changes at the transcriptome level in the resulting iCMs. To analyze this, we performed a pairwise comparison between iCMs treated prior to their differentiation with Dox in their iPSC-stage and their respective control (iCM_Dox_Mt vs. iCM_Mt_Mt). Whole genome transcriptome analysis revealed upregulation of 233 and downregulation of 193 genes (Supplementary Table 3 and Supplementary Data). GO-term enrichment of upregulated genes showed a significant link to biological processes (BP) such as angiogenesis, blood vessel morphogenesis, positive regulation of cell migration, and positive regulation of cell motility (Fig. 8A, Supplementary Fig. 9B). Among these terms, several genes regulating and promoting collagen production have been detected, consistent with a pro-fibrotic shift in gene expression. Indeed, an apparent increase in Collagen III protein levels was observed in Dox-treated iCMs (Supplementary Fig. 11I). Conversely, downregulated genes were enriched in GO-terms related to cardiac muscle tissue development, heart development, and muscle tissue development (Fig. 8B, Supplementary Fig. 9B).

To gain further information on the importance of the time point when Dox treatment occurred, we performed various pairwise comparisons of all treatment conditions in iCMs (iCM_Dox_Mt, iCM_Mt_Dox, and iCM_Dox_Dox), which were all compared to the non-treated control (iCM_Mt_Mt; Fig. 8C).

Initially, we focused on elucidating the influence of Dox treatment in the iPSC stage on the transcriptional response in iCMs. For that, we performed pairwise comparison of treatment conditions sharing an early Dox treatment in iPSC stage (iCM_Dox_Mt and iCM_Dox_Dox both compared to the non-treated control iCM_Mt_Mt) (Fig. 8C). Interestingly, 272 differentially expressed genes were specifically shared, of which 139 genes were upregulated and 133 were downregulated (Fig. 8C, Supplementary Table 4). GO-term enrichment of biological processes (BP) revealed that upon early pulse-treatment with Dox, again genes involved in blood vessel morphogenesis, ossification, extracellular matrix organization, and extracellular structure organization were robustly upregulated (Fig. 8D; Supplementary Fig. 8M-P), namely independent of a Dox-treatment applied in the iCM stage, emphasizing that this transcriptional response is caused before differentiation is initiated. Key genes included Decorin (*DCN*), WT1 Transcription Factor (*WT1*), Filamin A (*FLNA*), and Basonuclin Zinc Finger Protein 2 (*BNC2*), which all have been described to play pivotal roles in regulation of fibrosis and cardiac regeneration. The upregulation of *WT1* and *DCN* was confirmed by qPCR including a treatment regimen with equal doses at the iPSC and iCM stage (iCM_18 nM DOX_18 nM Dox) further supporting our findings from the RNASeq analysis (Supplementary Fig. 11A-H).

On the contrary, treatment of cells in iPSC stage is accompanied with strong downregulation of genes associated with cardiac muscle tissue development, striated muscle cell differentiation, muscle tissue development, and muscle cell development (Fig. 8E; Supplementary Fig. 8A-D). Among these GO-term functional enrichments, NK2 Homeobox 6 (*NKX2-6*), Ryanodine Receptor 1 (*RYR1*), Ankyrin Repeat Domain 1 (*ANKRD1*), and Actinin Alpha 3 (*ACTN3*) have shown to be the most suppressed genes upon Dox treatment before cardiac differentiation. All these mentioned genes are essential regulators of cardiac function. Comparing our findings with publicly available heart tissue RNASeq data from mice that have been treated with Dox or untreated we found a clear upregulation of fibrosis/extracellular matrix markers and a partial reduction in cardiac markers upon Dox treatment in line with our observations (Supplementary Fig. 14A^42^). Consistent with this, also a mouse model of acute Dox-induced toxicity showed a similar tendency of reduced expression upon Dox exposure of reduced expression of two cardiac markers (*Tnnc1* and *Tnnc2*) and also of genes linked to energy metabolism (*Gpi*, *Ndufs8*, *Cox5b*, *Ckm*) (Supplementary Fig. 13), yet these changes were statistically not significant^43^. Overall, we conclude that treatment with a moderate and transient dose of Dox at an iPSC stage prior to cardiac differentiation is sufficient to cause a major transcriptional reprogramming persistent in differentiated cells altering blood vessel formation and promoting fibrosis, while expression of genes required for cardiac muscle regeneration are repressed. This would be in line with a reduced regenerative capacity when iPSCs have been exposed to even low and transient doses of Dox once before the differentiation or regeneration process has been initiated.

### Pulse-treatment of iCMs results in transcriptional adaptations promoting apoptosis

Based on the major impacts seen on the expression profile when cells were pulse-treated prior to cardiac differentiation, we asked how a “late” Dox pulse-treatment at the iCM stage, as opposed to the iPSC stage, influences the transcriptional response in terminally differentiated iCMs. For this, we focused on shared genes that are significantly differentially regulated when iCMs were treated late (iCM_Mt_Dox and iCM_Dox_Dox, both compared to iCM_Mt_Mt (Fig. 8C). The number of shared genes differentially regulated upon late treatment was much lower compared to the shared genes upon early treatment (Fig. 8C, Supplementary Table 4). Only 25 genes were specifically shared, of which 12 genes were upregulated and 13 genes were downregulated (Fig. 8C, Supplementary Table 4 and Supplementary Data). GO-term analysis of upregulated genes showed an enrichment in biological processes (BP) associated with positive regulation of programmed cell death, positive regulation of apoptotic process and apoptotic signaling pathway (Supplementary Fig. 6A). Among these functional enrichments Fas cell surface death receptor (*FAS*), cyclin dependent kinase Inhibitor 1 A (*CDKN1A*), and BCL2 associated X (*BAX*) were among the upregulated genes with highest fold change. All of these candidates play a regulative role in cell survival or cell cycle progression. A similar upregulation of apoptosis-linked genes (*BAX* and *FAS*) upon Dox exposure was also seen in the aforementioned mouse RNASeq data set (Supplementary Fig. 14A). Overall, exposure of terminally differentiated iCMs to a modest dose of Dox is accompanied with a transcriptional upregulation of apoptotic pathways consistent with a direct toxicity of Dox to iCMs promoting apoptosis. The fact that we do not see this apoptotic signature in iPSCs treated with Dox is consistent with our results described earlier showing neither induction of extensive DDR, ROS, nor of apoptosis.

### The transcriptional response of iCMs to doxorubicin is influenced by an early pulse-treatment of Dox in iPSCs resulting in suppression of energy metabolism and muscle contraction pathways

Since iCMs that have been exposed to Dox before cardiac differentiation showed a strong transcriptional response and as these apparently also had multiple functional consequences as described above, we analyzed in more detail whether this early pulse-treatment alters the stress response against Dox when iCMs were exposed for a second time. We find 300 genes that are only differentially regulated upon double-treatment with Dox (iCM_Dox_Dox), of which 164 genes were upregulated and 136 genes were downregulated (Fig. 8C, Supplementary Table 4, Supplementary Fig. 9D). In contrast, iCMs that were only exposed to late Dox (iCM_Mt_Dox) treatment showed 119 different genes specifically affected, emphasizing that the transcriptional response to a late Dox treatment is highly influenced by a type of priming prior to commitment to cardiac differentiation. This is also corroborated by the fact that only 25 genes showed a specific common response upon late treatment. GO-term analysis of significantly upregulated genes upon a second Dox treatment solely found in primed iCMs (iCM_Dox_Dox) showed enrichment of biological processes (BP) such as ERK1 and ERK2 cascade, positive regulation of cell motility, signal transduction by p53 class mediator, and gland development (Supplementary Fig. 6B). Representative genes included cyclin dependent kinase 1 (*CDK1*), Wild-Type P53-induced phosphatase 1 (*PPM1D*), snail family transcriptional repressor 2 (*SNAI2*), and SRY-Box transcription factor 9 (*SOX9*), which have critical regulatory roles in cell fate decisions and transcription. However, priming of iCMs already in iPSC stage was accompanied with downregulation of genes associated with energy metabolism, muscle system processes and muscle contraction (Supplementary Fig. 6C). Within these functional categories, genes such as hexokinase 1 (*HK1*), several respiratory chain complex subunits (*COX5B*, *UQCRC1*, *NDUFS2*, *NDUFS8*), NK2 homeobox 5 (*NKX2-5*), ATPase Na + /K+ transporting subunit beta 1 (*ATP1B1*), and triadin (*TRDN*) were downregulated upon pulse-treatment with Dox. Dysregulation of these genes points to a possible disturbance in energy metabolism and regulation of electrochemical gradients necessary for cardiac function. Overall, we conclude that early doxorubicin treatment at the iPSC stage causes not only a major reprogramming of iCMs themselves but also alters their transcriptional response to a second exposure to Dox markedly negatively impacting pathways linked to energy metabolism and muscle contraction.

### Pulse-treatment of Dox is generally accompanied by transcriptional adaptations associated with suppressed cardiac muscle formation in iCMs

To determine whether Dox exerts a common transcriptional response which might be shared independent of the time or differentiation status of exposure, we identified DEGs commonly regulated over all treatment conditions (iCM_Dox_Mt, iCM_Mt_Dox, iCM_Dox_Dox) each compared to the mock-control (iCM_Mt_Mt). In total for all pulse-treatment schemes of Dox, 870 significantly differentially regulated genes were found (Fig. 8C). Interestingly, only 35 genes were shared between all the treatment conditions, indicating a common Dox-specific effect on gene expression independent of the time point or differentiation status of treatment (Supplementary Table 4 and Supplementary Data). Within the intersection of 35 selectively shared genes, some genes were antagonistically regulated (Supplementary Fig. 7). Enrichment analysis of GO-terms of biological processes (BP) showed no significant term enriched in the number of significantly upregulated genes. In contrast, GO-term enrichment analysis of downregulated genes revealed critical functions such as myofibril assembly, striated muscle cell development, heart contraction, and heart process (Supplementary Fig. 6D). Among these enriched functions, Myosin Heavy Chain 6 (*MYH6*), Nebulette (*NEBL*) and Myosin Light Chain 9 (*MYL9*) were downregulated upon pulse-treatment indicating a time-independent adverse effect of Dox treatment even upon modest and short exposure. In sum, pulse-treatment(s) with modest doses of Dox induce a convergent transcriptional downregulation of essential cardiac muscle genes in iCMs, indicating an impairment of the contractile function of iCMs independently of the Dox exposure timing.

### Pulse-treatment of iPSCs with doxorubicin prior to differentiation results in functional changes in beating iCMs

**Fig. 9:**
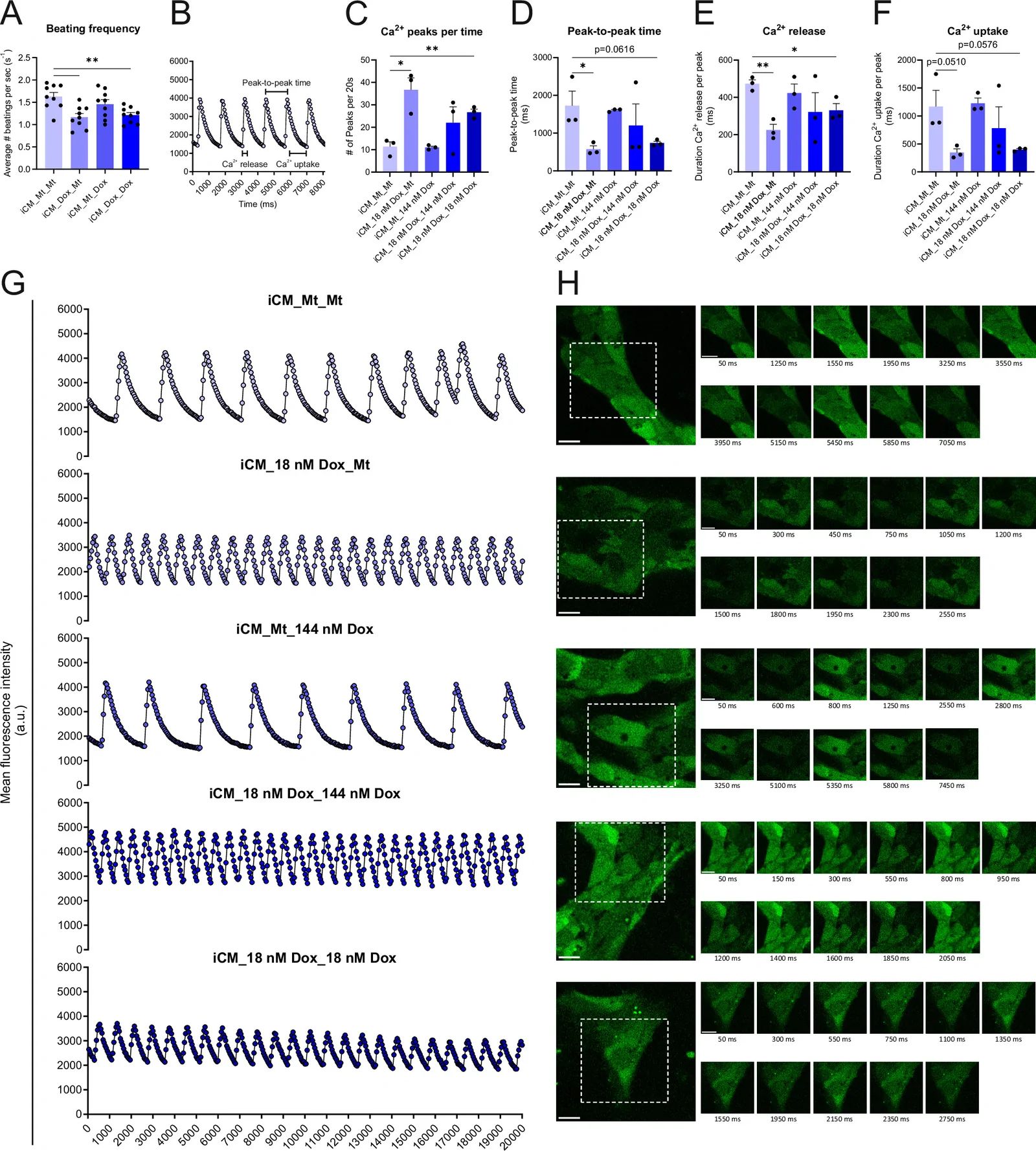
Repeated short pulse-treatment of cells with Dox is associated with impairment of contractility and Ca^2+^ oscillations in iCMs. **A** Average beating frequency per second measured in iCMs short-pulse treated with Dox at different stages of cardiac differentiation. Analysis of iCM contractility parameter in untreated iCMs (iCM_Mt_Mt), iCMs treated in iPSC stage (iCM_Dox_Mt), iCMs treated on day 7 of cardiac differentiation (iCM_Mt_Dox) and in double-treated iCMs (iCM_Dox_Dox). iCMs were treated for 2 h with Dox at defined stages of cardiac differentiation as shown in Fig. 6C. On day 9 of cardiac differentiation protocol, live-cell videos of about 20 s length with 60 frames per second of all conditions were acquired using Leica DM IL LED Fluo Cellfactory microscope. Analysis of the videos was conducted by using an automated analyzing system as described in the method section. Data shown represents the mean of three independent experiments ± SEM (*n* = 3, *N* = 3). One-way ANOVA with multiple comparisons was performed. **p-value ≤ 0.01. **B** Schematic illustration of the determined Ca^2+^ handling parameters in iCMs pulse-treated with Dox at defined time points of cardiac differentiation. Quantitative analysis of Ca^2+^ peaks within the 20 s of recording. Peaks were determined for values of Ca^2+^ signaling intensities higher than 30% of the maximal intensity and were counted as one Ca^2+^ signaling event. *n* = 3; *N* = 25 ROIs). Quantitative analysis of the number of peaks per 20 s (**C**) peak-to-peak time (**D**) calcium release into the cytoplasm (**E**) and uptake into calcium hubs (**F**) in iCMs pulse-treated with doxorubicin at different stages of cardiac differentiation. Shown are the mean ± SEM of three independent biological replicates (*n* = 3; *N* = 25 ROIs). Unpaired Student’s *t* test was performed. *p-value ≤ 0.05, **P-value ≤ 0.01. Live-cell FastAiryScan microscopy of iCMs labeled with Fluo4-AM to monitor intracellular Ca^2+^ fluctuation in iCMs with indicated treatment regimens. Exemplary intensity changes representing Ca^2+^ oscillations are shown (**G**) for each condition as indicated and exemplary fluorescent images at selected time points of minimal and maximal Ca^2+^ signal intensities are shown at indicated time points (**H**). The cropped panel below, based on the zoomed region, is indicated by a white box. Scale bar: 10 µm.

To evaluate whether the transcriptional changes showing impaired cardiac function translate into functional impairments related to muscle contraction, we analyzed the spontaneous beating frequency of all four Dox treatment groups based on live-cell videos of iCMs (Fig. 9A, Supplementary Movies 1-4) using the software Cardiovision^44^. Overall, quantitative analysis revealed a significant reduction in the beating frequency in those iCMs that where “early” pulse-treated with Dox at the iPSC stage and in double-treated cells (Fig. 9A). Thus, priming of iCMs appears to have a more profound functional effect than treating terminally differentiated iCMs with Dox that did not experience a Dox exposure at the iPSC stage. Cardiomyocyte contractility is well known to involve oscillating Ca^2+^ release from the ER, and other Ca^2+^deposits including mitochondria, and consecutive Ca^2+^ uptake, a highly energy-consuming cellular process. Thus, we asked whether Ca^2+^ handling in iCMs is affected in our five different treatment regimens (Fig. 6A) using a Ca^2+^-sensitive dye (Fluo4-AM) and Fast AiryScan fluorescence imaging (Fig. 9G, H). We could determine robust and rhythmic Ca^2+^ oscillations in all conditions (Fig. 9G, H). The number of Ca^2+^ peaks per time was significantly (~2- to 3-fold) increased in iCMs receiving 18 nM Dox at the iPSC stage (Fig. 9B, C). The most profound effect was observed for iCMs that were only treated once at the iPSC stage. Consistently, the average “peak-to-peak” times were markedly reduced (Fig. 9D). In principle, this was also observed when we analyzed in more detail the average duration of Ca^2+^ release per peak showing that early Dox treatment accelerated this parameter compared to untreated controls (Fig. 9E). For Ca^2+^ uptake the same trend was observed, however this was not statistically significant (Fig. 9F). Late treatment (iCM_Mt_144 nM Dox) had no significant effect on Ca^2+^ handling (Fig. 9B–H). We further asked whether an early encounter with Dox at the iPSC stage might be accompanied by a higher sensitivity of iCMs against Dox using the SRB assay for cell viability. Yet, we did not observe significant changes and only noted a moderately higher sensitivity against a second Dox dose when cells were primed (Supplementary Fig. 4B).

In accordance with our transcriptome findings, even a single short pulse-treatment with a moderate dose of Dox enhances dysregulation of cardiac contractility and of Ca^2+^ handling in iCMs derived from these iPSCs. This effect was in all cases quite pronounced even with a single Dox dose applied at the iPSC stage. Overall, we clearly demonstrated that primed cells are functionally altered and we propose that this is linked, at least in part, to mitochondrial dysfunction as well as to the major transcriptional reprogramming described here promoting fibrosis on the one hand side and impairing cardiac contraction function on the other side.

## Discussion

Dox is a widely-used and effective chemotherapeutic drug with severe short- and long-term adverse cardiotoxic effects limiting its clinical application. Understanding the underlying pathomechanisms of this genotoxic drug is essential to develop strategies preventing heart failure and/or promoting heart regenerative processes. Here we have established and characterized a model system comprising of human iPSCs and iPSC-derived cardiomyocytes (iCMs) allowing us to study cellular and transcriptional consequences of moderate Dox pulse-treatment regimens at defined stages of cardiac differentiation. The strength of this model system is that it enables the dissection of the pleiotropy of consequences inflicted by a moderate exposure to Dox encountered prior to differentiation in a defined, differentiated cell type, namely cardiomyocytes. In particular, we aimed to understand the consequences of moderate Dox treatment conditions on mitochondrial and metabolic functions, how this impacts cardiac differentiation, and whether pre-conditioning at an iPSC stage influences the susceptibility of their progeny. In contrast to many earlier studies, we applied a treatment scheme including rather mild and short exposures taking into account the low Dox concentrations reached rapidly in the plasma of treated patients receiving Dox as fast infusion^15^. Our study gives the following novel insights. First, human iPSCs are highly sensitive, much more than iCMs, to this rather mild Dox-treatment regime. Exposure to low concentrations of Dox for only 2 h was sufficient to reduce the viability of iPSCs significantly whereas iCMs were much more resistant. Second, this mild treatment primarily led to multiple defects in mitochondrial functionality including increased mitochondrial fragmentation, enhanced OPA1 processing, reduced inner membrane potential and altered mtDNA genome organization. The latter is consistent with the known genotoxic role of Dox by inhibiting mitochondrial DNA replication via intercalation as well as inhibition of the mitochondrial DNA-Topoisomerase IIβ. Importantly, it should be emphasized that these mild conditions did neither result in increased ROS levels, nor in activating a DDR or cell death, at least to a detectable manner, allowing to differentiate between toxic effects of Dox on mitochondrial DNA versus nuclear DNA. Third, upon analyzing the genome-wide transcriptional response to Dox at different time-points of differentiation we observed that iPSCs show an entirely different response to Dox than iCMs and much more genes were differentially expressed. Forth, another striking finding in our view is that iCMs treated at the iPSC stage, which we term priming, have a very distinct transcriptional expression profile compared to iCMs that are not primed. Interestingly, exactly those pathways that are linked to cardiac regeneration are reduced and pathways that promote fibrosis are increased in cells that have been exposed to Dox prior to differentiation. Also, metabolic pathways were differentially affected. Fifth, when analyzing the functional consequences of priming at the iPSC stage we observed that such iCMs show reduced ATP levels, altered mtDNA organization, and were severely impaired regarding muscle contractility and Ca^2+^ handling.

Overall, this demonstrates for the first time that pluripotent, non-differentiated cells are genetically reprogrammed when exposed to a genotoxic substance in a way that does not alter differentiation per se but does alter the capacity to regenerate cardiac muscle. The observation that stem-cells exhibit high sensitivity against Dox exposure, albeit DDR is not induced, strongly support that primarily mitochondrial impairments lead to cellular dysfunction and suggests that mitochondria are one prime target for Dox, even at very low concentrations and in particular for pluripotent stem cells. Moreover, mitochondrial defects, including alteration of mtDNA organization and ATP production, remain detectable later in the progeny of iPSCs demonstrating a cellular memory of those early events giving rise to functional alterations that are concomitant with substantial transcriptional reprogramming.

Here, the application of mild and transient exposures of a genotoxic compound revealed a number of unexpected insights compared to earlier studies using rather high doses for longer time periods. For example, a high susceptibility of stem cells to undergo programmed cell death was reported and has been proposed to ensure the maintenance of the stem cell pool by elimination of genetically defect cells^45^. Moreover, stem cells were proposed to rely strongly on complex DNA repair machineries to encounter possible DNA damage^46^. We do not question these findings; however, it appears that this cannot be generalized for milder treatment conditions. At the conditions applied here, an induction of apoptosis upon treatment with Dox was not observed. Further investigation of DNA damage repair (DDR) of iPSCs in our setting revealed no detectable DDR induction or signs of DNA damage. Thus, the high sensitivity of iPSCs has to be based on other factors, yet not on induction of persistent DNA damage or insufficient DNA repair.

We demonstrate, that mitochondria are one major target of Dox-induces and propose that they at least contribute to explain our findings. Besides the genomic DNA in the nucleus, mitochondria also harbor their own DNA (mtDNA) in multiple copies per cell and represent another target of Dox known to intercalate into DNA and to inhibit various topoisomerases. In mammalian cells, mitochondria constantly undergo fusion and fission events necessary for the sensitive regulation of cell homeostasis and rapid response upon cellular injuries^47^. Thus, morphological changes towards fission into single mitochondria has been associated with an increasing susceptibility of cells for apoptosis^48,49^. Along with morphological changes, previous studies have shown dysfunction of mitochondria, such as dissipation of mitochondrial membrane potential and reduction in oxygen consumption after treatment of iPSC-derived cardiomyocytes (iCMs) with Dox^50^. It has to be considered that the observed mitochondrial dysfunction was assessed using comparatively high doses of Dox with chronic treatment schemes. Therefore, it remains unclear whether in these cases mitochondrial damage was caused by a direct effect of Dox or by the accumulation of nuclear DNA damage. Here, we show that even low-dose pulse-treatment of iPSCs with Dox is sufficient to induce a shift in mitochondrial morphology towards fragmentation in iPSCs, yet in iCMs this shift is less pronounced and is partially reversed upon priming at iPSC stage (Supplementary Fig. 4C,D). It is worth to note, that mitochondrial morphology differs between iPSC cell lines depending on the genetic background of somatic origin^51^. According to previous studies, the iPSC line iPS (IMR90)-4 showed mostly tubular mitochondrial phenotype^52^. Besides morphology, mitochondrial dynamics and mitochondrial membrane potential are strongly linked to the energetic state of the cell^53,54^. Although iPSCs mainly depend on glycolysis as major pathway of energy conversion^55,56^, mitochondrial respiration can still be evaluated. Using live-cell respirometry, we clearly demonstrated that low Dox concentrations led to a decrease in mitochondrial oxygen consumption rates and in parallel caused a loss of mitochondrial membrane potential in iPSCs. The formation of crista via invagination of the inner mitochondrial membrane into the matrix space is essential for providing an assembly platform for OXPHOS complexes^57,58^. Since chronic exposure of cells with Dox is reported to be accompanied with disruption of mitochondrial ultrastructure^59^, we further elucidated the influence of acute Dox treatment on the ultrastructure of mitochondria in iPSCs. Contrary to our expectations, pulse-treatment had no observable effect on mitochondrial ultrastructure as well as OXPHOS complex assembly and in-gel activity. Nevertheless, to our knowledge we showed for the first time the presence of OXPHOS supercomplexes in iPSCs. Dox is able to cross both mitochondrial membranes and to intercalate into the 16.5 kb-sized mitochondrial DNA (mtDNA) encoding 13 essential OXPHOS complex subunits^60,61^. Compared to nuclear DNA, mtDNA is not surrounded by histones protecting DNA against possible damage. Interestingly, it has been shown that upon chronic exposure to Dox, nucleoids start to condensate^62^. This was suggested to rather serve as a protective way to reduce intercalation of Dox into mtDNA under chronic exposure condition than to be a consequence of Dox mtDNA intercalation. Our study shows that even a mild and transient treatment with Dox is accompanied with a remodeling of mtDNA nucleoids, which apparently occurred and persisted for 48 h after treatment. This imminent change in nucleoid size and number rather argue for a direct effect of Dox on mtDNA organization. This short time presumably also explains why we did not see any reduction of mtDNA and also no major changes in mitochondrial protein levels linked to mtDNA organization or OXPHOS. At this point we still cannot say whether mtDNA replication or the turnover rate of mtDNA was affected in any way upon Dox treatment. Further studies are needed to investigate the role of mtDNA turnover and possible effects on mtDNA replication in regulating cell fate in iPSCs. Besides adverse effects of Dox on stem cells in the organism, cardiomyocytes have been reported to be a cell type strongly impaired upon Dox treatment. The model used here allowed us to further characterize iCMs avoiding the problem of human or animal studies using cardiac tissue harboring multiple, very diverse, cell types. Our study reveals that iCMs are not only less susceptible towards pulse-treatment of Dox as compared to their undifferentiated counterparts, but also an early exposure to Dox lateron affects iCMs on a functional and on a regulatory transcriptomic level. Functionally, we observed that primed iCMs show alteration of mtDNA organization, reduced ATP levels, and enhanced ATP-linked respiration/ATP production. Moreover, we found that this is correlated to upregulation in genes associated with structural changes of the extracellular matrix often linked to increased fibrosis. In particular, the upregulation of collagen and other structure-determining genes in iCMs were shown to be associated with several cardio dysfunction^63^. Additionally, transcriptomic analysis revealed a consistent adverse effect of early pulse-treatment with Dox on genes essential for cardiac function, such as contractility and calcium homeostasis. This is in accordance to an earlier multi-omics study investigating the effect of chronic Dox exposure to iCMs^64^. Importantly, these observed changes in the transcriptome are apparently functionally translated as we observed reduced muscle contractility and altered Ca^2+^ oscillations under these conditions. Whether and how these changes are mechanistically linked to the observed mitochondrial alterations in energy metabolism and mtDNA organization remains to be deciphered in future studies.

Overall, priming at the iPSC stage was associated with reduced expression levels of genes involved in glycolytic processes indicating a possible metabolic switch in energy conversion in iCMs after priming towards OXPHOS. This would be in line with our findings that mitochondrial ATP production/ATP-linked respiration is increased. Moreover, we propose that such a stimulation of ATP production is a compensatory mechanism that would permit increased ATP demands expected from the observed increased oscillation rates of Ca^2+^ release and uptake. It is known that not fully matured iCMs inherit characteristics of fetal cardiomyocytes maintaining a residual proliferative capacity^65,66^. This suggests a high dependency of immature iCMs on glycolysis to fulfill their rudimentary energy demand. Thus, it is possible that such a metabolic shift induced by priming may impair the regenerative and proliferative capacity of fetal cardiomyocytes.

In contrast, upon late Dox treatment expression of genes involved in apoptotic processes was enhanced. This was in accordance with a previous study where Dox treatment induced the upregulation of death receptors in cardiomyocytes^67^.

Taken together, pairwise comparisons of different schemes of Dox treatment allowed us to determine a time point scheme-specific transcriptome “fingerprint” demonstrating that priming of iPSCs causes a broad and persistent genome-wide reprogramming of transcription profiles. Interestingly, many of the pathways that are negatively affected are required for cardiac function and many of the pathways that are positively affected are promoting fibrosis. These changes apparently do well manifest in several functional changes. Moreover, we also included a double-treatment scheme to investigate possible hormesis effects. Here, we aimed to prime iCMs with moderate doses of Dox at the iPSCs stage. It has been shown that mild ischemia pre-exposure of the heart was beneficial in overall cell survival upon a subsequent ischemic insult, which was termed preconditioning^68^. Albeit the term preconditioning is often linked to a sublethal stress having protective and beneficial outcomes, termed “hormesis”, this is not always the case and can strongly depend on the parameter investigated. Sublethal forms of stress can also have negative effects as shown e.g., for hepatocytes obtained from ischemia-preconditioned donor livers which had increased oxidative damage and reduced proliferative capacity compared to controls^69^. Here, since we observed negative and partly also positive effects we decided to use the term priming in a rather neutral manner, which does not generally infer positive or protective outcomes upon a second stress encounter. Indeed, our study did show both beneficial and detrimental effects of priming depending on the parameter tested. A possible hormesis effect was observed regarding ATP production/ATP-linked respiration as in primed iCMs a second Dox dose did not cause its reduction and rather reversed the effect. Such a protective effect was similarly found for changes in maximal respiration and changes in mitochondrial morphology. However, with respect to muscle contractility and Ca^2+^ handling we did not see any beneficial effects suggesting rather an adverse or cumulative effect on cardiac function upon priming of cells. In accordance to these findings, it was prominent that solely iCMs that received priming showed an increased sensitivity against Dox. Albeit our model system using iPSCs and iCMs has a clear limitation for any in vivo conclusions, it is worth to discuss our findings in this context, as they are reminiscent of earlier results reported for an in vivo model of late-onset Dox-induced cardiotoxicity in mice^70^. Here it was shown that juvenile mice which have been exposed to a dose of Dox that did not cause acute cardiotoxicity, were much more sensitive to stress-induced cardiotoxicity as adult mice compared to non-treated mice. Primed mice when subjected to myocardial infarction showed impaired vessel formation and fewer progenitor cells in the infract border region compared to mice that were not treated with Dox at the juvenile age. This is in line with our model that a pool of progenitor cells, which can be non-cardiac pluripotent cells or a subset of CMs that have the potential to dedifferentiate and redifferentiate, are altered negatively by a single mild exposure to Dox at an early stage of differentiation. Whether mitochondrial function is impaired in this in vivo model will be an interesting question to be addressed in the future. The facts that late-onset cardiotoxicity after Dox-treatment in humans is more prominent in children than in adults^2,3,7^ and that in children the percentage of cardiac cells renewing per year is much higher than in adults^14^ is consistent with the view that in particular affecting mitochondrial function in a pool of cardiac stem cells contributes to the high degree of late-onset cardiotoxicity in children. This also fits to the emerging view that mitochondrial quality control is critical to maintain cardiac function during ageing and also after cardiac damage^24^.

Overall, we demonstrate that even mild pulse-treatment of iPSCs with Dox is associated with persistent mitochondrial dysfunction and transcriptional reprogramming that affect cardiomyocyte function after differentiation. Human induced-pluripotent stem cells (iPSC) were introduced as a sensitive cell model system for investigation of the underlying susceptible processes upon Dox exposure. Apart from the time point of treatment, pulse-treatment of iCMs revealed changes in cardiac function and fibrosis as supported by our functional and transcriptome profiling data. Our findings highlight the importance of the exposure timing and suggest that the stem cell compartment is particularly vulnerable to genotoxic stress, with long-lasting consequences that manifest after cardiac differentiation. As Dox is one of the most commonly used chemotherapeutic agents across different age groups, the question arises whether these persistent changes are responsible for the frequently observed side effects. As elaborated above, although there is still a discussion regarding the origin of cells repopulating damaged regions of the heart and the driving factors promoting heart regeneration in general, it is evident that some type of pluripotent cardiac or non-cardiac stem cells or a subset of cardiomyocytes themselves dedifferentiating and redifferentiating exist and can promote cardiac regeneration^25,28,30,71^. We propose that an enhanced sensitivity of any of these pluripotent cells to Dox treatment affecting mitochondrial functions can contribute to the manifestation of the adverse side effects especially in pediatric patients. Thus, future studies using iPSC-based models as well as animal and human data are needed to decipher how different types of stem cells are transcriptionally primed in-vivo after an initial exposure to sublethal doses of Dox or other genotoxic substances and how this affects mitochondrial function, heart failure and heart regeneration.

## Methods

### Cell lines

Human fetal-derived iPSC line iPS (IMR90)-4 (lentiviral transfection with *Oct4*, *Sox2*, *Nanog* and *Lin28*, WiCell) were used for experimental procedures^72^. iPSCs were cultured in feeder-free conditions on Geltrex™-coated plates (Thermo Fisher Scientific Inc.) using mTeSR™ Plus media (STEMCELL Technologies Inc.) supplemented with 1% Penicillin/Streptomycin. Cells were kept under normal oxygen conditions at 37 °C and 5% CO_2_ with media change every second day. At 80% confluency, iPSCs were passaged as single cells at a confluency of 80% using StemPro™ Accutase™ (Thermo Fisher) and a defined number of cells was seeded for the respective experiments. To enhance viability of iPSCs after passaging, media was supplemented with 10 µM ROCK inhibitor Y-27632 2HCl (AdooQ BioScience) for 24 h followed by media change the next day and subsequent media changes every second day.

The human fetal-derived iPSC line iPS (IMR90)-4 was purchased from a commercial vendor (WiCell; https://www.wicell.org/home/stem-cells/catalog-of-stem-cell-lines/ips-imr90-4.cmsx) and described in an earlier publication^72^. This cell line is registered as WISCi004-B in the hPSCreg database (https://hpscreg.eu/cell-line/WISCi004-B) and according to public available information on this website ethical approval/informed consent has been obtained from the donor of the embryo/tissue (sex female, fetal 16 weeks) from which this cell line originated. Human iPSC11 and iPSC12 cell lines from Alstem (episomal plasmid with OCT4, SOX2, KLF4, and L-MYC) were used for experimental procedures. iPSCs were cultured in feeder-free conditions on Geltrex™-coated plates (Thermo Fisher Scientific Inc.) using mTeSR™ Plus media (STEMCELL Technologies Inc.) supplemented with 1% Penicillin/Streptomycin. Cells were kept under normal oxygen conditions at 37 °C and 5% CO_2_ with media change every second day. At 80% confluency, iPSCs were passaged with StemPro™ Accutase™ (ThermoFisher). To enhance the viability of iPSCs after passaging, media were supplemented with 10 µM ROCK inhibitor Y-27632 2HCl (AdooQ BioScience) for 24 h.

### Cardiac differentiation

iPSC-derived cardiomyocytes (iCMs) were generated using published protocols^73,74^. Briefly, 8 × 10^5^ cells per well were seeded as single cells with StemPro^TM^ Accutase^TM^ (ThermoFisher) on Geltrex^TM^-coated (Thermo Fisher Scientific Inc.) 24-well plates (Greiner Bio-One GmbH) and 10 µM ROCK inhibitor Y-27632 2HCl (AdooQ BioScience) was added for 24 h followed by media change the next day and subsequent culturing according to the cardiac differentiation protocol. Upon 80% confluency, cardiac differentiation was initiated by complete aspiration of stem cell media and exchange with basal cardiac differentiation media (CBM) consisting of RPMI 1640 media without L-Glutamine (Capricorn Scientific) supplemented with 1% Penicillin/Streptomycin (Sigma-Aldrich), 2 mM stable Glutamine (Capricorn Scientific) and 25 mM HEPES buffer (Thermo Fisher). During the process of cardiac differentiation, media was completely aspirated and exchanged by the respective cardiac differentiation media. From day 0 to day 2, 1x B-27 supplement without insulin (ThermoFisher), 50 µg/mL ascorbic acid (Sigma-Aldrich) and 6 µM CHIR99021 (MedChemExpress) was added to CBM. On day 3 of the cardiac differentiation, CBM media was supplemented with 1x B-27 supplement without insulin (ThermoFisher), 50 µg/mL ascorbic acid (Sigma-Aldrich). From day 4 to day 5, 1x B-27 supplement without insulin (ThermoFisher), 50 µg/mL ascorbic acid (Sigma-Aldrich) and 5 µM IWP4 (STEMCELL Technologies Inc.) was added to CBM. From day 5 to day 7 the same media composition was used as described for day 3. From day 7, cells received CBM supplemented with 1x B-27 supplement (ThermoFisher) and 50 µg/mL ascorbic acid (Sigma-Aldrich). Expression of cardiac differentiation markers was checked on the respective days of analysis with quantitative real-time PCR. Primers used are listed in Supplementary Table 1.

### Cell viability assay

To determine the cell viability of iPSCs and iCMs 48 h after 2 h pulse-treatment with Dox cells MTT assay (Sigma-Aldrich) was performed. For iPSCs, 6–8 × 10^4^ cells were seeded on Geltrex^TM^- coated 96-well plates (Greiner Bio-One GmbH). For iCMs, 6 × 10^4^ cells were split as single-cell suspension on Geltrex^TM^- coated 96-well plates (Greiner Bio-One GmbH). MTT assay measures the reduction of the terazolium salt 3-(4,5-dimethylthiazol-2-yl)-2,5-diphenyltetrazolium bromide by NAD(P)H-dependent oxidoreductase enzymes to purple formazan^75^. Absorbance was measured at 565 nm using Tecan INFINITE 200 Pro (Tecan Austria). Relative cell viability in respective controls was set to 100%. In addition, cell viability was measured with Sulforhodamine B assay in iPSCs (SRB assay, Supplementary Fig. 1) and iCMs (Supplementary Fig. 4B), respectively. Absorbance was measured using Tecan INFINITE 200 Pro (Tecan Austria). Relative cell viability in respective controls was set to 100%. To determine cell viability of iPSC11 and iPSC12 cell lines, 2 × 10^4^ cells were seeded as single-cell suspension, on Geltrex™-coated 48-well plates (Greiner Bio-One GmbH). 72 h after splitting, cells were treated with doxorubicin (1–2048 nM) for 2 h followed by a recovery time of 48 h. Viability was determined via measurement of the reduction of the terazolium salt 3-(4,5-dimethylthiazol-2-yl)-2,5-diphenyltetrazolium bromide by NAD(P)H-dependent oxidoreductase enzymes to purple formazan^75^. Absorbance was measured at 565 nm using Tecan INFINITE 200 Pro plate reader (Tecan, Austria). Relative cell viability in respective controls was set to 100%.

### Gene expression analysis (RT-qPCR)

Total RNA was purified using RNeasy Mini Kit (Qiagen). Reverse transcription was performed with GoScript™ Reverse Transcription Mix, Oligo(dT) kit (Promega) using 1 µg RNA. qRT-PCR was performed as followed: 1.25 °C—5 min; 2. Extension at 42 °C—60 min; 3. Inactivation with 70 °C—15 min and holding of the samples at 4 °C. Quantitative analyses were performed in triplicates using Corbett Rotorgene RG-6000 (Corbett Research) and the GoTaq® qPCR Master Mix (Promega). Quantitative real-time PCR (qRT-PCR) was performed as follows: 1. 95 °C—2 min; 2. Amplification for 40 cycles with 95 °C—5 s, 60°C—10 s; 3. Melting curve analysis with 1 °C steps ramping from 65 to 95 °C. Primers for qRT-PCR are listed in Supplementary Table 1 and Supplementary Table 2. At the end of each run, melting curves were analyzed. PCR products with Cq-values ≥ 35 were neglected. *HPRT1* was used as a housekeeping gene. For mtDNA:gDNA ratio, *GAPDH* was used as housekeeping gene. Relative mRNA expression was set to 1.0 for the untreated controls.

For determination of expression levels of cardiac, fibrotic and mitochondrial encoded genes in iCM culture upon doxorubicin treatment, 9.5 × 10^5^ iPS (IMR90)-4 cells per well were seeded onto Geltrex™-coated 6-well plates (Greiner Bio-One GmbH) followed by procedure of cardiac differentiation protocol. Expression of fibrotic markers was determined in following samples: Untreated iCMs (iCM_Mt_Mt), iCMs treated with 18 nM Dox in iPSC stage (iCM_ 18 nM Dox_Mt), iCMs treated on day7 with 144 nM Dox (iCM_Mt_144 nM Dox), iCMs treated in iPSC stage with 18 nM Dox, on day7 of cardiac differentiation with 144 nM Dox (iCM_18 nM Dox_144 nM Dox) and iCMs treated in iPSC stage with 18 nM Dox, on day7 of cardiac differentiation with 18 nM Dox (iCM_18 nM Dox_18 nM_Dox). Total RNA was purified using RNeasy Mini Kit (Qiagen) and reverse transcription was performed with GoScript™ Reverse Transcription Mix, Oligo(dT) kit (Promega) using 1 μg RNA. qRT-PCR was performed as followed: 1. 25 °C—5 min; 2. Extension at 42 °C—60 min; 3. Inactivation with 70 °C—15 min and holding of the samples at 4 °C. Quantitative analyses were performed in triplicates using Corbett Rotorgene RG-6000 (Corbett Research) and the GoTaq® qPCR Master Mix (Promega). Quantitative real-time PCR (qRT-PCR) was performed as followed: 1. 95 °C —2 min; 2. Amplification for 40 cycles with 95°C—5 s, 60°C—10 s; 3. Melting curve analysis with 1 °C steps ramping from 65 to 95 °C. Primers for qRT-PCR are listed in Supplementary Table 1. At the end of each run, melting curves were analyzed. *HPRT1* was used as a housekeeping gene. Relative mRNA expression was set to 1.0 for the untreated control.

### Mouse samples

Mouse-derived mRNA samples were kindly provided by our collaborators Prof. Dr. Gerhard Fritz and Dr. Christian Henninger. Samples used for analyses originate from previous publication^43^. In brief, animals were housed in the central animal facility of the Heinrich-Heine-University Duesseldorf (ZETT), experiments were performed according to the European guidelines of Directive 2010/63/EU, approval of animal experiments was granted by the North Rhine-Westphalia State Agency for Nature, Environment and Consumer Protection^43^. Euthanasia was performed by cervical dislocation under isofluorane anesthesia. The acute treated mice received 18 mg/kg BW doxorubicin (i.p.) as a single treatment, and analysis was performed 48 h later. The control group received NaCl injection at comparable time points. Mouse mRNA samples were stored at −80 °C until further procedure. Acute and subacute treated mouse samples were analyzed for gene expression of fibrotic, cardiac and mitochondrial markers. Quantitative analyses were performed in triplicates using Corbett Rotorgene RG-6000 (Corbett Research) and the GoTaq® qPCR Master Mix (Promega). Primers for qRT-PCR are listed in Supplementary Table 2. At the end of each run, melting curves were analyzed. PCR products with Cq-values ≥ 35 were neglected. *Nudc1* was used as housekeeping gene. Relative mRNA expression was set to 1.0 for the respective NaCl control groups.

### RNA-seq database research

Database research was performed with ARCHS4 (**A**ll **R**NA-seq and **Ch**ip-**s**eq **s**ample and **s**ignature **s**earch), a resource that provides access to gene and transcript counts uniformly processed from all human and mouse RNA-seq experiments from the Gene Expression Omnibus (GEO) and the Sequence Read Archive (SRA)^76^. Application of the search term “doxorubicin”, resulted in 83 human studies and 20 mouse studies containing RNA-seq experiments directly tagged with doxorubicin treatment. Received human RNA-seq studies were further filtered for patient samples. Filtering for patient samples resulted in one publication^41^ which was included for further analysis. For mouse studies, following criteria were applied to filter the dataset received from ARCHS4 database research: “in vitro or ex vivo treated murine cardiomyocytes” and “mice treated with doxorubicin”. Application of the filters resulted in four mouse studies which performed RNA-seq analysis of heart tissue from doxorubicin-treated mice. Here, only the study of Luo et al. fulfilled the criteria and was included for further analysis^42^. Heatmaps of mouse and human RNA-seq data were generated using the Bioconducter package ComplexHeatmaps^77,78^. Targeted analysis of OXPHOS genes was performed using a pre-defined gene list from Harmonizome 3.0^39,40^.

### Immunofluorescent staining of cells

To perform immunofluorescence staining, 7.5 × 10^4^ cells per well were seeded as single cells on Geltrex^TM^-coated cover glasses (Carl Roth) of a 6-well plate (Greiner Bio-One GmbH). Briefly, at the day of analysis, cells were fixed with 4% PFA for 15 min at room temperature, followed by a second fixation with 100% methanol for 10 min at −20 °C. Samples were permeabilized and blocked in PBS with 0.5% Triton X-100, 5% BSA (PAN-Biotech) for 30 min at room temperature, and then incubated with primary antibody in PBS with 0.1% Triton X-100, 5% BSA overnight at 4 °C as indicated. Samples were then washed, incubated with secondary antibody for 1 h at room temperature, and mounted with ROTI®Mount FluorCare DAPI (Carl Roth). The following antibodies were used: α-Ki-67 (Cell Signaling, #9449S), α-γH2AX (Cell Signaling, # 80312S), α-53BP1 (Cell Signaling, #4937), α-HSP60 (Sigma-Aldrich®, #SAB4501464-100UG), α-dsDNA (PROGEN, #AC-30-10), goat anti-mouse IgG H&L AlexaFluor®488 (ThermoFisher Scientific, #A27034), goat anti-rabbit IgG H AlexaFluor®647 (ThermoFisher Scientific, #A27040). Fluorescence Z-stack imaging of iPSCs was acquired at ECLIPSE Ti2 inverted confocal microscope (Nikon Instruments Inc.), coupled with UltraVIEW®VoX spinning disc laser system (PerkinElmer Inc.) equipped with a 63× oil objective (N.A. 1.2) using Velocity 6.3 software (PerkinElmer Inc.).

### SDS PAGE and western blotting

For SDS-PAGE, at least 7.5 × 10^4^ cells per well were seeded onto Geltrex^TM^-coated 6-well plates (Greiner Bio-One GmbH). After following indicated treatment regimens, cells were harvested and pellets were frozen at −80 °C. Pellets were thawed on ice and protein was extracted with RIPA lysis buffer. Protein levels were quantified using *DC* Protein Assay Kit (Bio-Rad Laboratories, Inc.). Protein lysates (15 µg) were separated on 15% or 5% SDS-gels, transferred to nitrocellulose membranes (Amersham) and decorated with antibodies as indicated. Signal was visualized with Thermo Scientific™ SuperSignal™ West Femto Maximum Sensitivity Substrate (Thermo Fisher Scientific Inc.) and Fusion SL (Vilber). Intensity of the bands was analyzed using ImageJ 1.53c^79^ and normalized to untreated control. Following antibodies were used: α-PARP (Cell Signaling, #953S), α-Caspase3 (abcam, #ab32351), α-cleaved Caspase 3 (Cell Signaling, #9664S), α-Actin (Thermo Fisher Scientific Inc., #MA1-744), α-γH2AX (Sigma Aldrich®, #SAB5600038-100UG), α-ATM (Cell Signaling, #2873T), α-pATM (Thermo Fisher Scientific Inc., #MA1-46069), α-Vinculin (Sigma-Aldrich®, #V9131-100UL), α-OPA1 (Pineda, polyclonal rabbit antibody custom-made against C-terminal peptide^80^), goat anti-mouse IgG H&L (HRP) (abcam, #ab6789), goat anti-rabbit IgG H&L (HRP) (BIOZOL, # 111-035-144). For the following proteins slight adaptations were used, namely around 9.5 × 10^4^ iPS (IMR90)-4 cells per well were seeded onto Geltrex^TM^-coated 6-well plates (Greiner Bio-One GmbH). After 72 h, cells were treated with 18 nM doxorubicin for 2 h, followed by recovery time for 48 hours. Then, cells were harvested and pellets were frozen at −80 °C until further procedure. Protein was extracted using RIPA lysis buffer RIPA-buffer (50 mM Tris-HCl pH 7.4; 150 mM NaCl; 1% NP-40; 0.5% sodium deoxycholate; 1% Triton X-100) containing cOmplete^TM^ (Roche Deutschland Holding GmbH) and PhosSTOP^TM^ (Roche Deutschland Holding GmbH). Protein lysates (15 μg) were separated on 15% or 5% SDS-gels, transferred to nitrocellulose membranes (Amersham). Membranes were decorated with following antibodies: MRPL28 (Sigma-Aldrich, #HPA030594), MRPS25 (ProteinTech, #15277-1-AP), MRPL3 (ProteinTech, #16584-1-AP), MRPL12 (ProteinTech, #14795-1-AP), MRPL53 (ProteinTech, #16142-1-AP), PEO1/Twinkle (Proteintech, #18793-1-AP), TOM20 (Proteintech, #11802-1-AP), MRPS18B (Proteintech, #16139-1-AP), MTCO1 (abcam, #ab14705), UQCRC2 (abcam, #ab14745), MTCO3 (abcam, #ab110259), NDUFA9 (abcam, #ab147139), Fp complex II/FP CII (Novex, 459200), alpha Tubulin (Sigma Aldrich, #62204), GAPDH (Proteintech, 81640-5-RR), TFAM (gently provided by Prof. Dr. Rudi Wieser), ATP5A (abcam, ab14748), Cytochrome *b/CYTB* (Sigma Aldrich, #AV50256), MTND1 (Proteintech, #19703-1-AP), goat anti-mouse IgG H&L (HRP) (abcam, #ab6789), goat anti-rabbit IgG H&L (HRP) (BIOZOL, # 111-035-144). Signal was visualized with Pierce™ ECL Western Blotting-Substrate (Thermo Fisher Scientific Inc.) and visualized using visualized using a LAS500 CCD camera. Images were analyzed using ImageJ1.53c^79^ and normalized to untreated control.

### Analysis of apoptosis and DNA damage response

For measurement of apoptosis in iPSCs, 7.5 × 10^4^ cells were seeded as single cells onto Geltrex™-coated 6-well plates (Greiner Bio-One GmbH). The pan caspase inhibitor Z-VAD-FMK (AdooQ BioScience) was used in order to limit cell death. Cells were pre-incubated with Z-VAD-FMK for 4 h prior to procedure of Dox treatment regimen. Staurosporine was applied as a positive control. Induction of apoptosis was determined using SDS-PAGE and western blot analysis using indicated markers. Protein levels of each treatment were determined and further evaluated using the software GrapPad Prism8. To assess DNA damage induced by moderate pulse-treatment with Dox, the levels of DNA damage markers phosphorylated histone variant H2A.X (γH2AX, phosphorylated histone H2A.X at serine 139) and P53-binding protein 1 (53BP1) were measured by immunofluorescence staining. Briefly, 2.5 × 10^4^ cells (iPSCs) per well were seeded on Geltrex^TM^-coated coverslips in 24-well plate Greiner Bio-One GmbH) followed by treatment regimen with Dox. Here, 10 nM of etoposide (Eto) treatment was used as a positive control. Cells were then fixed at 0.5, 4-, 8-, and 24-h post-treatment and stained for the respective markers. Subsequent to this, the number of individual γH2AX and 53BP1 foci was manually counted using ImageJ (version 1.53c). The average number of DNA damage foci per nucleus was calculated, and further data analysis was performed using GraphPad Prism 10 (GraphPadPrism Software, Inc.).

### Electron microscopy

For investigation of the ultrastructure of mitochondria, 7.5 × 10^4^ cells per well were seeded into Geltrex™-coated 6-well plates (Greiner Bio-One GmbH). iPSCs were fixed with 3% glutaraldehyde/0.1 M sodium cacodylate buffer at pH 7.2. Cell pellets were washed in fresh 0.1 M cacodylate buffer at pH 7.2 and embedded in 3% low melting agarose. Staining and filtration was performed in 1% osmium tetroxide for 50 min, two washing steps with cacodylate buffer and once with 70% EtOH for 10 min each. Further staining was proceeded in 1% uranyl acetate/1% phosphotunstic acid in 70% EtOH for 1 h. Dehydration of samples was performed by incubation of samples once in 90% EtOH, once in 96% EtOH, thrice in 100% EtOH, and lastly twice in 100% acetone. Stained samples were embedded in low viscosity EMS for polymerization at 70 °C for 48 h. Ultrathin sections (70 nm) of each sample were prepared using a Leica EM UC7microtome (Leica Biosystems GmbH) images were acquired with a JEM-2100 Plus transmission electron microscope (JEOL, Freising, Germany) at 200 KV equipped with an EM-24830 flash CMOS camera system (JEOL, Freising, Germany). Images of ultrastructure were randomized and were categorized into tubular, septae, arched and branched cristae, and the mean percentage of each cristae phenotype was determined.

### Blue native and clear native PAGE

For Blue Native and Clear Native PAGEs, 7.5 × 10^4^ cells per well were seeded into Geltrex^TM^-coated 6-well plates (Greiner Bio-One GmbH). iPSCs were treated according to the defined treatment scheme (see Fig. 1A) and harvested after 48 h of recovery time. Cell pellets were resuspended in 1 ml lysis buffer (210 mM mannitol, 70 mM sucrose, 1 mM EDTA, 20 mM HEPES, 0.1% BSA, 1× protease inhibitor) and incubated on ice for 10 min. Cells were disrupted using a 20 G canula, followed by sequential centrifugation steps at 1000 × *g*, 4 °C for 10 min to remove cell debris and 10,000 × *g*, 4 °C for 15 min to pellet mitochondria. Mitochondrial pellet was resuspended in BSA-free lysis buffer and protein concentration was determined using DC Protein Assay Kit.

For blue native page, 100 µg of mitochondria were solubilized for 1 h on ice using 2.5 g/g of digitonin to protein ratio. Insolubilized material was pelleted at 20,000 × *g*, 4 °C for 20 min. The supernatants were supplemented with loading buffer (50% glycerol, 8 g/g Coomassie to detergent ratio) and loaded onto 3–13% gradient gel and run at 150 V, 15 mA, 4 °C for 16 h. Thereafter, protein complexes were transferred onto PVDF membrane and blocked overnight with 5% milk in TBS-T at 4 °C. For identification of relevant protein complexes, the membrane was decorated using the following antibodies: NDUFB4 (abcam, #ab110243), UQCRC2 (abcam, #ab14745), COXIV (abcam, #ab16056) ATP5A (abcam, #ab14748), OXPHOS cocktail antibody (abcam, #ab110412), Mic60 (abcam, #ab110329), Goat IgG anti-Mouse IgG (abcam, #ab97023) and Goat IgG anti-Rabbit IgG (Dianova, #SBA-4050-05) conjugated to HRP. The chemiluminescent signals were obtained using Pierce™ SuperSignal™ West Pico PLUS chemiluminescent substrate reagent (Thermo Scientific) and VILBER LOURMAT Fusion SL equipment (Peqlab).

For clear native gels, 300 µg mitochondria were solubilized on ice for 1 h with 2.5 g/g digitonin to protein ratio. The samples were centrifuged for 20 min at 20,000 × *g* and 4 °C to pellet insolubilized material. The supernatants were supplemented with loading buffer (50% glycerol, 0.01% Ponceau S) and immediately loaded onto 3-13% gradient gel. Complexes were separated at 150 V, 15 mA for 16 h. To assess complex in-gel activity, the gel slices were incubated in respective buffer solution for several hours at room temperature. For complex I activity, the gel was incubated in 5 mM Tris-HCl pH 7.4, 0.1 mg/mL NADH and 2.5 mg/mL nitro blue tetrazolium chloride (NBT). For complex IV activity, the gel was incubated in 50 mM sodium phosphate buffer pH 7.2, 0.05% DAB and 50 µM horse heart cytochrome *c*.

### MitoTracker™Green FM and TMRM staining

For analysis of mitochondrial morphology and membrane potential, 7.5 × 10^4^ cells were seeded on Geltrex^TM^-coated 3.5 cm MatTek glass bottom dishes (MATTEK) for live-cell microscopy followed by treatment regimen with Dox. On the day of microscopy, cells were stained with MitoTracker^TM^Green FM and Tetramethylrhodamin (TMRM) for 30 min (Thermo Fisher Scientific Inc.). Cells were washed thrice with 1 x PBS and were covered 1 mL mTeSR^TM^ Plus (STEMCELL Technologies Inc.) and 10 µM HEPES buffer was added for buffering oxidation of media. Fluorescence imaging of iPSCs was ECLIPSE Ti2 inverted confocal microscope (Nikon Instruments Inc.), coupled with UltraVIEW®VoX spinning disc laser system (PerkinElmer Inc.) equipped with a 63× oil objective (N.A. 1.2) using Velocity 6.3 software (PerkinElmer Inc.). Z-stack imaging was performed at 37 °C. For analysis of mitochondrial morphology with MitoTracker™Green FM, images were randomized and mitochondrial morphology was categorized as tubular, intermediate or fragmented. For estimation of mitochondrial membrane potential, in Velocity 6.3 software (PerkinElmer Inc.) for each image the background was manually corrected and for each individual cell a region of interest (ROI) was manually defined prior to measurement of TMRM fluorescence intensity. Outliers were identified and removed using ROUT (*Q* = 1) outliers test in GraphPad Prism 8 (GraphPadPrism Software, Inc.). Relative TMRM fluorescence intensity for the untreated control was set to 1.0. Alternatively, cells were seeded on Geltrex™- coated MatTek glass bottom dishes (MATTEK) for live cell microscopy followed by treatment with doxorubicin for 2 h and 48 h recovery. As positive control, iPSCs were treated for 30 min with 10 µM CCCP prior to imaging. On the day of microscopy, cells were stained with MitoTracker™Green FM and Tetramethylrhodamin (TMRM) for 30 min (Thermo Fisher Scientific Inc.). Cells were washed thrice with 1× PBS and were covered 1 mL mTeSR™ Plus (STEMCELL Technologies Inc.) prior to imaging. Live cell fluorescence microscopy was performed with the confocal laser scanning microscope AxioObserver LSM880 (Zeiss) at the Advanced Light Microscopy Core Facility of the medical faculty of the Heinrich-Heine-University (AdLight, Düsseldorf). During imaging, cells were constantly kept at 5% CO_2_ and 37 °C. For each Z-stack the average signal of each pixel was acquired over 8 successive line scans, with a pixel dwell time of 2.06 µs and a pixel size of 0.13 µm. Image processing and analysis were performed with Mitochondrial Analyzer (Version 2.1.0^81^. Based on the control images, the ideal threshold was conducted using the 2D Optimize Threshold command. With the 2D Batch command the defined threshold was applied over all images within one experimental approach. Quantitative analysis of mitochondrial morphology was performed by defining the average Form Factor per mitochondria for each for all conditions, with 1 indicating a round object and values > 1 increased elongation. For each treatment condition, the mitochondrial morphology of at least five images was determined.

### Measurement of mitochondrial ROS

7.5 × 10^4^ human iPSCs were seeded on Geltrex^TM^-coated plates followed by cultivation time of 72 h prior to treatment with 5 or 18 nM doxorubicin for 2 h. As positive control, cells were treated for 1 hour with 0.5 µM Antimycin A prior to analysis. On the day of analysis, cells were detached and stained with MitoSOX (1 µM) for 30 min followed by two washing steps. MitoSOX signal was measured using CyFlow Cube 6 (Sysmex, Germany) flow cytometry machine. For analysis, the percentage of MitoSOX-positive cells was determined.

### Analysis of cellular bioenergetics and energy metabolism

Optimal seeding density and FCCP concentration without toxicity response were tested before starting the Seahorse XF Cell Mito Stress Test assay (Agilent Technologies, Inc). For Seahorse run, iPSCs were pulse-treated with Dox for 2 h followed by 24 h of recovery. Afterward, 3.5 × 10^4^ cells per well were seeded on Geltrex^TM^- coated 96-well Seahorse XF cell culture plates and further recovery of 24 hours was continued. According to manufacturer’s protocol, XF sensor cartridges were hydrated with Seahorse XF Calibrant at 37 °C in a non-CO_2_ incubator overnight. Normal cell culture medium was changed to phenol-free Seahorse XF DMEM (Agilent Technologies, Inc.) containing 10 mM Glucose, 1 mM Pyruvate and 2 mM L-Glutamine (all from Sigma-Aldrich®). OCR was measured in a Seahorse XFe96 Flux Analyzer with Seahorse Wave 2.4 software (Agilent Technologies, Inc). In 3 cycles of 3 min mixing and 3 min recording, 1 µM Oligomycin, 0.125 µM FCCP and 0.5 µM Antimycin/Rotenone AA were injected and OCR was measured. The data were normalized to the cell number using Hoechst normalization assay (Thermo Fisher Scientific Inc.). Relative OCR (pmol/min/cell) was set to 100% of the basal respiration of the control samples.

For Seahorse run in iCMs, 4 × 10^4^ iPS (IMR90)-4 cells per well were seeded on Geltrex^TM^-coated XF24 cell culture microplates (Agilent Technologies, Inc). Untreated iCMs (iCM_Mt_Mt), iCMs treated with 18 nM Dox in iPSC stage (iCM_ 18 nM Dox_Mt), iCMs treated on day 7 with 144 nM Dox (iCM_Mt_144 nM Dox), iCMs treated in iPSC stage with 18 nM Dox, on day 7 of cardiac differentiation with 144 nM Dox (iCM_18 nM Dox_144 nM Dox) and iCMs treated in iPSC stage with 18 nM Dox, on day 7 of cardiac differentiation with 18 nM Dox (iCM_18 nM Dox_18 nM_Dox). On day 9 of cardiac differentiation, live-cell energy metabolism of iCMs was measured using the Mito Stress Test. Prior to the measurement, cardiac differentiation media was exchanged by phenol-free Seahorse XF DMEM (Agilent Technologies, Inc.) containing 10 mM glucose, 1 mM pyruvate and 2 mM L-glutamine (all from Agilent Technologies, Inc). OCR was measured in a Seahorse XFe24 Flux Analyzer with Seahorse Wave 2.4 software (Agilent Technologies, Inc). In 3 cycles of 3 min mixing and 3 min recording, 2.5 μM Oligomycin, 2 μM FCCP and 2.5 μM Antimycin/Rotenone AA (all from Agilent Technologies, Inc) was injected and OCR was measured. The data were normalized to the cell number using Hoechst normalization assay (Thermo Fisher Scientific, Inc.). Relative OCR (pmol/min/cell) was set to 100% for the basal respiration of the control samples.

### Analysis of ATP levels

To determine ATP levels in treated and untreated iPSC, 8 × 10^3^ cells were seeded onto Geltrex™-coated 96-well glass-bottom plates (Greiner Bio-One GmbH). After recovery time, luminescence- based assay CellTiter-Glo® 2.0 Cell Viability Assay (Promega) was performed. Luminescence was measured at CLARIOStar^Plus^ plate reader (BMG Labtech GmbH). The data were normalized to the cell number using the Hoechst normalization assay (Thermo Fisher Scientific Inc.). Relative ATP levels of control cells were set to 1.

For measurement of ATP level in iCMs, 2 × 10^4^ cells were seeded on Geltrex™-coated 48-well plates (Greiner Bio-One GmbH), followed by initiation of cardiac differentiation. Cells were treated according to the indicated conditions. Additionally, treatment with 18 nM Dox in iPSC and iCM stage was included (iCM_18 nM Dox_18 nM Dox). After recovery time, luminescence- based assay CellTiter-Glo® 2.0 Cell Viability Assay (Promega) was performed and cell suspension was transferred into 96-well glass bottom plates in three technical replicates for measurement. Luminescence was measured at the SpectraMax® i3x Multi-Mode Microplate Reader (Molecular Devices GmbH). The data was normalized to the cell number using the Hoechst normalization assay (Thermo Fisher Scientific Inc.). Relative ATP levels of untreated samples were set to 1.

### Analysis of mitochondrial DNA organization

For mitochondrial nucleoid imaging, Z-stack images (20 stacks per image) were recorded using an ECLIPSE Ti2 inverted confocal microscope (Nikon Instruments Inc.), coupled with an UltraVIEW®VoX spinning disc laser system (PerkinElmer Inc.) equipped with a 63× oil objective (N.A. 1.2) using Velocity 6.3 software (PerkinElmer Inc.). Using ImageJ 1.53c^79^, each z-stack image was projected to one z-plane (Maximum projection) followed by separation of each channel and manual thresholding of 8-bit images (Otsu-Threshold). Watershed function was performed and images were further proceeded using a working pipeline in CellProfiler4.2.5^82^. Cells were analyzed with a semi-automated pipeline in CellProfiler. The number of mitochondrial nucleoids per area of cell and the size of mitochondrial nucleoids (µm^2^) for at least 50 cells per condition were determined.

### Mitochondrial morphology and nucleoid imaging in iCMs

For mitochondrial nucleoid imaging in iCMs, following samples were measured: Untreated iCMs (iCM_Mt_Mt), iCMs treated with 18 nM Dox in iPSC stage (iCM_Dox_Mt), iCMs treated on day7 with 144 nM Dox (iCM_Mt_Dox), iCMs treated in iPSC stage with 18 nM Dox and on day7 of cardiac differentiation with 144 nM Dox (iCM_Dox_Dox). Fluorescence Z-stack imaging of iCMs was acquired at ECLIPSE Ti2 inverted confocal microscope (Nikon Instruments Inc.), coupled with UltraVIEW®VoX spinning disc laser system (PerkinElmer Inc.) equipped with a 63× oil objective (N.A. 1.2) using Velocity 6.3 software (PerkinElmer Inc.). Using ImageJ 1.53c, each z-stack image was projected to one z-plane (Maximum projection) followed by separation of each channel and manual thresholding of 8-bit images (Otsu-Threshold). Watershed function was performed and images were further proceeded using a working pipeline in CellProfiler4.2.5^82^. For each treatment condition minimum 50 cells were analyzed with a semi-automated pipeline in CellProfiler. The number of mitochondrial nucleoids per cell and the size of mitochondrial nucleoids (µm^2^) were determined.

Mitochondrial morphology in iCMs was determined by using Mitochondrial Analyzer Plug-in in ImageJ^79^. Based on the control images, the ideal threshold was conducted using the 2D Optimize Threshold command. With the 2D Batch command the defined threshold was applied over all images within one experimental approach. Quantitative analysis of mitochondrial morphology was performed by defining the average Form Factor per mitochondria for each for all conditions, with 1 indicating a round object and values >1 increased elongation. For analysis, the mitochondrial morphology of at least 15 images was determined.

### Beating characterization of human induced-pluripotent stem cell derived cardiomyocytes (iCMs)

For beating analysis, 8 × 10^4^ cells per well were seeded into Geltrex™-coated 12-well plates (Greiner Bio-One GmbH). On day 9 of iCM differentiation, live cell videos of about 20 s were acquired with x20 magnification at Leica DM IL LED Fluo Cellfactory (Leica) using 60 frames per second. All video analyses of beating frequency in the wells were conducted using an automated system applied earlier^44^. The following steps were applied to analyze each video. First, a motion profile was established by placing reference points on the video in a grid pattern, with each point spaced 20 pixels apart. The motion of each reference point was then tracked using the Lucas-Kanade method^83^, which is available in the OpenCV library^84^. This tracking was conducted on a frame-by-frame basis. The movement of each reference point generated a distance profile, indicating how far each point moved from its initial position in the first frame at time t. Next, a Savitzky-Golay filter^85^ was applied to the resulting time-distance curves (motion profiles) to filter out minimal movements caused by noise or potential artifacts like floating particles. This filtering is crucial for distinguishing between the motion of cells and any artifacts, helping to reduce noise in the analysis. Only the motion profiles of grid points within a manually pre-defined area characterized by clearly visible contractions were considered for further analysis. By performing peak analysis on the remaining distance profile curves of the grid points, the endpoint beating frequency was determined. Exemplary distance profiles are shown in the supplementary material (Supplementary Fig. 10).

### Ca^2+^ imaging and analysis

To measure Ca^2+^ oscillations in iCMs, 2 × 10^4^ iPS (IMR90)-4 cells were seeded as single-cell suspension, on Geltrex™-coated cell culture µ-slide 8-well chambers (ibidi GmbH) and cardiac differentiation was induced, accordingly. The indicated Dox-treatment regimens were applied. On day 9 of cardiac differentiation, intracellular calcium levels were measured using the Fluo-4 NW Calcium Assay Kit (Invitrogen, Thermo Fisher Scientific). Staining procedure was followed according to the manufacturer’s instructions. Fast AiryScan microscopy was performed at the Advanced Light Microscopy Core Facility (Ad-Light) with a confocal laser scanning microscope (LSM880) (Carl Zeiss, Jena, Germany) equipped with an AiryScan/AiryScan Fast detection unit. Fluo4-AM was excited with 488 nm and the emission was collected using a bandpass filter 420–480 nm and 495–620 nm. Each movie was acquired using the Fast AiryScan mode without averaging, using a Plan-Apochromat 63×/1.4 NA oil objective (Carl Zeiss, Jena, Germany) and a time interval of 50 ms over 400 frames, resulting in 20 s of recording. The pixel dwell time was 0.86 µs and the pixel size in the images was 0.170 µm. Acquisition was done using a zoom factor of 2×, resulting in an image size of 65.43 µm × 65.43 µm/384 × 384 pixel per image. During Fast AiryScan microscopy iCMs were incubated at 37 °C and 5% CO_2_. In order to analyze Ca^2+^ signal intensities, a threshold was applied for each condition in order to define the border between consecutive Ca^2+^ signal maxima and minima. We considered only peaks of intensity values above 30% of maximal intensity and used these peaks from the respective Ca^2+^ signal curves. For analysis, the number of peaks per 20 s was determined, the average duration between two Ca^2+^ signal peaks, the duration of Ca^2+^ release (average time between Ca^2+^ signal minima and following maximum) and Ca^2+^ uptake (average time between Ca^2+^ signal peak and following minimum) were determined. In three biological replicates, at least 25 regions of interest (ROIs) were measured for each condition.

### Whole genome transcriptome analysis

For the whole genome transcriptome approach, 8 × 10^4^ iPSCs per well were seeded with StemPro™ Accutase™ (ThermoFisher) on Geltrex™-coated 12-well plates (Thermo Fisher Scientific Inc.) and 10 µM ROCK inhibitor Y-27632 2HCl (AdooQ BioScience) for 24 h. When iPSCs reached 80% confluency, cardiomyocyte differentiation was initiated by following a chemically defined protocol. Following treatment conditions were included: iPSC untreated (iPSC_Mt), iPSC treated with Dox (iPSC_Dox), untreated iPSC-derived cardiomyocytes (iCM_Mt_Mt), iCMs treated at iPSC stage (iCM_Dox_Mt), iCMs treated on day 7 of cardiac differentiation (iCM_Mt_Dox) and iCMs treated at iPSC and iCM stage (iCM_Dox_Dox). For iPSCs, cells were harvested 48 h after 2 h pulse-treatment with Dox (respective to IC_30_-value). For iCMs, cells were treated with Dox for 2 h (IC_30_-value respective to cell type) and all iCM samples were harvested on day 9 of cardiac differentiation. For preparation of transcriptomic samples, total RNA was purified using RNeasy Mini Kit (Qiagen). The expression for pluripotent factors POU Class 5 Homeobox 1 (*POU5F1*), Nanog Homeobox (*NANOG*) and cardiac markers Troponin T2 (*TNNT2*), NK2 Homeobox 5 (*NKX2-5*), Myosin Heavy Chain 6 (*Myh6*) and GATA Binding Protein 4 (*GATA4*) was determined with qRT-PCR before further procedure. For each sample the RNA concentration was determined and 20 ng aliquots of RNA were used for transcriptomics analysis. All RNA samples were quantified (Qubit RNA HS Assay, Thermo Fisher Scientific, MA, USA) and quality was assessed by capillary electrophoresis using the Fragment Analyzer and the “Total RNA Standard Sensitivity Assay” (Agilent Technologies, Inc. Santa Clara, CA, USA). Following manufacturer’s protocol, library preparation was performed using ‘VAHTS™ Stranded mRNA-Seq Library Prep Kit’ for Illumina®. As input for mRNA capturing, fragmentation, cDNA synthesis, adapter ligation and library amplification, 20 ng of RNA was used. The bread purified library was normalized and sequencing was performed on the NextSeq2000 system (Illumina Inc. San Diego, CA, USA) with a read setup of 1 × 100 bp. Data were converted from bcl files to fastq files using the BCL Convert Tool (version 3.8.4). Additionally, adapter trimming and demultiplexing was performed with this tool. Quality control was done and submitted together with the read fastq files. The stranded library of single-end mRNA reads was mapped to the human reference genome (ENSEMBL, GRCh38) using the tool HISAT2^86^. Alignments were sorted with samtools^87^ sort option and counts per transcript were received from applying HTSeq^88^. The subsequent analysis was performed in R (version 4.3.0) using the Bioconductor^89^ packages edgeR^90^ and limma/voom^91,92^ to create a count matrix, to filter low expression profiles and to identify differentially expressed genes. Based on the result of multidimensional scaling^92^ which reveals the relationships between the expression profiles of the samples, one replicate was discarded from further analysis to avoid biased results (Replicate number four of Sample iPSC_Dox, Supplementary Fig. 5). Before testing for differential expression, the expression based on the count data was normalized by trimmed mean of M values (TMM^93^) and counts were transformed to lcpm (log counts per million reads). Pairwise comparisons lead to sets of significant differentially expressed genes (Benjamini-Hochberg adjusted *p*-value < 0.05) which were further analyzed by GO-term enrichment using the R packages ClusterProfiler^94^. Heatmaps were generated using the Bioconducter package ComplexHeatmaps^77,78^.

### Statistical analysis

Data is represented as mean ± standard error of mean (SEM). Statistical significance between two groups was determined using unpaired Student’s *t* test. Statistical significance between more than two groups was determined by one-way ANOVA followed by Dunnett’s *post hoc* test. *P-value ≤ 0.05, **P-value ≤ 0.01, ***P-value ≤ 0.001, ****P-value ≤ 0.0001. Data analysis was performed using Microsoft Excel. Data representation and statistical analysis were performed using GraphPad Prism8.

## Supplementary information

Supplementary Information

Supplementary Information

Supplementary movie

Supplementary movie

Supplementary movie

Supplementary movie

## Data Availability

The raw RNASeq dataset generated in this study including sequence reads with quality scores in fastq format study is available on SRA (Sequence Read Archive) included in the BioProject ID: PRJNA1249226 (https://www.ncbi.nlm.nih.gov/bioproject/1249226). Results of statistical analysis as described in the methods part are shown in Supplementary Table 5 showing log fold-change and adjusted p-value for each pairwise comparison per data sheet. This study was deposited in an earlier version as a preprint on BioRxiv (https://doi.org/10.1101/2025.04.18.649376).
