# Supplementary material for: Priming of human iPSCs with doxorubicin causes genome-wide transcriptional reprogramming and functional impairments in iPSC-derived cardiomyocytes
Source: NPJ Regen Med. 2026 Oct 10;11:41. doi: 10.1038/s41536-026-00513-7 (PMC13653920; doi:10.1038/s41536-026-00513-7)
Supplement: Supplementary file 1 [file 41536_2026_513_MOESM1_ESM.pdf]

Supplement

Westerhoff *et al.* "Primimg of human iPSCs with doxorubicin causes genome-wide transcriptional reprogramming and functional impairments in iPSC-derived cardiomyocytes"

Supplementary Figure 1. Influence of doxorubicin pulse-treatment on viability of induced-pluripotent stem cells. A Analysis of cell viability with Sulforhodamine B assay (SRB). iPSCs were pulse-treated with diverse doses of Dox for 2 hours and cell viability was measured 48 hours after treatment with SRB assay. Mean values ± SEM of three independent biological replicates (n=3; N=8) are shown.

Supplementary Figure 2. Influence of Dox pulse-treatment on OPA1 processing in iPSCs. A Quantitative analysis of MitoSOX positive cells after 2 hours Dox pulse-treatment. As positive control, iPSCs were treated with 0.5 µM Antimycin A for 1 hour prior to analysis. Data points shown are the mean ± SEM (n=6). One -way ANOVA with multiple comparisons was performed. * p-value ≤ 0.05, ** p-value ≤ 0.01. **B** Representative electron micrograph of mitochondria from iPSC upon 5 nM and 18 nM Dox treatment. Drawn surroundings of respective mitochondria are shown in the second row. Scale bar 0.1 µm. **C** Quantification of crista per mitochondria 48 h after Dox treatment. Percentage of crista morphology categorized in tubes, septa, arches and branches in iPSCs after Dox treatment. Data show mean ± SEM (n=2; N= 38-50 mitochondria). **D** Blue (left) and clear native (right) PAGE analysis of ETC complex I (CI) and IV (CIV) in control and Dox pulse treated iPSCs. Shown are representative cropped images of blue and clear native PAGEs (blue native PAGE: n=4; clear native PAGE: n=2). **E** Blue Native PAGE gels blotted and probed with indicated OXPHOS complex II (CII), III (CIII), V (CV) and Mic60 antibodies upon pulse-treatment with Dox in iPSCs. Representative cropped images are shown (n=4). Full -length gels/blots of panels **D** and **E** are presented in Supplementary Figure **15C**,**D**. **F** Cells were untreated (Control) or pulse-treated with 18 nM Dox for 2 hours (18 nM Dox) followed by recovery for 48 hours before total cell lysis and Western blot analysis for OPA1 isoforms was performed. As positive control for OPA1 processing, cells were treated with 10 µM Carbonylcyanid-m chlorphenylhydrazon (CCCP) for 1 hour. 10 nM etoposide for 4 hours was used as another control for DNA damage stress. The relative fraction of short OPA1 isoforms (s-OPA1) relative to all OPA1 isoforms (short and long OPA1 isoforms = total OPA1) is given in **G**. Full -length gels/blots of panels **F** are presented in Supplementary Figure **15E. H** Quantitative real-time PCR analysis of mitochondrial encoded genes mitochondrial encoded 16S rRNA (*RNR2*) and NADH-ubiquinone oxidoreductase chain

1 (*MTND1*) in pulse-treated iPSCs. Relative expression levels of three independent biological experiments are shown (n=3; N=3).

Supplementary Figure 3. Influence of Dox treatment on mitochondrial protein expression levels in iPSCs. Mitochondrial protein expression levels in iPSCs pulse-treated with Dox. Cells were untreated (Control) or pulse-treated with 18 nM Dox for 2 hours (18 nM Dox) followed by recovery for 48 hours before total cell lysis and Western blot analysis for mitochondrial proteins was performed. Apparent changes in MRPS25 and MRLP3 levels are indicated by an arrow. Tubulin and GAPDH served as internal standard. Shown are the expression levels of three independent biological replicates. Full-length blots can be found in Supplementary Figure **16 & 17**.

Supplementary Figure 4. Expression level of iPSC-derived cardiomyocytes treated at different time points of cardiac differentiation. A Relative expression levels of pluripotency markers POU Class 5 Homeobox 1 (*POU5F1*), Nanog Homeobox (*NANOG*) and cardiomyocyte marker Troponin T2 (*TNNT2*), NK2 Homeobox 5 (*NKX2-5*), Myosin Heavy Chain 6 (*MYH6*) and GATA Binding Protein 4 (*GATA4*) were measured with quantitative real-time PCR. Following samples were measured: Dox-treated iPSCs (IPSC_Dox), untreated iCMs (iCM_Mt_Mt), in iPSC stage treated iCMs (iCM_Dox_Mt), iCM treated on day7 (iCM_Mt_Dox) and iCMs treated in iPSC stage and on day7 of cardiac differentiation (iCM_Dox_Dox). Expression levels were normalized to the untreated iPSCs control. Means ± SEM of three independent experiments (n=3; N=4) are shown. **B** Measurement of diverse Dox concentrations on the viability of iCMs pre-treated in iPSC stage prior to cardiac differentiation and sham-treated cells. Data points shown represent the mean ± SEM of three independent experiments (n=3; N=8). **C** Representative confocal images of mitochondria in iCMs (iCM_Mt_Mt), iCMs treated in iPSC stage (iCM_Dox_Mt), iCMs treated on day7 of cardiac differentiation (iCM_Mt_Dox) and in double-treated iCMs (iCM_Dox_Dox). Here, HSP60 served as mitochondrial marker. Cell surroundings are marked with dashed lines and zoomed-in regions are marked with a white square. Magnification: x63, scale bar: 10 µm. **D** Quantification of the average Form Factor per mitochondria determined in all treatment conditions (n=3; N=15 images). For statistical analysis, Kruskal-Wallis test with following Dunn's post-hoc test was performed. * P-value ≤ 0.05, *** P-value ≤ 0.001. **EF** Relative basal respiration (**E**) and Spare respiratory capacity (**F**) of the Seahorse run mentioned in Figure **6F-H** are shown for iCMs receiving Dox as pulse-treatment at different stages of cardiac differentiation. Data was normalized to basal respiration of the untreated control- Data shown represents the mean ± SEM of three independent experiments (n=3; N=2-4).

Supplementary Figure 5. Multidimensional scaling of all whole genome transcriptome conditions. Shown are the results of multidimensional scaling revealing the relationship between expression profiles of the samples. One biotechnical replicate was removed for the further analysis (iPSC_Mt) to avoid biased results.

Supplementary Figure 6. Doxorubicin applied prior to cardiac differentiation influences transcriptional response of iCMs towards doxorubicin applied later. A Gene concept network of GO-term enrichments of differentially upregulated genes exclusively shared by late-treated iCMs (iCM_Mt_Dox and iCM_Dox_Dox, all compared to iCM_Mt_Mt). GO-terms of associated genes, number of genes, and logFCs are indicated. **BC** Gene concept network of GO-term enrichments of differentially upregulated (**B**) and downregulated (**C**) genes unique for double-treated iCMs (iCM_Dox_Dox). GO-terms of associated genes, number of genes, and logFCs are indicated. **D** Gene concept network of GO-term enrichments and associated downregulated genes within the exclusive intersection of all treatment conditions in iCMs (iCM_Dox_Mt vs. iCM_Mt_Dox vs. iCM_Dox_Dox). Number of genes and GO-terms are indicated.

Supplementary Figure 7. Time-independent transcriptional response in iPSC-derived cardiomyocytes pulse-treated with doxorubicin revealed partially contrary response. Heatmap of all 35 significantly differentially regulated genes detected within the selective intersection of early treated iCMs (iCM_Dox_Mt), late treated iCMs (iCM_Mt_Dox) and double-treated iCMs (iCM_Dox_Dox)

Supplementary Figure 8. Average gene expression levels of cardiac marker genes in transcriptome conditions and their controls. Shown are the expression levels (gene counts) of cardiac marker genes (*MYH6* (**A**), *TNNC1* (**B**), *CORIN* (**C**), and *ACTN3* (**D**)), glycolytic genes (*PGK1* (**E**), *PDK1* (**F**), *HK1* (**G**), and *GPI* (**H**)), OXPHOS genes (*ND6* (**I**), and *COX3* (**J**)), apoptotic genes (*BAX* (**K**), and *FAS* (**L**)), and fibrosis-related genes (*WT1* (**M**), *COL6A1* (**N**), *COL3A1* (**O**), *DCN* (**P**)) resulted from RNAseq-data of iPSCs and iCMs pulse-treated with Dox for 2 hours with Dox and their respective controls. Each data point represents the expression level of one biotechnical replicate (N=3-4).

Supplementary Figure 9. Volcano plots of iPSCs and iCMs pulse-treated with doxorubicin compared to their untreated control. Shown are pairwise comparisons between iPSCs and iCMs pulse-treated for 2 hours with Dox and their respective controls (iPSC_Mt and iCM_Mt_Mt, respectively). Each data point represents a gene is sorted after the mean logFC as well as the calculated p-value of four biotechnical replicate (N=3-4). In total, 16198 genes were included in this analysis. **ABCD** Volcano plot of the pairwise comparison between Dox-treated iPSCs (**A**), early treated iCMs (**B**), late treated iCMs (**C**), and double-treated iCMs (**D**) to their respective control. Top5 genes as well as genes mentioned in the result part are respectively labeled. The p-value=0.05 as well as the logFC=1 is marked with the dashed line.

Supplementary Figure 10. Visualization of peak analysis of one exemplary reference point in live-cell videos of iCMs. Briefly, analysis was performed with the software CardioVision. During the process, reference points were placed over the video, with a distance of 20 pixels between each point. Each reference point was analyzed and is depicted in the shown graphs. Here, peaks that were marked with an orange X and are counted as beat. **ABCD** Representative graphs of peak analysis are shown for mock-treated iCMs (iCM_Mt_Mt, **A**), iCMs already treated in iPSC stage (iCM_Dox_Mt, **B**), iCMs treated as terminal differentiated cardiomyocytes (iCM_Mt_Dox, **C**), and iCMs receiving Dox treatment in iPSC stage and iCM stage (iCM_Dox_Dox, **D**).

Supplementary Figure 11. Validation of genome-wide transcriptomics data in iCMs including fibrosis by qPCR and Western Blot analysis. A-H Relative gene expression levels of fibrosis (**A, B, C**), cardiac function related (**D, E**) and mitochondrial encoded genes (**F, G, H**) in iCMs. Following samples were measured: Untreated iCMs (iCM_Mt_Mt), iCMs treated with 18 nM Dox in iPSC stage (iCM_18 nM Dox_Mt), iCMs treated on day7 with 144 nM Dox (iCM_Mt_144 nM Dox), iCMs treated in iPSC stage with 18 nM Dox and on day7 of cardiac differentiation with 144 nM Dox (iCM_18 nM Dox_144 nM Dox) and iCMs treated in iPSC stage with 18 nM Dox and on day7 of cardiac differentiation with 144 nM Dox (iCM_18 nM Dox_18 nM Dox). Shown is the average relative expression levels normalized to iCM_Mt_Mt ± SEM of three independent experiments (n=3; N=3). **I** Representative Western Blots showing Collagen III (Col III) protein expression in iCMs short pulse-treated with Dox at defined stages of cardiac differentiation. Vinculin served as internal standard. Shown are two independent biological replicates. Full-length blots of panels **I** are presented in Supplementary Figure **18.**

Supplementary Figure 12. Human iPSC cell lines iPSC11 and iPSC12 are highly sensitive against doxorubicin treatment. AB Viability of iPSC 11 (**A**) and iPSC 12 (**B**) was elucidated 48 hours after pulse-treatment with Dox using MTT assay. Data points represent the mean ± SEM of three independent experiments (n=3; N=3). Dashed line marks the IC_{50}-value. **CF** Representative confocal image of MitoTracker™Green FM-stained mitochondria of control and pulse-treated iPSC11 (**C**) and iPSC12 (**F**). Magnification: x 63, scale bar: 10 µm. Cell surroundings are marked with dashed lines. **DG** Quantification of mitochondrial morphology in iPSC11 (**D**) and iPSC12 (**G**) with the average mean form factor per mitochondria. 10 µM CCCP treatment served as positive control. Data show mean ± SEM (n=3; N=15 images). One-way ANOVA was performed. * p-value ≤ 0.05, ***P-value ≤ 0.001, ****P-value ≤ 0.001. **EH** Quantification of relative ATP levels in iPSC11 (**E**) and iPSC12 (**H**) 48 hours after 2 hours pulse-treatment with Dox using CellTiter-Glo® 2.0 Cell Viability Assay. Data represent the mean ± SEM (n=3; N=6), normalized to respective control. Unpaired Student's t test was performed. * p-value ≤ 0.05, **p-value = 0.01.

Supplementary Figure 13. Expression levels of cardiac tissue samples from a mouse model of acute Dox-induced toxicity. A-F Relative expression levels of metabolic (**A, B, C**) and cardiac markers (**D, E, F**) in available samples [43] from cardiac tissue from mice following acute treatment with doxorubicin (i.p.). Upon acute treatment, mice received 18 mg/kg doxorubicin once and were sacrificed 48 hours after treatment. NaCl application at comparable time points served as control. Shown is the relative mRNA expression normalized to acute NaCl treated mice ± SEM of four independent experiments (n=4).

Supplementary Figure 14. Comparison of whole genome analysis of mice and patient data with iPSC and iCM data. A Heatmap of targeted analysis of genes involved in cardiac differentiation, fibrosis / extracellular matrix remodeling, glycolysis, OXPHOS and apoptosis. Here, iPSC-based data of this study was compared to RNA-seq data from mouse heart tissue of doxorubicin treated mice [42] found with ARCHS4 database research . Average z-score for all four technical replicates of each condition are shown. Coloring for z-scores as indicated. **B** Heatmap of targeted analysis of genes involved in oxidative phosphorylation (OXPHOS, B) in iPSC-based data of this study was compared to RNA-seq data found with ARCHS4 database research where patients underwent doxorubicin treatment[41]. Average z-score for all four technical replicates of each condition are shown. Coloring for z-scores as indicated.

Supplementary Figure 15. Uncropped full-length blots from Figure 2. A Full-length blots of DDR gels shown in main Figure 2B. **B** Full-length blots of apoptosis gels shown in main Figure **2D**. **C** Full-length blots of blue native (left) and clear native (right) gels shown in Supplementary Figure **2D**. **D** Full-length blots of blue native gels shown in Supplementary Figure **2E**. **E** Full-length blots of OPA1 gels shown in Supplementary Figure **2F.** Marker lanes were identified using high contrast images of the respective membranes. Cropped regions are indicated by a black square. As indicated, individual western blotting membranes were cut at expected size ranges prior to incubation with primary antibodies to allow for decorating multiple target proteins per gel/blot. Respective loading controls are grouped accordingly.

Supplementary Figures 16. Uncropped full-length blots from Supplementary Figure 3 – Part 1. A Shown are the full-length gels/blots of mitochondrial proteins in iPSCs shown in **Supplementary Figure 3**. Cropped regions are indicated by a black square. Marker lanes were identified using high contrast images of the respective membranes. As indicated, individual western blotting membranes were cut at expected size ranges prior to incubation with primary antibodies to allow for decorating multiple target proteins per gel/blot. Respective loading controls are grouped accordingly.

Supplementary Figures 17. Uncropped full-length blots from Supplementary Figure 3 – Part 2. A Shown are the full-length gels/blots of mitochondrial proteins in iPSCs shown in **Supplementary Figure 3**. For MTCO1 the signal was quite low and for representation the contrast was linearly increased for the entire image (marked with an asterisk). Therefore, the original and the contrast enhanced full images are shown. Cropped regions are indicated by a black square. Marker lanes were identified using high contrast images of the respective membranes. As indicated, individual western blotting membranes were cut at expected size ranges prior to incubation with primary antibodies to allow for decorating multiple target proteins per gel/blot. Respective loading controls are grouped accordingly.

Supplementary Figure 18. Uncropped full-length blots from Supplementary Figure 11. Shown are the full-length gels/blots of Collagen III (Col III) and Vinculin detected in iCMs shown in Supplementary Figure **11I**. Cropped regions are indicated by a black square. As indicated, individual western blotting membranes were cut at expected size ranges prior to incubation with primary antibodies to allow for decorating multiple target proteins per gel/blot. Respective loading controls are grouped accordingly.

Supplementary Movies 1 to 4. Live-cell videos of iCM beating upon Dox treatment. Representative live-cell videos of iPSCs under different treatment conditions. Videos were acquired at Leica DM IL LED Fluo Cellfactory with 60 frames/second. Representative videos of peak analysis are shown for mock-treated iCMs (iCM_Mt_Mt, **Movie 1**), iCMs already treated in iPSC stage (iCM_Dox_Mt, **Movie 2**), iCMs treated as terminal differentiated cardiomyocytes (iCM_Mt_Dox, **Movie 3**), and iCMs receiving Dox treatment in iPSC stage and iCM stage (iCM_Dox_Dox, **Movie 4**).

Supplementary Data. Excel table including information of log fold-change and adjusted p-value for each gene transcript ID, short description and gene symbol. Each data sheet contains all 16198 genes and the respective statistics for its corresponding pairwise comparison. Significant, up- and downregulated genes can be filtered by colors of the columns (pre-filter: significant (adjusted p-value < 0.05, green), sorted descending by log fold-change (upregulation logFC > 0, blue, downregulation logFC < 0, orange).

Supplementary Figure 1

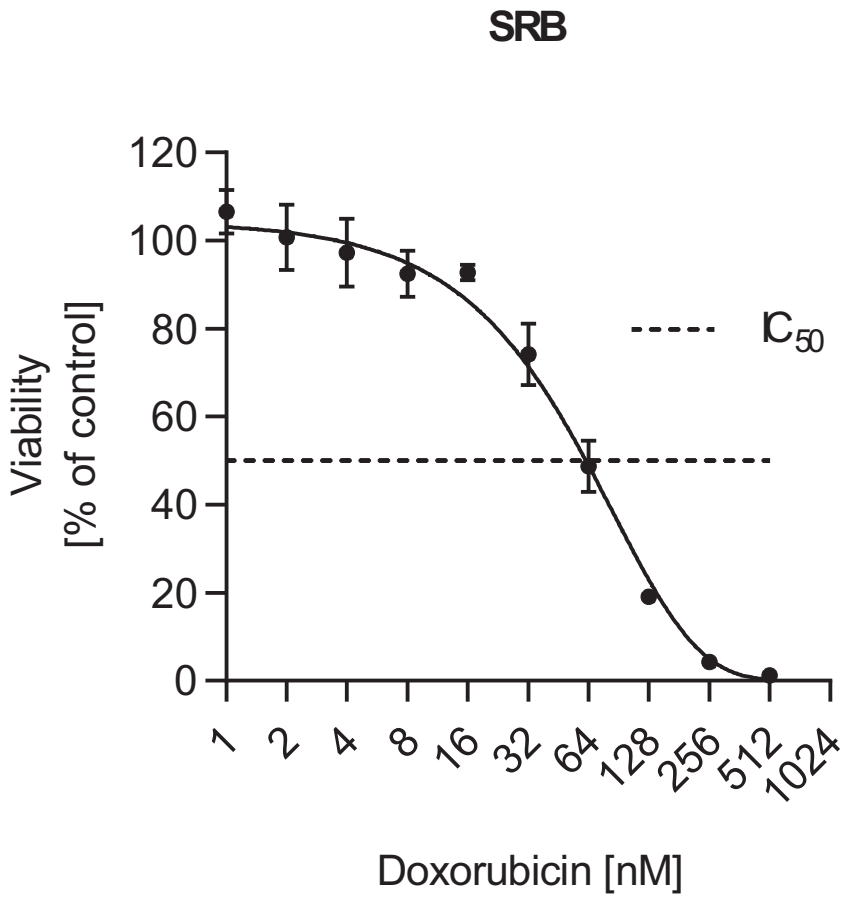

Supplementary Figure 2

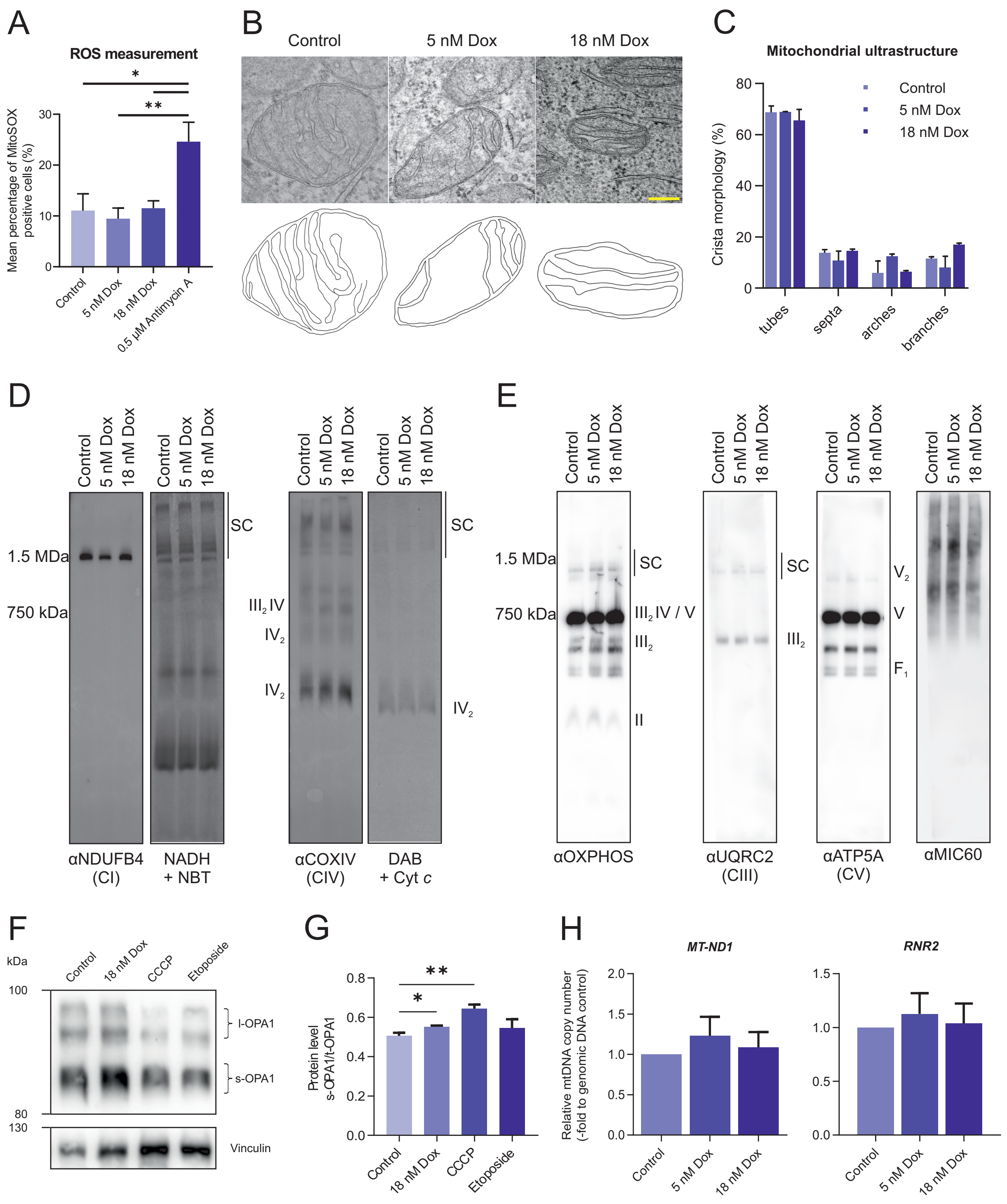

Supplementary Figure 3

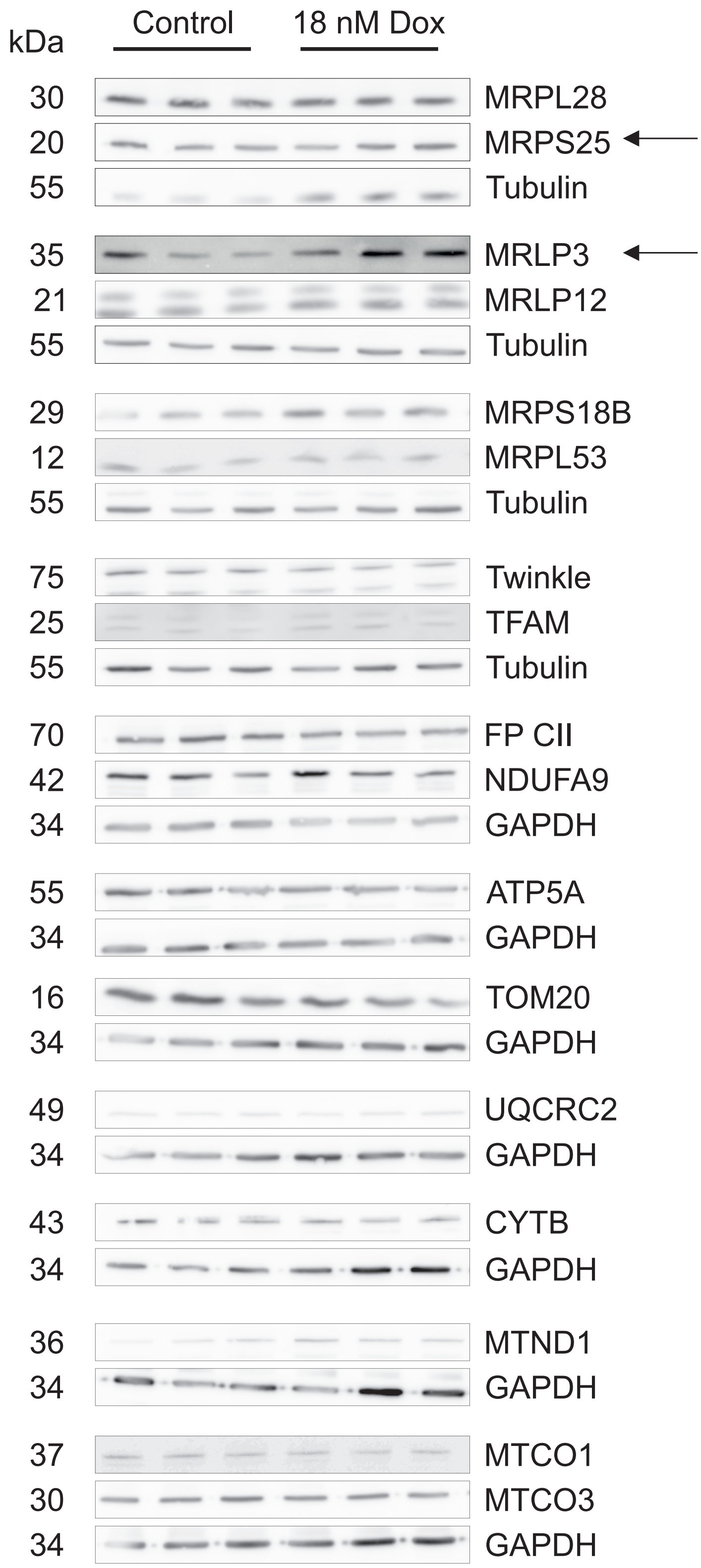

Supplementary Figure 4

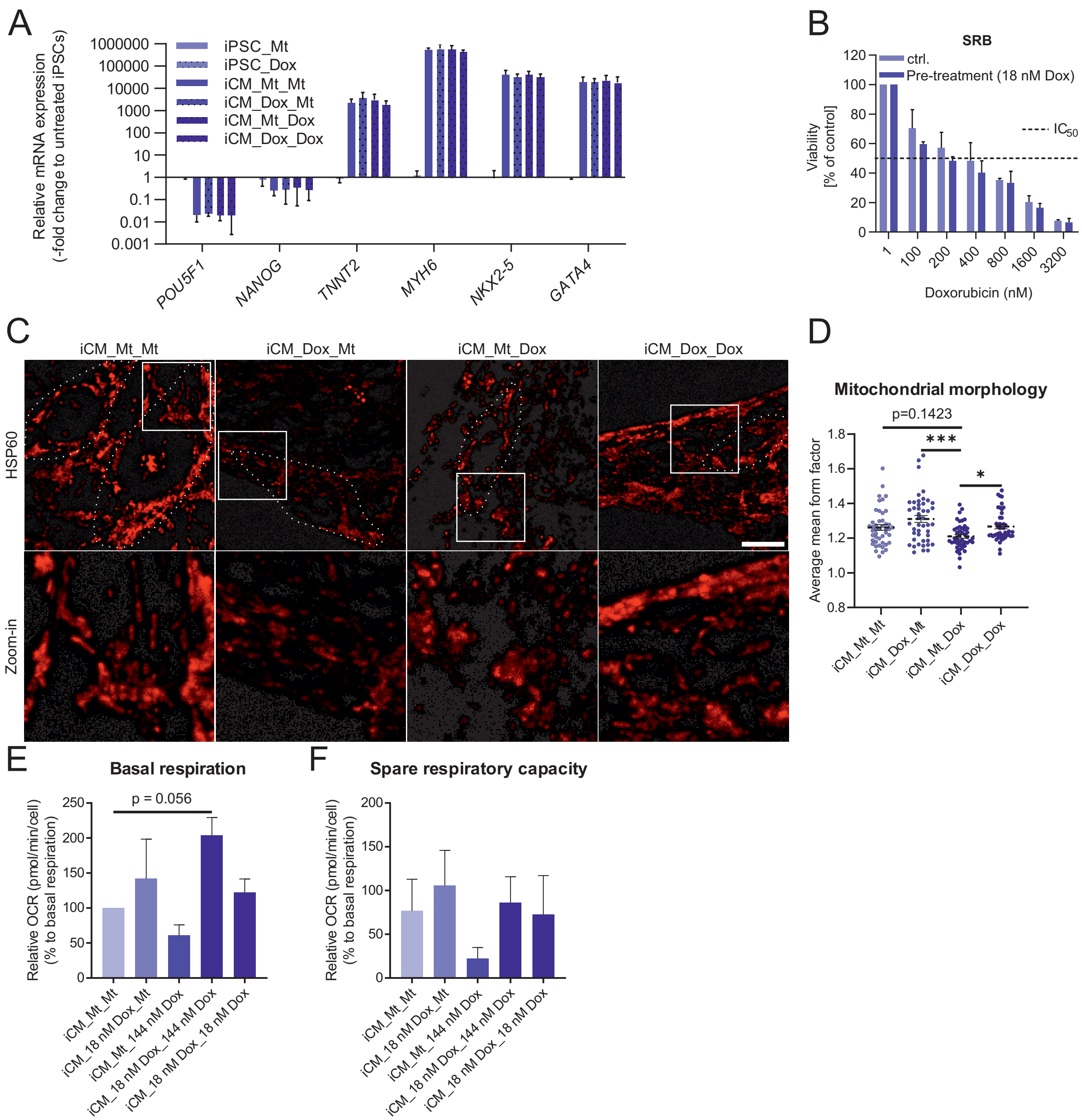

Supplementary Figure 5

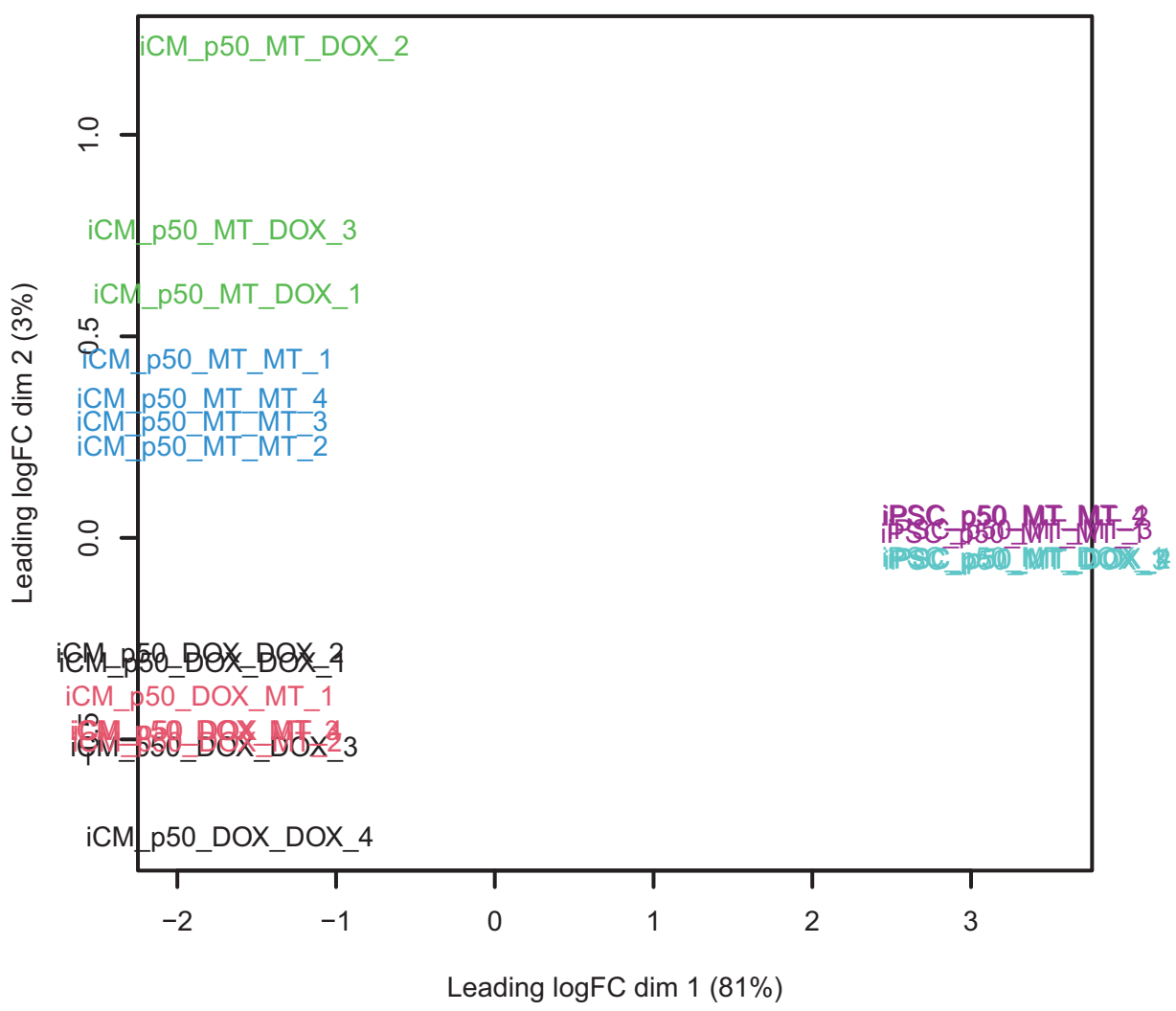

Supplementary Figure 6

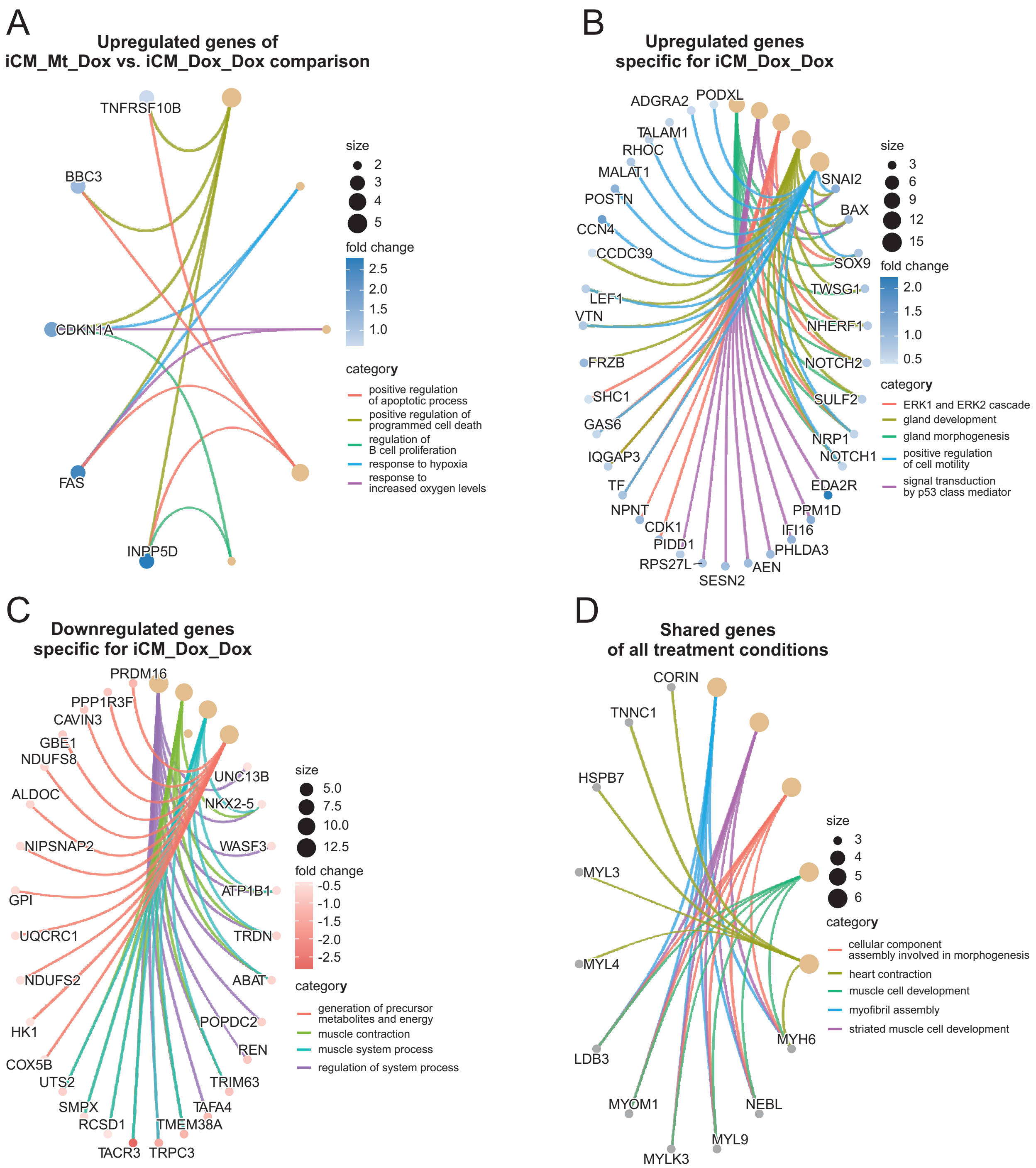

Supplementary Figure 7

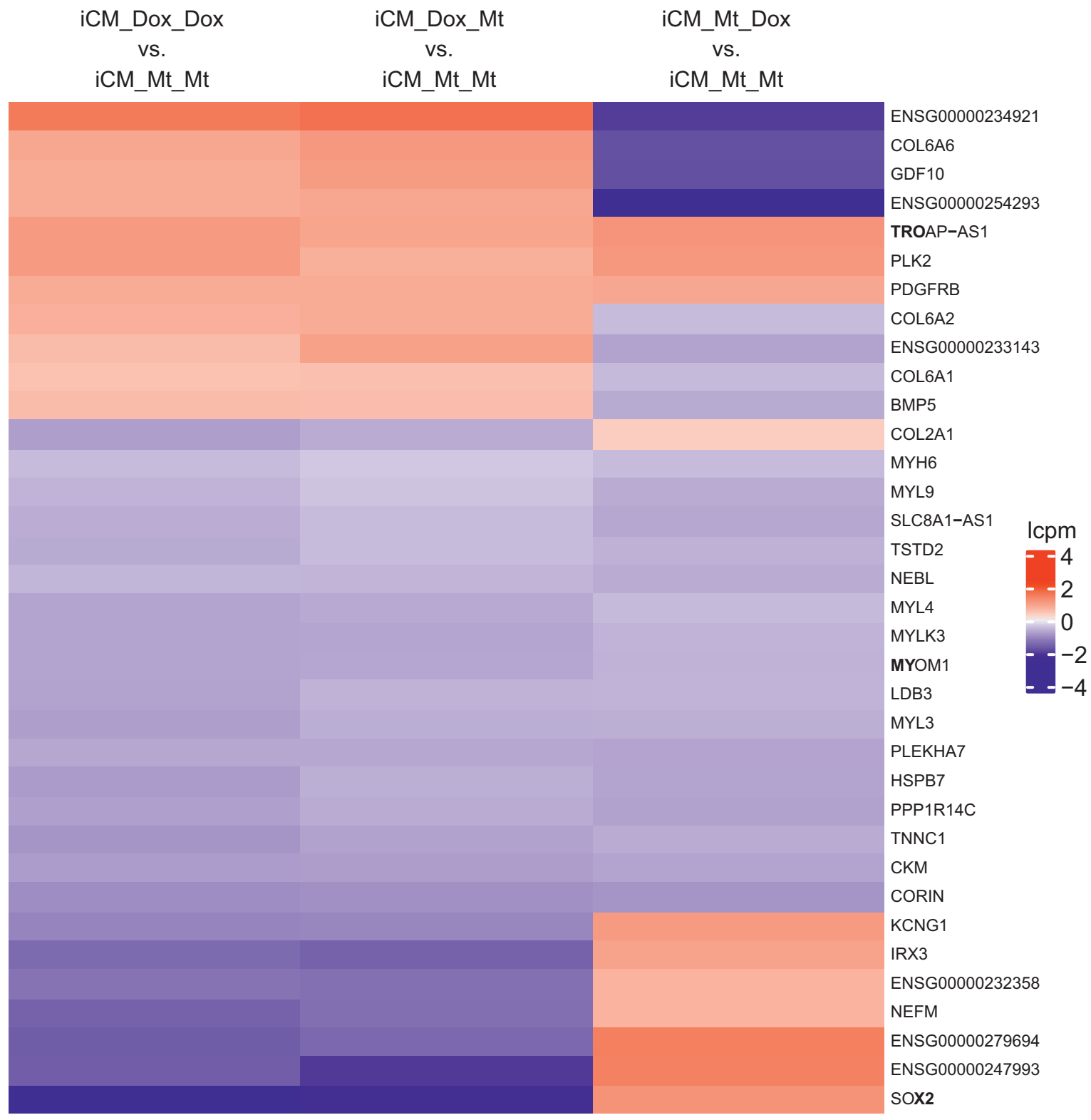

Supplementary Figure 8

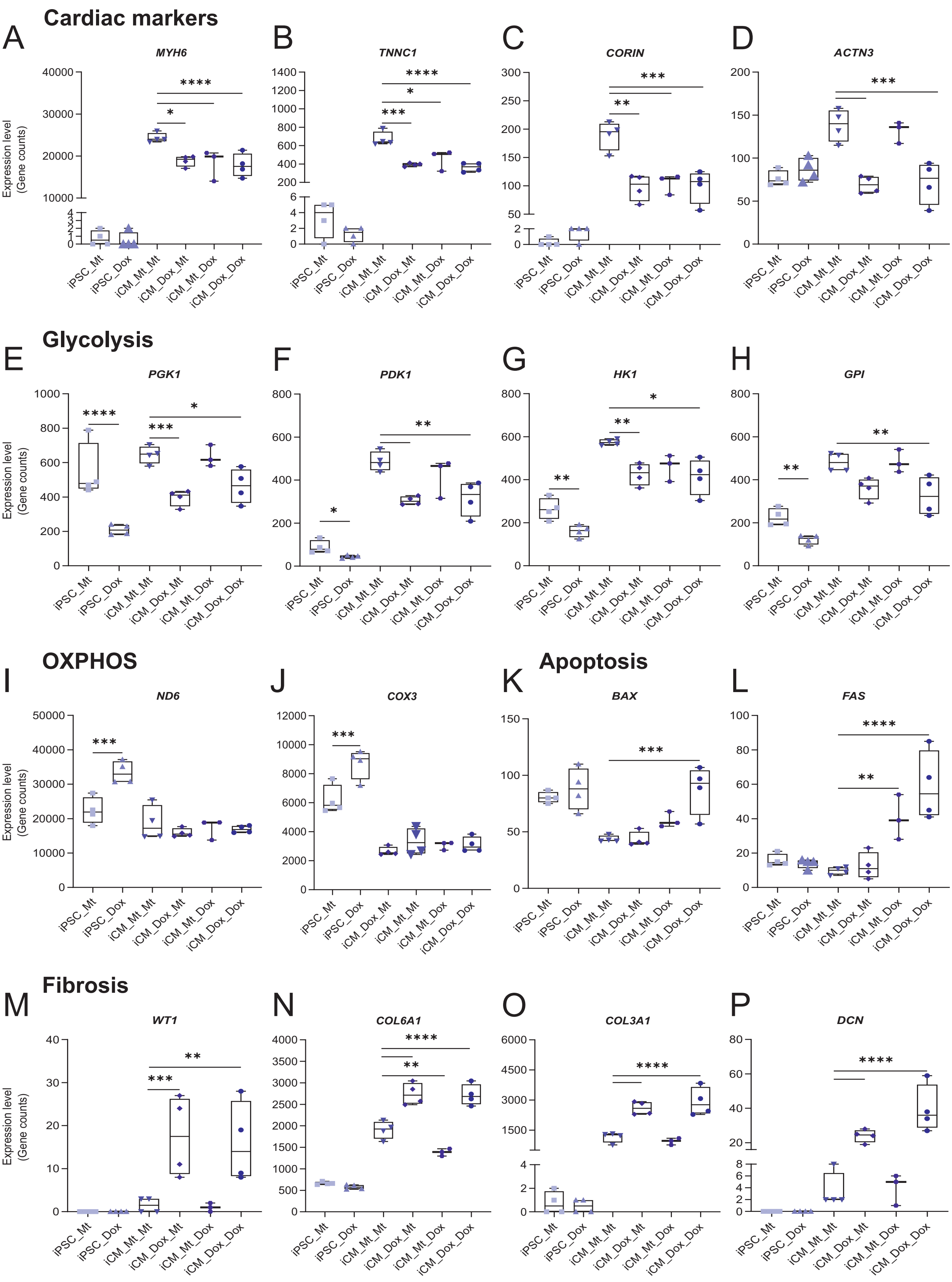

Supplementary Figure 9

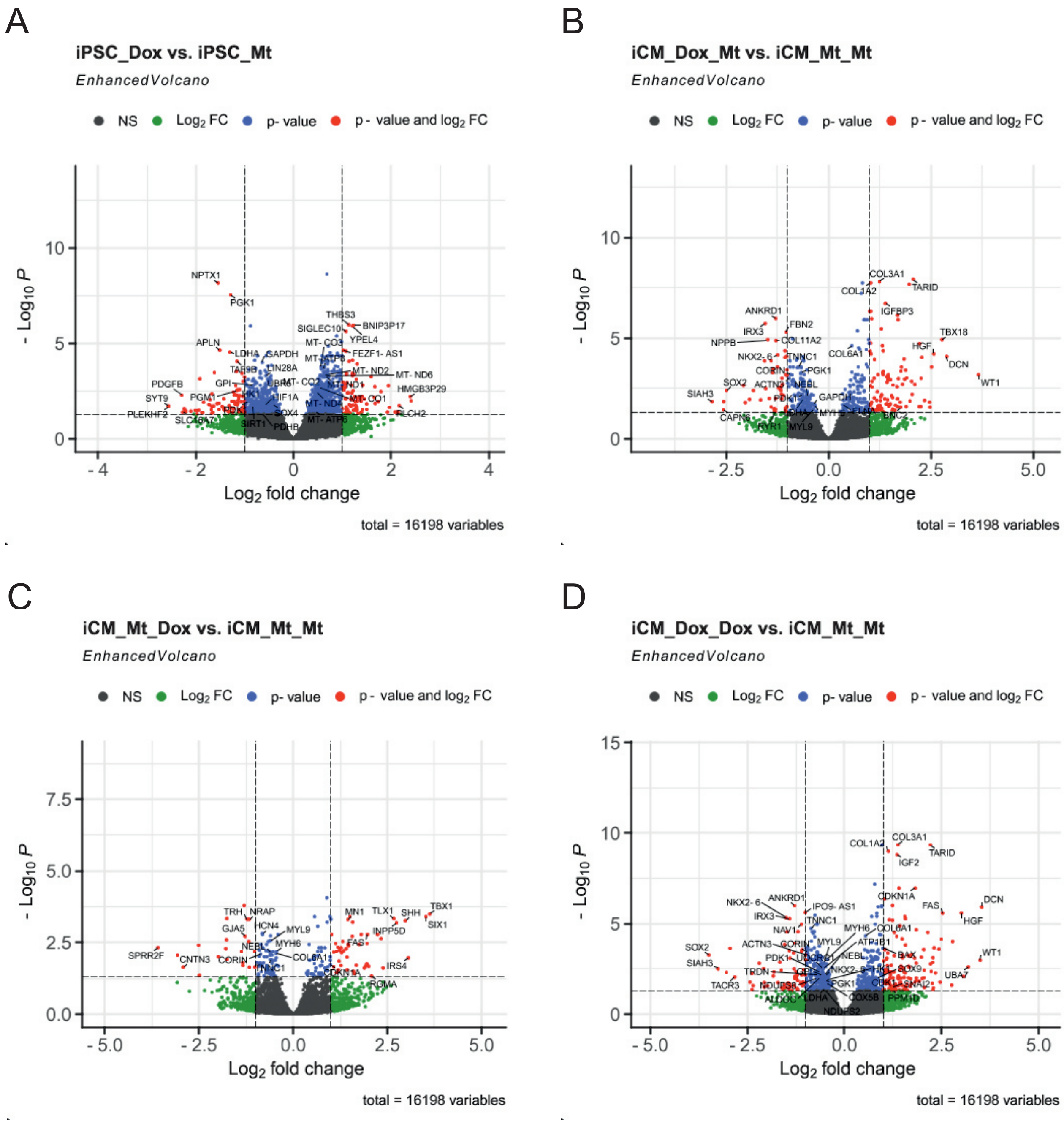

Supplementary Figure 10

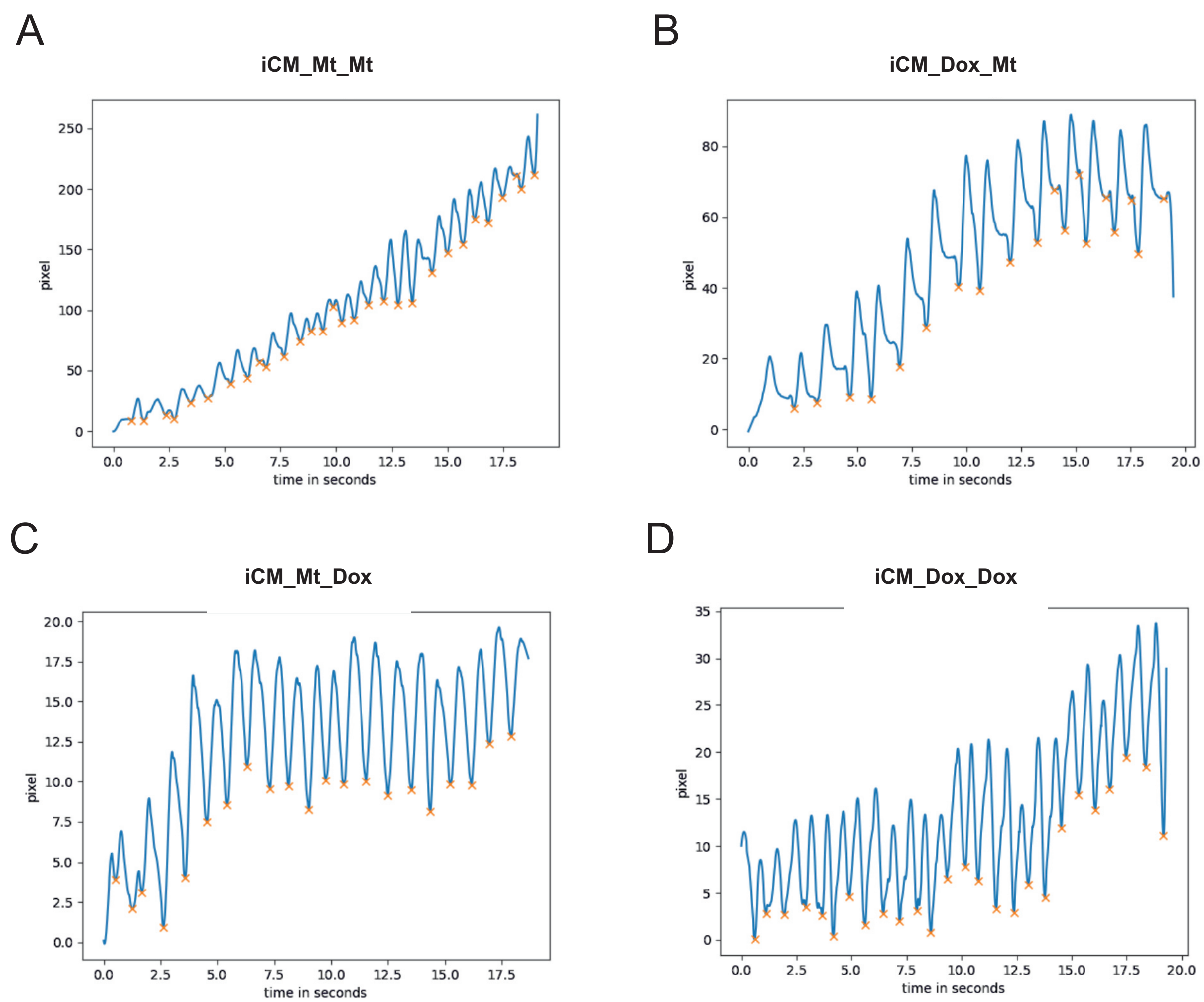

Supplementary Figure 11

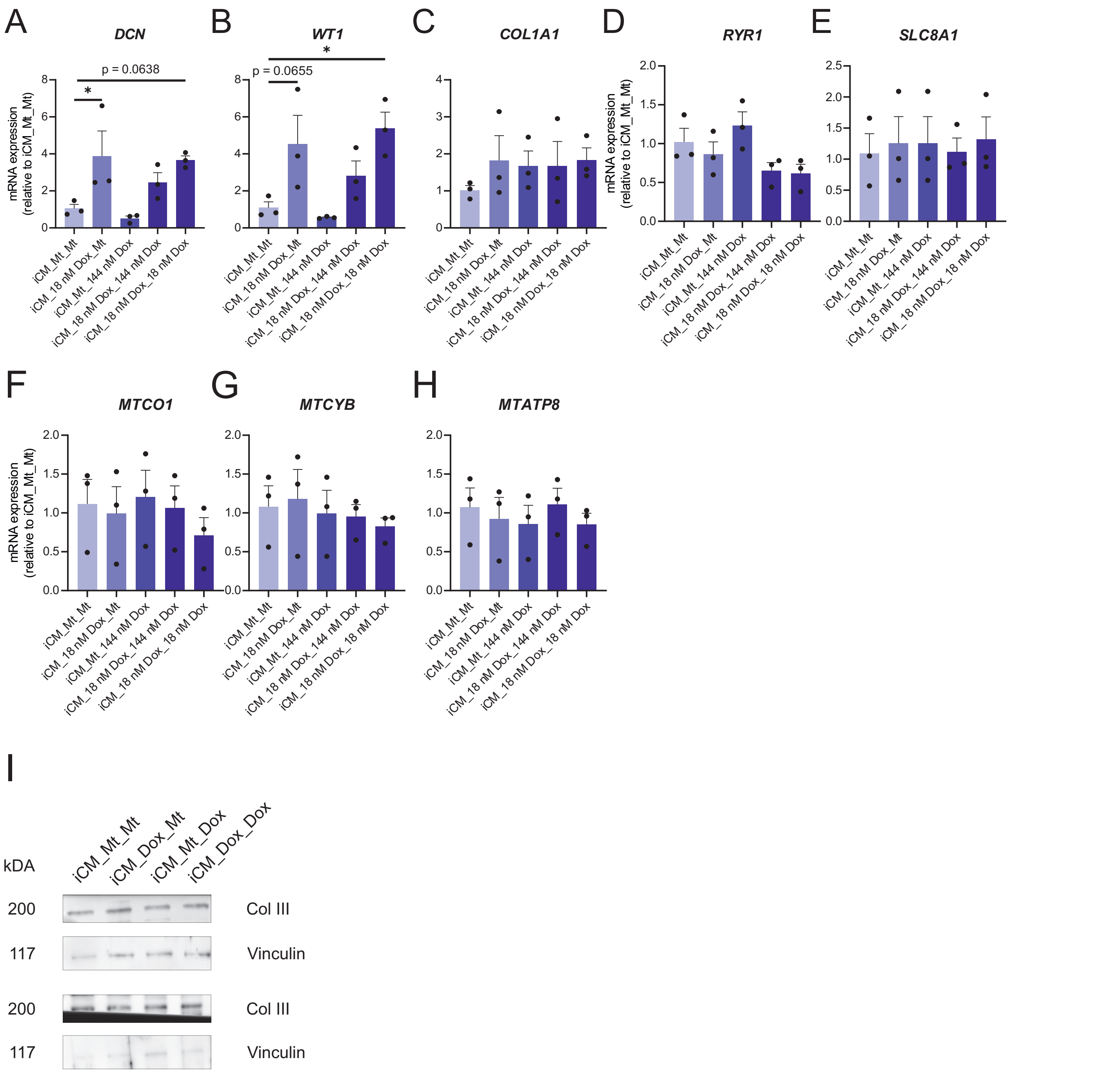

Supplementary Figure 12

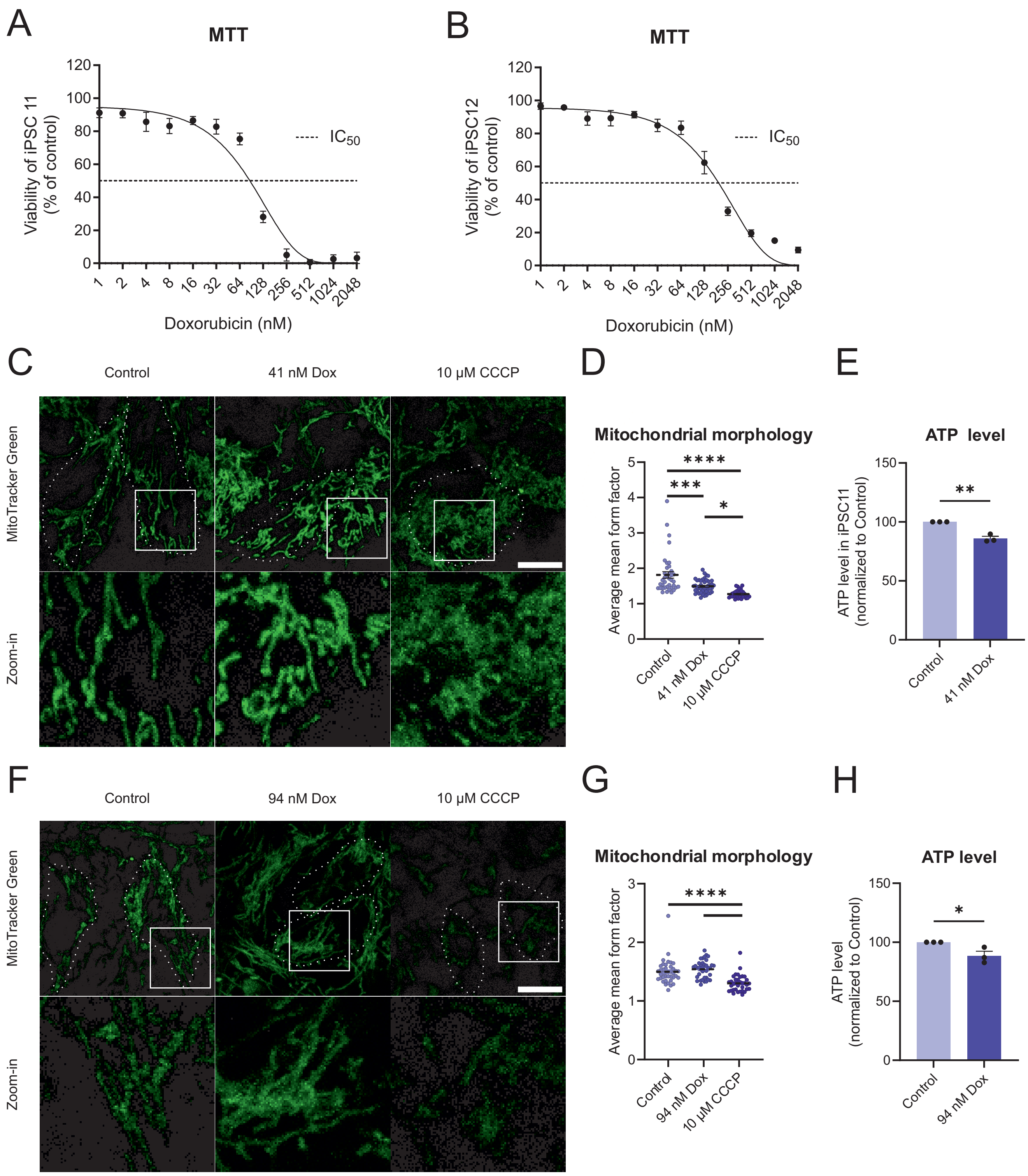

Supplementary Figure 13

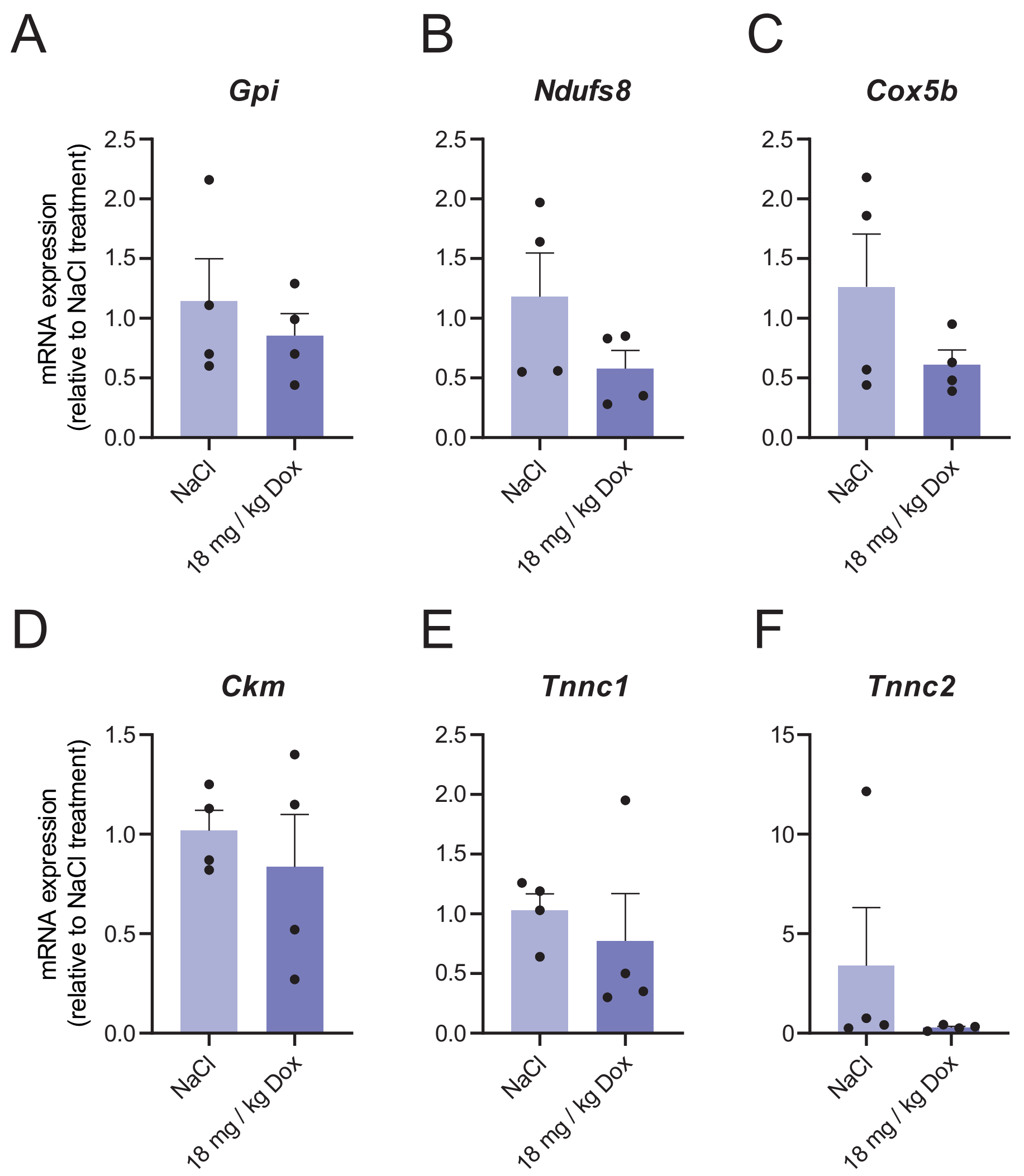

Supplementary Figure 14

A

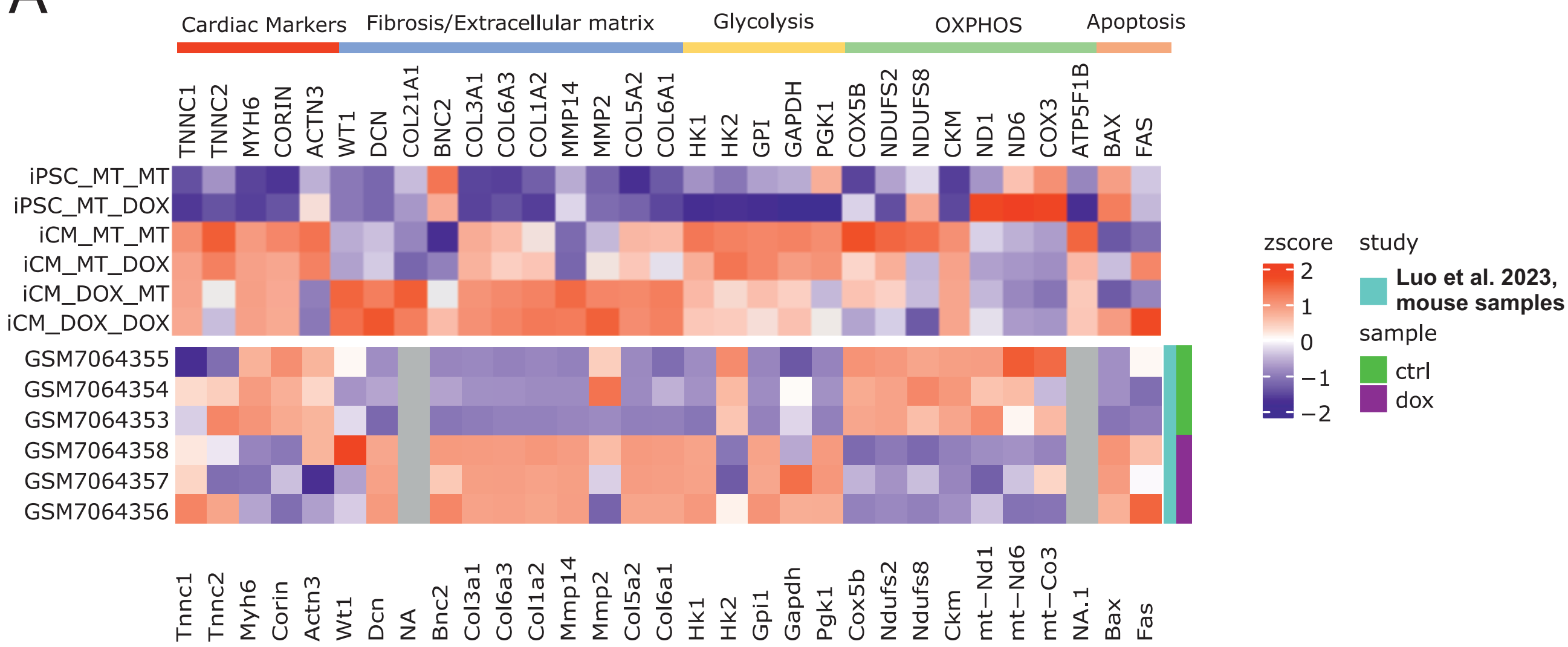

B

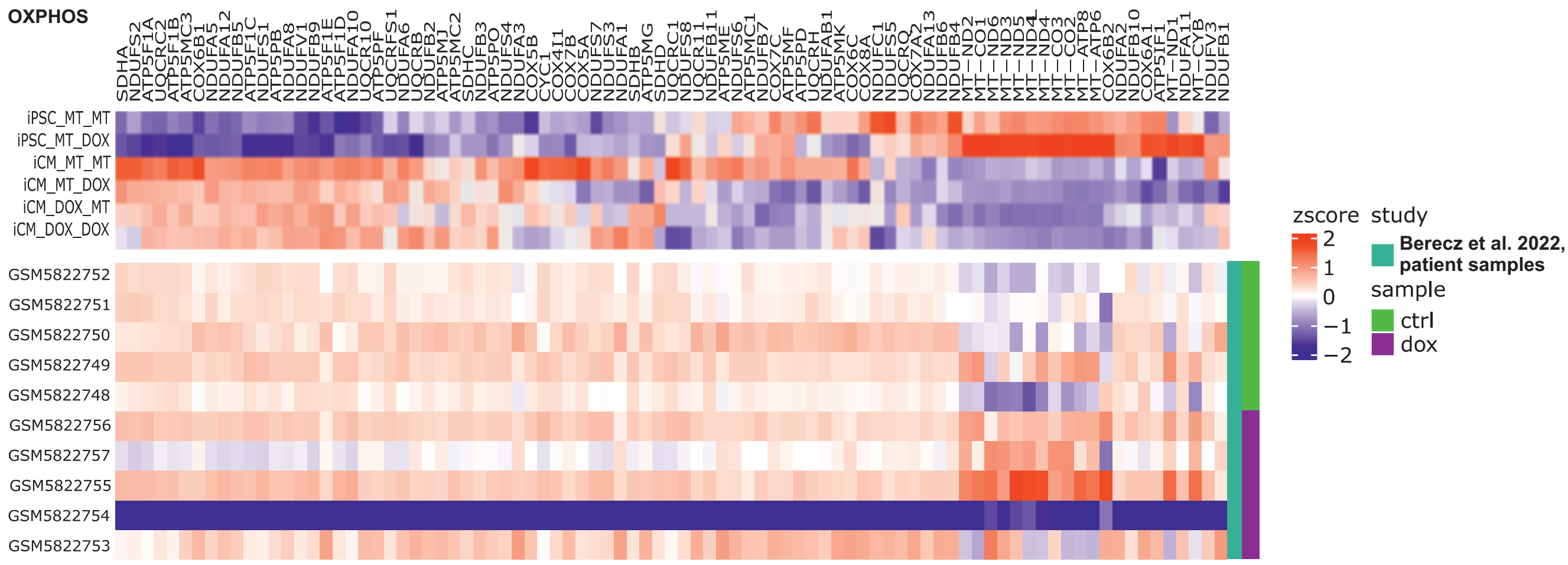

Supplementary Figure 15

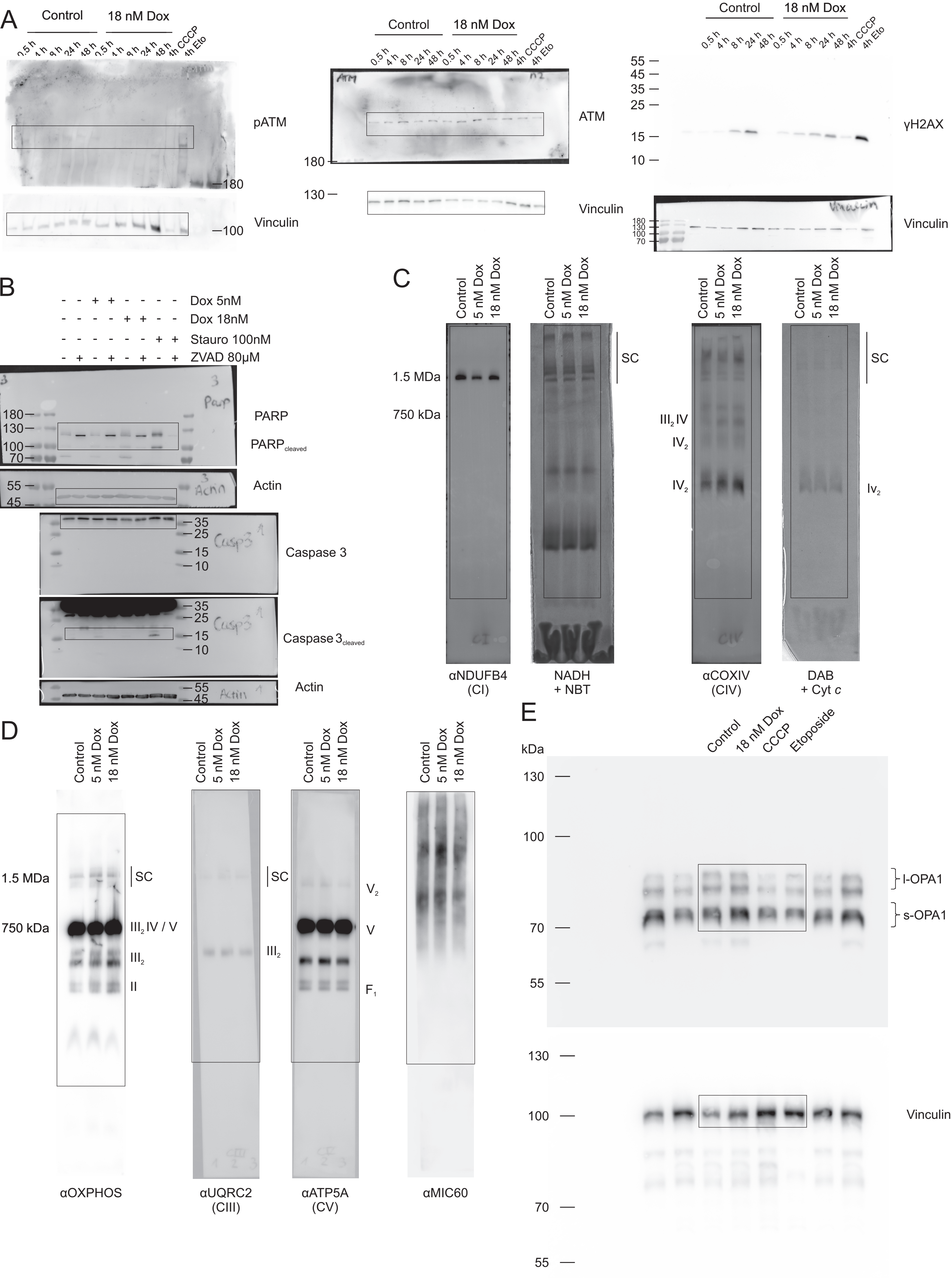

Supplementary Figure 16

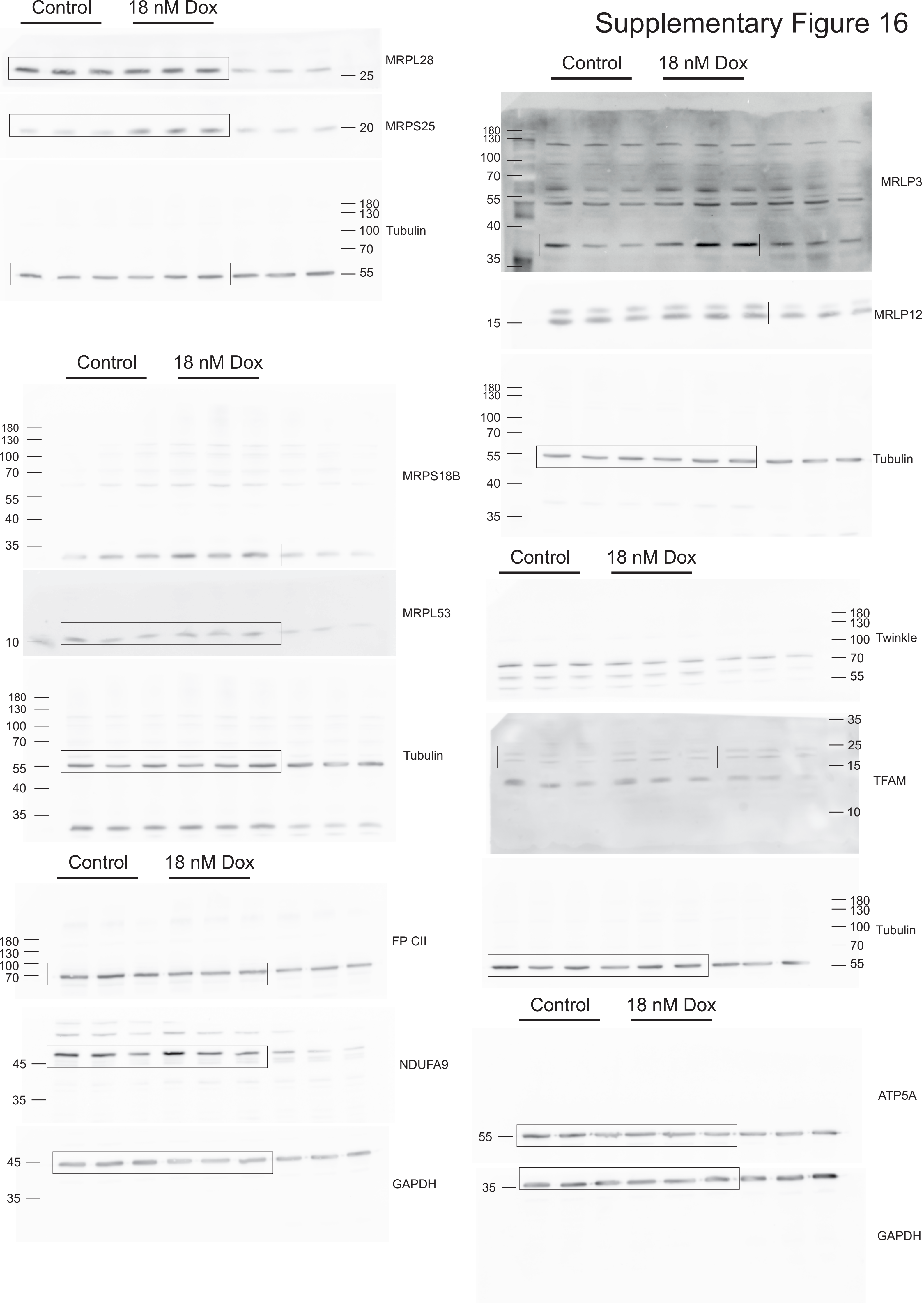

Supplementary Figure 17

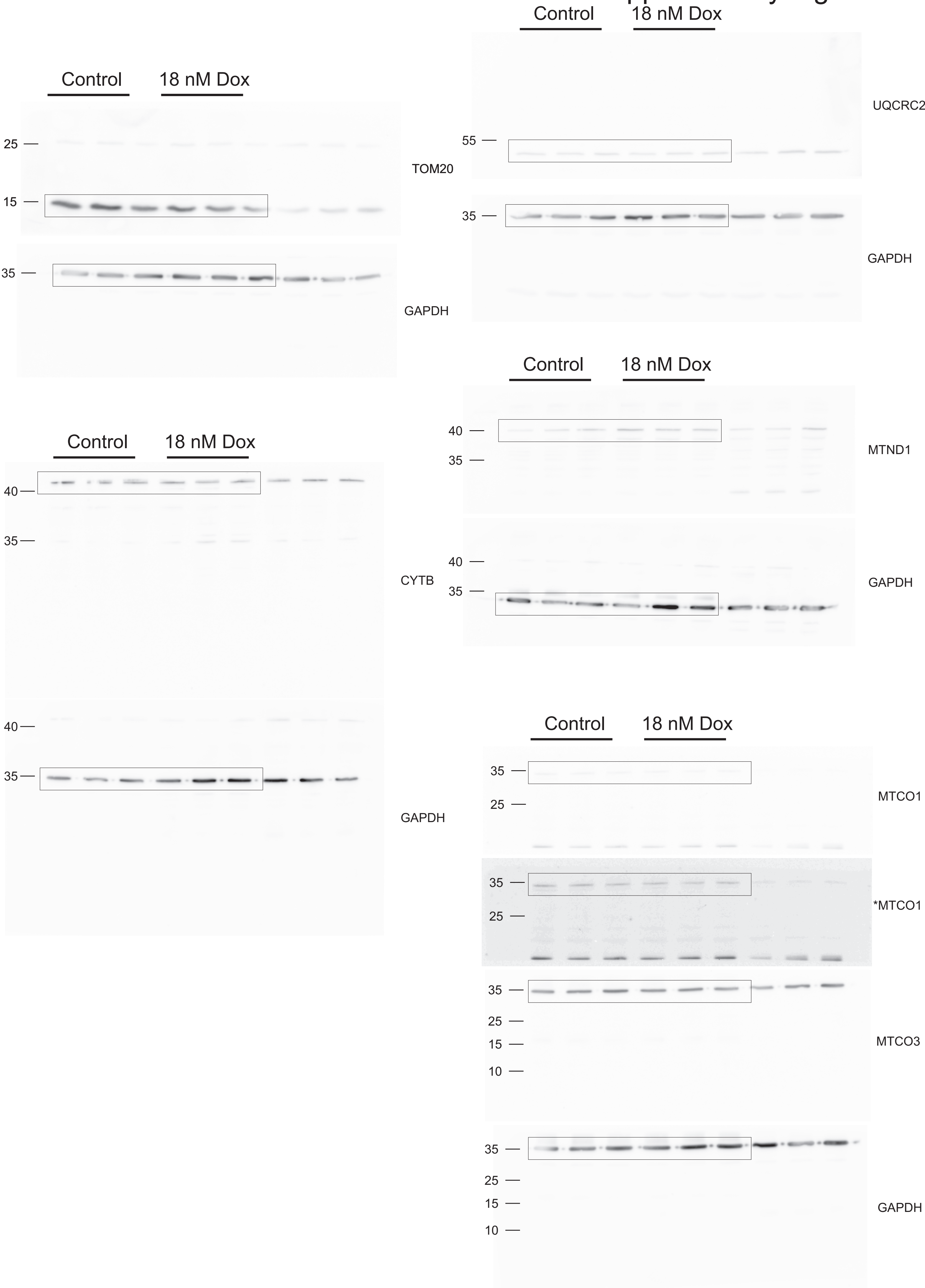

Supplementary Figure 18

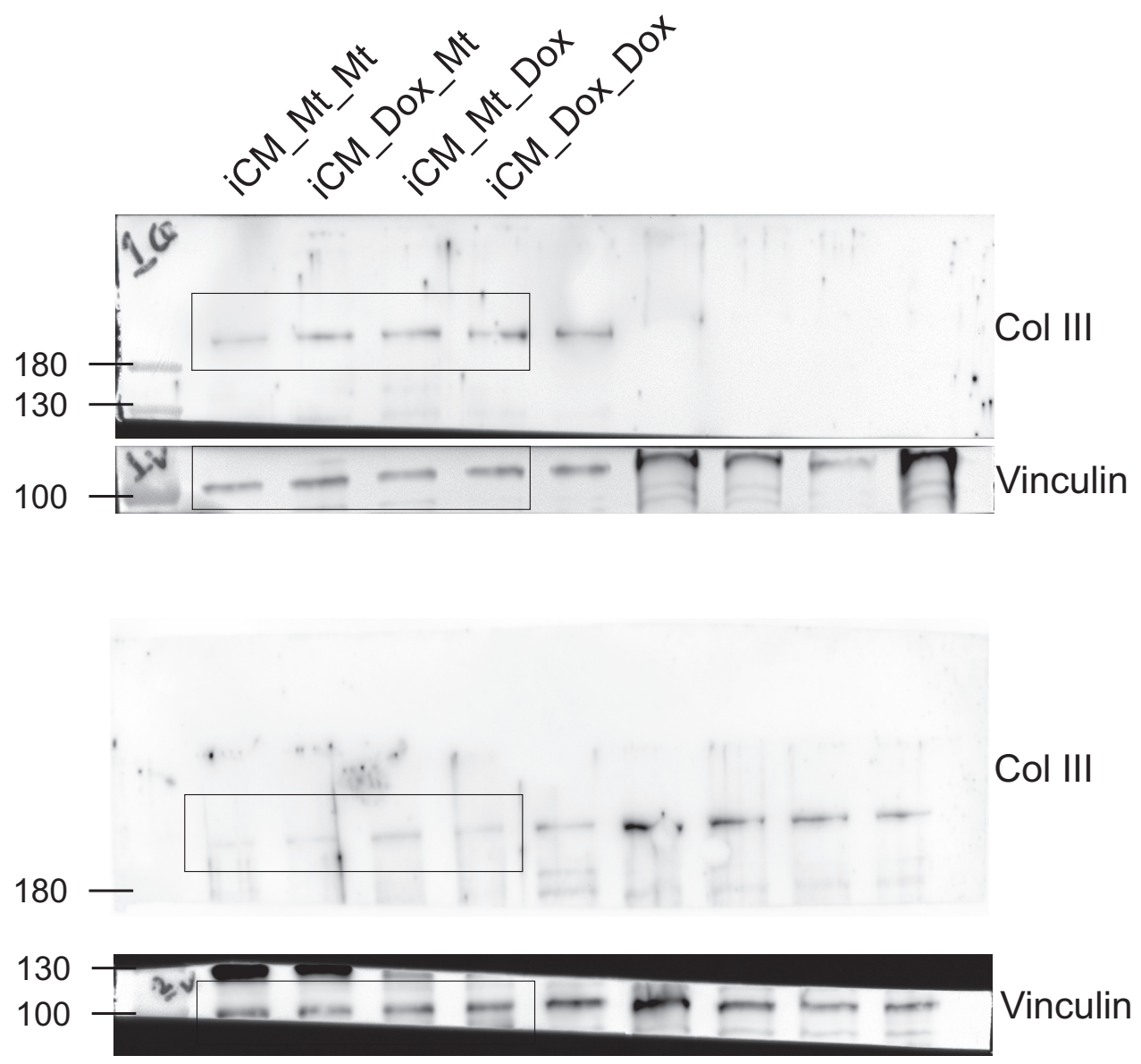

Supplementary Table 1. Primer sequences used for qualitative real-time PCR approaches.

Primer	Forward primer	Reverse primer
GAPDH	CGTAGCTCAGGCCTCAAGAC	GCTGCGGGCTCAATTTATAG
HPRT1	CCTGGCGTCGTGATTAGTG	TGAGGAATAAACACCCTTTCCA
POU5F1	CAGTGCCCGAAACCCACAC	GGAGACCCAGCAGCCTCAAA
NANOG	CAGAAGGCCTCAGCACCTAC	ATTGTTCCAGGTCTGGTTGC
SOX2	GCCGAGTGGAAACTTTTGTCG	GCAGCGTGTACTTATCCTTCTT
TNNT2	TTCACCAAAGATCTGCTCCTCGCT	AACATAAATACGGGTGGGTGCGTG
NKX2-5	AAGTGTGCGTCTGCCTTTCCCG	TTGTCCGCCTCTGTCTTCTCCA
GATA4	GCGGTGCTTCCAGCAACTCCA	GACATCGCACTGACTGAGAACG
MYH6	GGAAGACAAGGTCAACAGCCTG	TCCAGTTTCCGCTTTGCTCGCT
RNR2	AACGATTAAAGTCCTACGTGATC	TCCTTTCGTACAGGGAGGAAT
MtND1	CCACCCTTATCACAACACAAGA	GGTTCGGTTGGTCTCTGCTA
COL1A1	GATTCCCTGGACCTAAAGGTGC	AGCCTCTCCATCTTTGCCAGCA
DCN	GCTCTCCTACATCCGCATTGCT	GTCCTTTCAGGCTAGCTGCATC
MT-ATP8	CACAAACTACCACCTACCTCCC	GGCAATGAATGAAGCGAACAGA
MT-CO1	CCCTAATAATCGGTGCCCCC	TTCAACCTGTTCCTGCTCCG
MT-CYB	CCACCCCATCCAACATCTCC	GCGTCTGGTGAGTAGTGCAT
RYR1	CCATCCTCAACGAGTTCGCCAA	AGAGTTTCCGTGTGAGGTGCAG
SLC8A1	CTGCTTTGTGCTTCCCACAG	GAAAATACGGCGCACTCCCT
WT1	CGAGAGCGATAACCACACAACG	GTCTCAGATGCCGACCGTACAA

Supplementary Table 2. Murine primer sequences used for qualitative real-time PCR approaches in mouse samples.

Primer	Forward primer	Reverse primer
Ckm	ACCCCAACTATGTGCTGAGC	CCATAGCTGAACTGAAAACCACC
Cox5b	GCGTTGTTAGACTCCCACCA	CCACTCCGCGAAGTAACCTT
Gpi	AGGAATGGGGCTGGAGAGAT	CCAGAAGAGGGCATCGGATC
Ndufs8	TAGGGTCAGAGGTCAAGGGG	ATACTTGTACGTTGCCGCCA
Nudc1	AGAACTCCAAGCTATCAGAC	TGAGAACTCCAAGCTATCAG
Tnnc1	GAAGGACGACAGCAAAGGGA	TGTCACCGTCCTTCATGAGC
Tnnc2	GATGATGGAGGGCGTTCAGT	TGTGAGGCAGGGAAAGCAAA
Wt1	CGCCCTACAGCAGTGACAAT	CACTCTCATACCCTGTGCCG

Supplementary Table 3. Number of significantly differentially regulated genes in the indicated pairwise comparisons.

The table presents the number of significantly differentially expressed genes (DEGs) in Dox-treated iPSCs and iCMs versus untreated controls. DEGs are categorized as upregulated, downregulated, or total, based on pairwise comparisons between treated samples and their respective controls. A total of 16,198 genes were analyzed using the voom-limma method, applying the Benjamini-Hochberg correction for multiple testing (adjusted p-value < 0.05). No log fold-change (logFC) threshold was applied. Furthermore, a downstream analysis comparing the significant DEGs resulting from iPSC_Dox vs. iPSC-Mt comparison and the significant DEGs resulting from iCM_Dox_Mt vs. iCM_Mt_Mt resulted in the total sum 1448 DEGs influenced upon Dox treatment, among them 842 DEGs were upregulated and 606 DEGs were downregulated.

Total number of tested genes (16198)	**iPSC_Dox vs. iPSC_Mt**	**iCM_Dox_Mt vs. iCM_Mt_Mt**	**iCM_Mt_Dox vs. iCM_Mt_Mt**	**iCM_Dox_Dox vs. iCM_Mt_Mt**
**Upregulated**	**753**	**233**	**97**	**326**
**Downregulated**	**519**	**193**	**89**	**306**
**Sum**	**1272**	**426**	**186**	**632**

Supplementary Table 4. Number of shared significantly differentially regulated genes in the indicated multi-comparisons.

The table presents the number of shared significantly differentially expressed genes (DEGs) in iCMs each compared to the untreated control. DEGs are categorized as upregulated, downregulated, or total, based on pairwise comparisons between different treatment conditions. Only significantly DEGs identified in a prior comparison between each treatment regimen and its respective control were included. A total of 16,198 genes were analyzed using the voom-limma method, applying the Benjamini-Hochberg correction for multiple testing (adjusted p-value < 0.05). No log fold-change (logFC) threshold was applied.

Total number of tested genes (16198)	**DEGs specifically shared by iCM_Dox_Dox vs. iCM Dox_Mt**	**DEGs specifically shared by iCM_Dox_Dox vs. iCM_Mt_Dox**	**Shared by all treatment conditions**	**DEGs specific for iCM_Dox_Dox**
**Upregulated**	139	12	3	164
**Downregulated**	133	12	16	136
**Contrary regulated**	-	1	16	-
**Sum**	272	25	35	300
